# Sequence‐Based *In‐silico* Discovery, Characterisation, and Biocatalytic Application of a Set of Imine Reductases

**DOI:** 10.1002/cctc.201800607

**Published:** 2018-07-17

**Authors:** Stefan Velikogne, Verena Resch, Carina Dertnig, Joerg H. Schrittwieser, Wolfgang Kroutil

**Affiliations:** ^1^ University of Graz Institute of Chemistry NAWI Graz, BioTechMed Graz Heinrichstrasse 28 8010 Graz Austria

**Keywords:** Amines, Asymmetric Catalysis, Biocatalysis, Imine Reductases, Oxidoreductases

## Abstract

Imine reductases (IREDs) have recently become a primary focus of research in biocatalysis, complementing other classes of amine‐forming enzymes such as transaminases and amine dehydrogenases. Following in the footsteps of other research groups, we have established a set of IRED biocatalysts by sequence‐based *in silico* enzyme discovery. In this study, we present basic characterisation data for these novel IREDs and explore their activity and stereoselectivity using a panel of structurally diverse cyclic imines as substrates. Specific activities of >1 U/mg and excellent stereoselectivities (*ee*>99 %) were observed in many cases, and the enzymes proved surprisingly tolerant towards elevated substrate loadings. Co‐expression of the IREDs with an alcohol dehydrogenase for cofactor regeneration led to whole‐cell biocatalysts capable of efficiently reducing imines at 100 mM initial concentration with no need for the addition of extracellular nicotinamide cofactor. Preparative biotransformations on gram scale using these ‘designer cells’ afforded chiral amines in good yield and excellent optical purity.

## Introduction

Chiral amines form the core structure of numerous natural products and are also present in many synthetic bioactive molecules. According to recent estimates, approximately 40 % of active pharmaceutical ingredients and 20 % of agrochemicals contain a chiral amine moiety.^1^ The paramount importance of this class of substances has led to the development of numerous strategies for their asymmetric synthesis,1b,^2^ of which biocatalytic methods have attracted increased interest in recent years.1a,^3^ The established approaches in this context, such as the kinetic resolution of racemic amines using lipases,^4^ the asymmetric reductive amination of prochiral ketones using transaminases,^5^ and the chemo‐enzymatic deracemisation of amines employing amine oxidases in combination with chemical reducing agents,1a,^6^ have recently been supplemented by biocatalytic imine reduction as a novel, intensively investigated strategy for chiral amine synthesis.^7^ In particular, the discovery in 2010 of natural imine reductases (IREDs)^8^ – an enzyme class capable of reducing cyclic imines at the expense of a nicotinamide cofactor – has sparked immense research efforts directed at identifying and characterising a larger number of these novel enzymes, and applying them in organic synthesis.

Over the last seven years, a large number of imine reductases have been identified and heterologously expressed, and their substrate scope, their kinetic properties, their three‐dimensional structure and their applicability in preparative‐scale reactions have been investigated.^8^, ^9^ Both (*R*)‐ and (*S*)‐selective IREDs are relatively common in bacteria (particularly in actinomycetes) and all known members of this enzyme family share the same overall structure: a dimer formed through reciprocal domain swapping between two monomers that consist of an N‐terminal, cofactor‐binding Rossmann fold motif and a helical C‐terminal domain. The preferred cofactor is generally NADPH, but it has been shown that structure‐guided protein engineering can be used to increase the specific activity of IREDs towards the non‐phosphorylated nicotinamide cofactor (NADH).9a,9n Moreover, it has been found that IREDs are not limited to reducing cyclic imines but that they are also capable of coupling carbonyl compounds and amines in a reductive amination reaction, albeit at often drastically reduced rates.9b,9g–9j,9s,9w,9z While most IREDs seem to rely on spontaneous imine formation from the amine and carbonyl substrates when performing such reductive aminations, a sub‐class of the IRED family, termed ‘reductive aminases’, has been found capable of catalysing the imine formation step as well.9c,^10^ Biocatalytic cascade systems in which IREDs are combined with other enzymes to achieve deracemisation of cyclic amines9o or their asymmetric synthesis from open‐chain precursors9e,9m have also been developed. However, reports on the preparative application of IRED‐catalysed reductions have remained comparably scarce.

The first two members of the IRED family (included for comparison in the present study as IRED‐**A** and IRED‐**I**, see Table 1) have been discovered by Mitsukura and co‐workers in an extensive screening of microbial strain collections.^8^ The cloning and sequencing of the genes encoding these proteins has paved the way for subsequent enzyme discovery efforts focused on identifying homologues of the original IREDs in public databases using a sequence‐based bioinformatics approach. A result of these efforts is the establishment of the *Imine Reductase Engineering Database* (https://ired.biocatnet.de/), maintained by researchers from the University of Stuttgart,9aa,^11^ which currently (version 3, accessed 15 March 2018) contains more than 1400 sequences of putative IREDs. The wealth of sequence information in this database has been used as the basis for detailed bioinformatics analyses,^11^ but only a small fraction of the listed enzymes has actually been studied at the protein level.

**Table 1:** Imine reductases investigated in the present study.

Enzyme	UniProt ID	Source Organism	Partial Sequence Alignment^[a]^	Ref.
IRED‐**A**	M4ZRJ3	*Streptomyces* sp. GF3587	119 GAIMIT 124 … 170 LYDVSLLGLMWG 181	9ag, 9ah
IRED‐**B**	Q1EQE0	*Streptomyces kanamyceticus*	134 GAILAG 139 … 185 LYDAAGLVMMWS 196	9ad, 9ag
IRED‐**C**	W7VJL8	*Micromonospora* sp. M42	120 GGIMAV 125 … 172 LHDVALLSAMYG 183	this work
IRED‐**D**	V7GV82	*Mesorhizobium* sp. L2C089B000	115 GGIMAV 120 … 165 LYDISLLTGMYG 176	this work
IRED‐**E**	J7LAY5	*Nocardiopsis alba*	122 GAIMAT 127 … 172 LFDLALLSGMYT 183	this work
IRED‐**F**	V6KA13	*Streptomyces niveus* NCIMB 11891	121 GAVYAV 126 … 171 LYDVALLSGMYG 182	this work
IRED‐**G**	L8EIW6	*Streptomyces rimosus* ATCC 10970	128 GAIMVP 133 … 179 VYDLAMLSFFYS 190	this work
IRED‐**H**	I8QLV7	*Frankia* sp. QA3	119 GAIMTT 124 … 169 LYDVALLGLMWS 180	this work
IRED‐**I**	M4ZS15	*Streptomyces* sp. GF3546	117 GGVQVP 122 … 167 MYYQAQMTIFWT 178	9ac
IRED‐**J**	D2PR38	*Kribbella flavida* DSM 17836	120 GGVMIP 125 … 170 LMYQAQLDVFLT 181	9x
IRED‐**K**	D2AWI4	*Streptosporangium roseum* DSM 43201	118 GGVQVP 123 … 168 LFYQIGMDMFWT 179	9ae
IRED‐**L**	K0F8R0	*Nocardia brasiliensis* ATCC 700358	121 GGVMSA 126 … 171 VYYQALLTIFHP 182	this work
IRED‐**M**	K0K4C6	*Saccharothrix espanaensis* ATCC 51144	114 GGVMVP 119 … 164 LFYQAQLDFFLT 175	this work
IRED‐**N**	J7YM26	*Bacillus cereus*	135 GGVQVP 140 … 185 LYYQIQMDIFWT 196	9v

[a] Highlighted are the ‘proton donor’ residues D187 (IRED‐**B** numbering; red) and Y169 (IRED‐**I** numbering; yellow), the hydrophobic flanking residues 137 and 191 (IRED‐**B** numbering; purple),9ad and residues P139 and F194 (IRED‐**B** numbering; blue and green, respectively), which have been shown to be conserved among (*S*)‐selective IREDs.^11^

Herein, we report the sequence‐based *in‐silico* discovery and characterisation of eight novel imine reductases along with the investigation of six known IREDs, all of which are also contained in the *Imine Reductase Engineering Database*. Moreover, we report on the application of these enzymes in the asymmetric reduction of cyclic imines, employing an alcohol dehydrogenase as auxiliary enzyme and isopropanol as co‐substrate for cofactor regeneration. Finally, we demonstrate that by employing an *E. coli* strain that co‐expresses the alcohol dehydrogenase and a suitable IRED, preparative‐scale reductions of imines at elevated substrate concentration are possible without the need for addition of NADP^+^.

## Results and Discussion

### Sequence‐based *In‐silico* Enzyme Discovery

When we started our investigations in late 2013, the *Imine Reductase Engineering Database* had not yet been established. We therefore followed our own sequence‐based approach for the *in‐silico* discovery of putative IREDs, which we based on overall protein sequence homology to the four confirmed IREDs known at that time (IREDs **A**, **B**, **I** and **K**; see Table 1) and on the presence of specific active‐site key residues. The first X‐ray crystal structure of an IRED (Q1EQE0, IRED‐**B**; PDB 3zgy, 3zhb) had just been solved and published by Grogan and co‐workers a few months earlier, and their results suggested that an aspartic acid residue, D187 (IRED‐**B** numbering), was crucial for the imine reductase activity of the protein, supposedly playing a role in substrate protonation.9ad A tyrosine residue, Y169 (IRED‐**I** numbering), is present in the corresponding position of the (*S*)‐selective IREDs **I** and **K** and can be assumed to serve the same function as D187 in the (*R*)‐selective Q1EQE0 enzyme. On the basis of this limited structural and functional information we decided to search the UniProt database for protein sequences that (*i*) scored an E‐value of ≤10^−50^ in a protein BLAST search using the sequences of IREDs **A**, **B**, **I**, or **K** as template, (*ii*) contained a full‐length Rossmann‐fold domain with a complete GxGxxG consensus sequence, hence excluding truncated sequences, and (*iii*) featured an acidic residue (Asp, Glu or Tyr) in position 187 (IRED‐**B** numbering). Since Grogan and co‐workers suggested that the flanking of D187 in Q1EQE0 by two apolar residues (L137 and L191) might be instrumental in raising the p*K*_a_ value of the D187 side chain, resulting in it being protonated under physiological conditions, we also made the presence of such apolar flanking residues a selection requirement when the ‘proton donor’ residue was Asp or Glu. The 215 hits identified using these search criteria were narrowed down to 182 by removal of duplicates (identical database entries found by more than one of the four BLAST searches) and of redundant sequences (database entries having a different accession code but identical sequence). These 182 candidates were aligned using the Clustal Omega online tool and arranged into a phylogenetic tree by the neighbour‐joining method.

**Figure 1:**
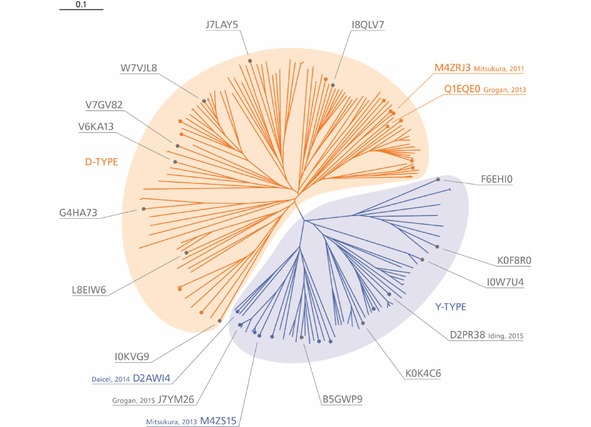
Phylogenetic tree of the putative imine reductases identified in this work. Enzymes reported in the literature before the start of our investigations are shown as coloured dots with coloured labels. Enzymes identified and characterised by other research groups during the course of our studies are shown as coloured dots without labels. Novel IREDs chosen for investigation in the present work are shown as grey dots with labels. The 6‐digit alphanumerical codes are the UniProt identifiers of the respective protein sequences. The scale bar represents 0.1 variations per amino acid residue.

The phylogenetic tree (Figure 1) shows a clear separation into two major branches: the ‘D‐type’ branch with a higher overall homology to confirmed IREDs **A** and **B**, featuring an aspartic acid (or, in two cases a glutamic acid) residue in position 187 (IRED‐**B** numbering), and the ‘Y‐type’ branch with a higher overall homology to IREDs **I** and **J** and a tyrosine residue in the respective position. The D‐type branch is the larger one of the two, accommodating 116 of the 182 candidate sequences identified (64 %). Scheller *et al*. have observed a similar division into two superfamilies of different size in a sequence similarity network analysis of the first version of the *Imine Reductase Engineering Database*.9aa Figure 1 also shows that the sequence space of both major branches of our phylogenetic tree has been explored only in small parts and with an unbalanced distribution of the characterised enzymes.

**Figure 2:**
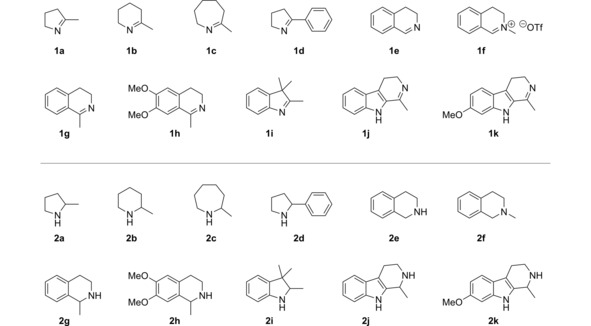
Imines **1** and corresponding amines **2** investigated in the present study.

We randomly selected 15 novel enzyme candidates^12^ from all main sub‐branches of the phylogenetic tree for expression and experimental characterisation, along with the four known IREDs **A**, **B**, **I**, and **K**. The genes encoding these 19 proteins were ordered as linear, synthetic DNA double‐strands, subcloned into pET28a(+) and expressed heterologously in *E. coli* BL21 (DE3), using terrific broth (TB) as growth medium and IPTG (1 mM) for induction. Under these conditions, four enzymes (B5GWP9, F6EHI0, I0KVG9, I0W7U4) were expressed only in insoluble form, while for the gene encoding G4HA73 several attempts of subcloning failed and an expression construct was hence never obtained. These five candidates were therefore excluded from all further investigations. The 14 enzymes expressed in soluble form (listed in Table 1) were tested for IRED activity using wet whole cells as biocatalyst and 2‐methylpyrroline (**1 a**, 50 mM; Figure 2) as substrate. These experiments confirmed that all ten novel IRED candidates are indeed functional imine reductases.

**Figure 3:**
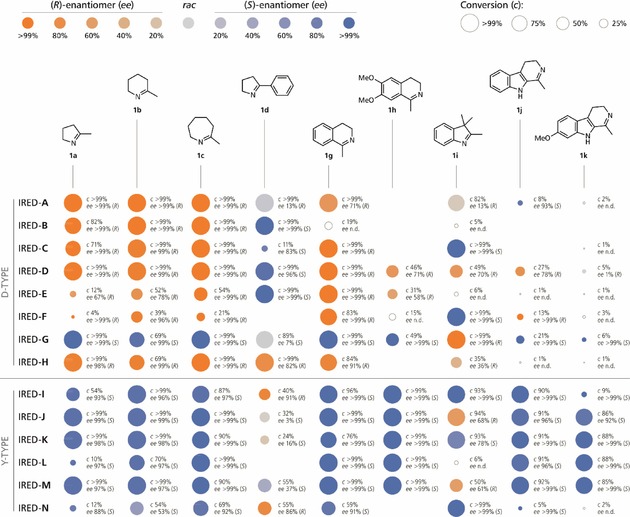
Conversions (*c*) and optical purities (*ee*) observed in the reduction of imines **1 a–d** and **1 g–k** by the investigated D‐type and Y‐type IREDs. *Reaction conditions*: Substrate **1** (10 mM), NADP^+^ (1 mM), IRED (2 mg/mL crude preparation), *Lb*‐ADH (2 mg/mL crude preparation), Tris‐HCl buffer (100 mM, pH 7.5; IREDs **A–E**, **I–M**) or potassium phosphate buffer (100 mM, pH 6.0; IREDs **F–H**, **N**), 2‐PrOH (5 % v/v), 30 °C, 24 h. Empty circles indicate products whose *ee* was not determined, and blank spaces indicate reactions in which no product formation was detected.

Conservation analyses of the *Imine Reductase Engineering Database* have revealed the conservation of Asp187 (IRED‐**B** numbering) in IREDs that reduce **1 a** to the (*R*)‐amine, and of a tyrosine residue in the corresponding position of the (*S*)‐selective enzymes,9aa suggesting that the residue in this position might be used as a predictor of IRED stereoselectivity. More recent studies have shown, however, that functional IREDs of either selectivity can feature other amino acid residues, including those with non‐acidic side‐chains, in the ‘proton donor’ position,9l,9s,9x,9y which demonstrates that the factors governing IRED selectivity are more complex than initially assumed. Chiral‐phase GC analysis of the biotransformations of substrate **1 a** by the 14 IREDs investigated in this study showed that with one exception all enzymes exhibit the expected stereoselectivity (*R* for D‐type, *S* for Y‐type). Only IRED‐**G** (L8EIW6) breaks this pattern: it is a D‐type IRED but forms (*S*)‐2‐methylpyrrolidine from **1 a** in >99 % *ee*. The same ‘inverse’ stereoselectivity is also observed with several other substrates (see Figure 3 below). This finding is interesting in the context of a recent analysis by Fademrecht *et al*., who found that two active‐site residues consistently differ between (*R*)‐ and (*S*)‐selective IREDs: Previously described (*S*)‐selective enzymes invariably have a proline in position 139 and a phenylalanine in position 194 (IRED‐**B** numbering for both positions), while (*R*)‐selective IREDs feature a hydrophobic residue (Val, Thr, Ile) in position 139 and methionine or leucine in position 194.^11^ The partial sequence alignment in Table 1 shows that IRED‐**G** contains the proline and phenylalanine residues typical for (*S*)‐IREDs, indicating that these are better predictors for IRED stereoselectivity than overall sequence homology or the nature of the ‘proton donor’ residue.

### Enzyme Characterisation

After confirming the imine reductase activity of the novel IREDs and determining their stereoselectivity in the reduction of **1 a**, we proceeded with a thorough characterisation of the enzymes with respect to their pH‐activity profile, their temperature stability, their cofactor preference, and their activity and stereoselectivity towards a broader range of substrates. The known IREDs **A**, **B**, **I**, and **K** were included in these experiments for comparison.

Analysis of the pH‐activity profiles revealed two groups of enzymes, one showing highest activity at pH 7.0–7.5 (IREDs **A**–**E**, **I**–**M**), the other preferring slightly acidic pH values (pH 6.0; IREDs **F**–**H**, **N**). The temperature stability varied widely: Some enzymes (e. g., IREDs **A**, **M**) retained significant activity even after incubation at 50 °C for 1 h, while others (e. g., IRED‐**B**) were completely inactivated even by a 1 h incubation at 37 °C. The preferred cofactor for all investigated enzymes is NADPH, but NADH was also accepted by some of the enzymes at appreciable rates. For instance, IRED‐**H** showed 25 % of its maximum activity when NADH was used as cofactor instead of NADPH. Detailed data on pH optimum, thermostability, and cofactor preference are provided in the Supporting Information (Supporting Figures S1–S5).

The specific activities of the IREDs towards substrates **1 a**–**g** were determined by spectrophotometrically following the consumption of NADPH in biotransformations using enzymes purified by Ni^2+^‐NTA affinity chromatography. Table 2 summarises the obtained results. Although activity was detectable for all but two substrate–enzyme combinations, the observed values vary over a wide range: The specific activities of different IREDs towards the same substrate span up to three orders of magnitude (e. g., substrate **1 d**) and the activity of the same enzyme towards the seven imines tested can range from as low as 1.4 mU/mg to >2700 mU/mg (IRED‐**F**, substrates **1 a** and **1 e**). 3,4‐Dihydroisoquinoline (**1 e**) turned out to be a particularly well‐accepted substrate, being reduced at initial rates of >500 mU/mg by 10 out of the 14 IREDs.

**Table 2:** Specific activities of the investigated imine reductases for the reduction of imines **1 a**–**1 g**.

	Specific Activity [mU/mg]						
Enzyme	**1 a**	**1 b**	**1 c**	**1 d**	**1 e**	**1 f**	**1 g**
IRED‐**A**	433.2±17.4	559.9±44.2	1164.0±58.3	222.9±23.8	242.7±13.1	1642.1±84.5	251.3±5.8
IRED‐**B**	10.7±0.6	350.9±12.1	11.1±0.1	21.6±0.8	2.7±0.2	6.8±0.3	<1.0
IRED‐**C**	17.3±1.0	368.2±24.1	192.8±6.1	1.8±0.1	1719.6±59.9	150.3±4.0	644.8±68.7
IRED‐**D**	21.9±0.9	233.8±5.3	90.7±4.4	3.8±0.2	889.0±35.5	123.6±5.3	534.2±12.6
IRED‐**E**	6.8±0.2	76.1±7.3	3.8±0.1	33.3±0.9	155.0±6.7	206.9±6.0	241.8±4.3
IRED‐**F**	1.4±0.3	15.4±1.0	6.9±0.5	0.8±0.0	2703.2±79.4	14.6±1.6	1155.9±48.4
IRED‐**G**	35.3±1.6	324.6±2.6	193.2±3.6	12.7±0.2	844.4±23.5	66.6±2.9	162.1±20.0
IRED‐**H**	359.8±9.8	360.5±19.8	397.3±36.1	1594.0±87.5	1153.8±108.2	810.3±21.4	334.4±24.1
IRED‐**I**	17.8±0.9	222.1±5.5	85.8±3.0	4.4±0.1	1622.6±70.6	1472.6±140.6	77.8±1.9
IRED‐**J**	38.1±1.6	349.5±5.6	75.2±7.0	1.4±0.1	1849.2±58.1	705.3±92.8	2333.7±46.2
IRED‐**K**	25.7±1.5	227.3±30.1	88.8±4.9	1.9±0.0	664.5±48.5	308.4±8.9	11.3±0.6
IRED‐**L**	18.6±1.4	369.7±3.9	132.8±4.1	1.3±0.1	1288.8±65.0	127.3±1.8	5442.8±115.5
IRED‐**M**	18.4±0.8	98.5±4.8	23.8±0.6	2.2±0.1	740.2±15.4	251.8±15.4	1307.0±20.5
IRED‐**N**	6.1±0.5	43.9±2.4	14.7±1.8	<1.0	379.9±1.1	278.4±3.9	43.3±2.3

*Assay conditions*: substrate **1 a–g** (10 mM), NADPH (2 mM), IRED (0.033–3.5 mg/mL purified enzyme), Tris‐HCl buffer (100 mM, pH 7.5; IREDs **A–E**, **I–M**) or potassium phosphate buffer (100 mM, pH 6.0; IREDs **F–H**, **N**), MeOH (5 % v/v), 30 °C.

To obtain information on the stereoselectivity of the investigated IREDs and on their activity towards substrates **1 h**–**k**, which are not amenable to photometric activity screening, we next performed biotransformations of all ten imines **1 a**–**k** (10 mM) employing lyophilised crude cell‐free extracts of IREDs **A**–**N**. For *in situ* regeneration of the NADPH cofactor, we chose the well‐known alcohol dehydrogenase from *Lactobacillus brevis* (*Lb*‐ADH), employing isopropanol (5 % v/v) as cheap and innocuous sacrificial co‐substrate. This approach, which represents a biocatalytic alcohol‐to‐imine hydrogen transfer, is enabled by the excellent chemoselectivity of the involved enzymes: Alcohol dehydrogenases are unable to reduce imines, while imine reductases are generally unreactive towards carbonyl compounds.^13^ As shown in Figure 3, high conversions and excellent optical purities were attained in many of the reactions and the observed stereoselectivities largely follow the expected patterns, with the previously discussed exception of the (*S*)‐selective D‐type IRED‐**G**. Among the general trends that emerge from the data are the poor acceptance of sterically demanding imines **1 h**, **1 j** and **1 k** by D‐type IREDs and the unpredictable and in many cases moderate stereoselectivities observed with substrates **1 d** and **1 i**.

### Process Optimisation

**Figure 4:**
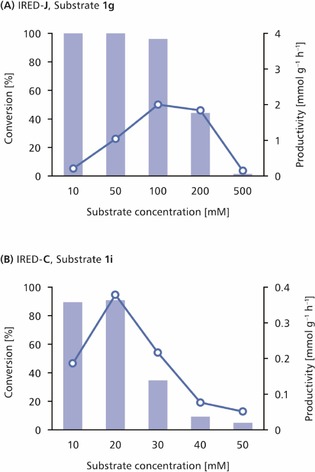
Conversions (bars) and productivities (circles) achieved in the reduction of **(A)** imine **1 g** using IRED‐**J**, and **(B)** imine **1 i** using IRED‐**C**. Note the different scaling of the substrate concentration and productivity axes in **(A)** and **(B)**. *Reaction conditions*: Substrate **1 g** or **1 i** (10–500 mM), NADP^+^ (1 mM), IRED‐**J** or IRED‐**C** (2 mg/mL crude preparation), *Lb*‐ADH (2 mg/mL crude preparation), Tris‐HCl buffer (100 mM, pH 7.5), 2‐PrOH (5 % v/v), 30 °C, 24 h.

Many of the biotransformations reported in Figure 3 reached completion already within 2 h (Supplementary Tables S1–S4, Supporting Information), which prompted us to challenge the enzymes with substantially higher substrate concentrations while keeping all other reaction conditions unaltered.^14^ Gratifyingly, many of the reactions conducted at elevated substrate loading proceeded smoothly to high conversions, whereby the highest productivities were often observed at 100–200 mM concentration of the imine (for an exemplary data set, see Figure 4; for complete data, see Supplementary Figures S6–S9 in the Supporting Information). These results are not surprising in the light of previous Michaelis−Menten kinetic studies on IREDs, which found *K*_M_ values of typical imine substrates to be in the millimolar range.9t,9v,9ab–9ad,9ah These enzymes therefore reach maximum activity only in the presence of high‐millimolar concentrations of imines. In some cases, however, elevated substrate concentrations were not well tolerated. For instance, the conversion of indoleine **1 i** by IREDs **C**, **G**, and **I** dropped from >90 % to below 5 % upon raising the substrate concentration from 10 mM to 50 mM (Figure 4, B; Supplementary Figure S9 in the Supporting Information).

Out of the biotransformations that worked well at 100 mM substrate concentration, two were chosen for reactions at preparative scale (5 mmol): Imine **1 b** was reduced by IRED‐**J** to (*S*)‐2‐methylpiperidine (**2 b**), which was isolated as the corresponding acetamide derivative in 74 % yield and >99 % *ee*. The reduction of 1‐methyl‐3,4‐dihydroisoquinoline (**1 g**) by IRED‐**D** afforded (*R*)‐**2 g** in 98 % *ee* and 91 % isolated yield.

### Co‐expression of IREDs and *Lb*‐ADH

**Figure 5:**
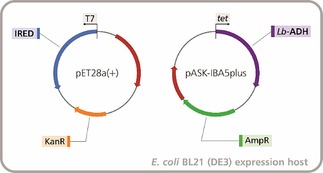
Two‐plasmid system for co‐expression of IREDs and *Lb*‐ADH in a single *E. coli* BL21 (DE3) host. Promoters, resistance genes and target genes are labelled. Red arrows (unlabelled) represent repressor genes (lacI in pET28a(+), tetR in pASK‐IBA5plus).

Although the biotransformations using lyophilised cell‐free extracts of IREDs and *Lb*‐ADH gave excellent results, we anticipated that a whole‐cell biocatalyst would be even better applicable to preparative‐scale reactions. In particular, co‐expression of IREDs and *Lb*‐ADH in a single host would provide a convenient ‘all‐in‐one’ biocatalyst that could benefit from intracellular cofactor cycling, rendering the external addition of NADP^+^ unnecessary. Because the plasmids used for expression of the imine reductases and of *Lb*‐ADH are complementary with respect to both antibiotic resistance and induction conditions, implementation of a two‐plasmid co‐expression system was straightforward (Figure 5).

**Figure 6:**
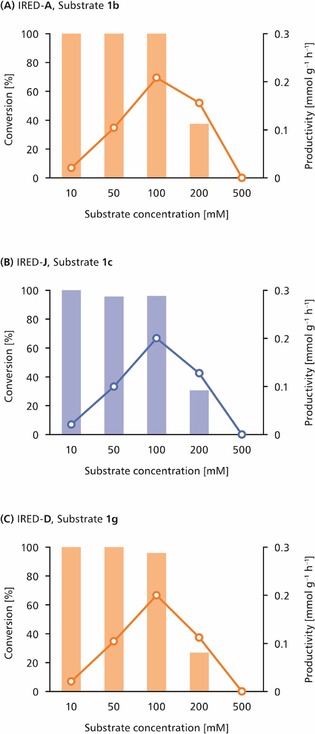
Conversions (bars) and productivities (circles) achieved in the reduction of imines **1 b**, **1 c**, and **1 g** using a whole‐cell biocatalyst co‐expressing *Lb*‐ADH and IRED **A**, **J**, and **D**, respectively. Data from (*R*)‐selective reductions are shown in orange, those from (*S*)‐selective reductions are shown in blue. *Reaction conditions*: Substrate **1** (10–500 mM), *E. coli* BL21 (DE3) co‐expressing IRED and *Lb*‐ADH (20 mg/mL lyophilised cells), Tris‐HCl buffer (100 mM, pH 7.5), 2‐PrOH (5 % v/v), 30 °C, 24 h.

The ‘designer cells’ co‐expressing *Lb*‐ADH and IREDs worked well in the absence of additional NADP^+^, even after prolonged storage of the lyophilised preparations at 4 °C (data not shown). Biotransformations carried out with 20 mg/mL of lyophilised cells gave results comparable to those obtained with 2 mg/mL of lyophilised cell‐free extract, and elevated substrate concentrations were also tolerated similarly well (see Figure 6 for three examples; for complete data see Supplementary Figures S10–S16 in the Supporting Information). No significant differences were observed in the enantioselectivities of the reductions catalysed by the ‘designer cells’ compared to those catalysed by the cell‐free enzyme preparations.

The preparative applicability of the IRED/*Lb*‐ADH ‘designer cells’ was demonstrated by two reductions at gram scale (10 mmol substrate at 100 mM concentration): The biotransformation of imine **1 c** by cells co‐expressing *Lb*‐ADH and IRED‐**D** afforded, after *in situ* derivatisation with acetic anhydride, the acetamide derivative of amine (*R*)‐**2 c** in 65 % isolated yield (1.01 g) and >99 % *ee*. Using an *Lb*‐ADH/IRED‐**J** ‘designer cell’, (*S*)‐1‐methyl‐1,2,3,4‐tetrahydroisoquinoline (**2 g**) was obtained from the corresponding imine **1 g** in 94 % yield (1.38 g) and in optically pure form (*ee* >99 %).

## Conclusions

To summarise, we report the expression and characterisation of eight novel imine reductases along with six literature‐known enzymes, thereby contributing to the further expansion of the growing IRED enzyme ‘toolbox’. Basic characterisation of the new enzymes revealed two distinct groups with respect to the pH optimum and wide variations in temperature stability. The specific activities of the IREDs towards the investigated imines **1 a**–**g** ranged from <1 mU/mg to >5 U/mg. In terms of substrate scope, group‐specific trends were observed for the ‘D‐type’ and ‘Y‐type’ branches of the phylogenetic tree, as the D‐type enzymes gave particularly poor results with sterically demanding imines. The stereoselectivity of the enzymes, on the other hand, was most reliably predicted not by their affiliation to either of the two phylogenetic branches but rather by the presence of key residues identified in a recent bioinformatics analysis by Pleiss and co‐workers.^11^

Cofactor regeneration *via* formal alcohol‐to‐imine hydrogen transfer employing alcohol dehydrogenase from *Lactobacillus brevis* as auxiliary enzyme was successfully implemented and allowed the imine reductions to proceed efficiently at elevated substrate concentrations (100 mM) and on preparative scale. A further simplification of the reaction system was achieved by using an IRED/ADH ‘designer cell’ as biocatalyst, which showed satisfactory imine‐reducing activity also in the absence of additional NADP^+^. Studies aimed at applying the IREDs described herein to the preparation of more complex amines are currently underway in our laboratory, and their results will be reported in due course.

## **Experimental**

### General Methods and Materials

^1^H‐ and ^13^C‐NMR spectra were recorded using a 300 MHz instrument. Chemical shifts are given in parts per million (ppm) relative to TMS (*δ*=0 ppm) and coupling constants (*J*) are reported in Hertz (Hz). Melting points were determined in open capillary tubes and are uncorrected. Thin layer chromatography was carried out on silica gel 60 F_254_ plates and compounds were visualised either by dipping into cerium ammonium molybdate (CAM) reagent [100 g/L (NH_4_)_6_Mo_7_O_24_ ⋅ 4 H_2_O, 4 g/L Ce(SO_4_)_2_ ⋅ 4 H_2_O, in 10 % aq. H_2_SO_4_], by dipping into basic permanganate reagent (10 g/L KMnO_4_, 50 g/L Na_2_CO_3_, 0.85 g/L NaOH, in H_2_O), or by UV. Unit resolution GC‐MS analyses were performed using electron impact (EI) ionisation at 70 eV and quadrupole mass selection. Optical rotation values [α]_D_^20^ were measured at 589 nm (Na D‐line) and 20 °C using a cuvette of 1 dm path length.

Unless otherwise noted, reagents and organic solvents were obtained from commercial suppliers in reagent grade quality and used without further purification. Diethyl ether and acetonitrile used for anhydrous reactions were dried over molecular sieves (3 Å) for at least 48 hours. THF used for anhydrous reactions was distilled from potassium/benzophenone directly before use. For anhydrous reactions, flasks were oven‐dried and flushed with dry argon just before use. Standard syringe techniques were applied to transfer dry solvents and reagents in an inert atmosphere of dry argon.

Imines **1 a**, **1 e**, **1 g**, **1 h**, **1 i**, and **1 k** as well as amines **2 a**, **2 b**, and **2 e** were obtained from commercial suppliers and used as received. Synthetic procedures and full characterisation data for all other substrates and products are provided in the Supporting Information.

Alcohol dehydrogenase from *Lactobacillus brevis* and the imine reductases used in this study were heterologously expressed in *E. coli* as described in the Supporting Information.

The analytical methods used for determination of conversion and enantiomeric excess are described in the Supporting Information.

### *In‐silico* Enzyme Discovery

The full‐length protein sequences of IREDs **A**, **B**, **I**, and **K** (UniProt accession codes M4ZRJ3, Q1EQE0, M4ZS15, D2AWI4) were used as templates for protein BLAST searches^15^ in the UniProtKB database (http://www.uniprot.org/blast/) and the generated hit sets were limited to homologues with E‐values of ≤10^−50^, giving a total of 1065 candidate sequences (274 from IRED‐**A**, 319 from IRED‐**B**, 245 from IRED‐**I**, 227 from IRED‐**K**). These sequences were manually checked for the presence of the following features: (1) a full‐length Rossmann‐fold domain with a complete GxGxxG consensus sequence, (2) a polar residue (Ser, Thr) in position 111 (IRED‐**B** numbering), (3) an acidic residue (Asp, Glu) in position 187 (IRED‐**B** numbering), along with apolar residues (Ala, Ile, Leu, Met, Phe, Val) in positions 137 and 191 (IRED‐**B** numbering), or a Tyr residue in position 187 (IRED‐**B** numbering). Of the 1065 candidate sequences, 215 contained all three features. These were further narrowed down to 182 by removal of duplicates (identical database entries found by more than one of the four BLAST searches) and of redundant sequences (database entries having a different accession code but identical sequence). A multiple‐sequence alignment of the 182 candidate proteins was generated using the Clustal Omega online tool (http://www.ebi.ac.uk/Tools/msa/clustalo/)^16^ and arranged into a phylogenetic tree by the neighbour‐joining method, also using the Clustal Omega interface. The tree was visualised using the TreeView 1.6.6 application, exported in enhanced metafile (EMF) format, and coloured and labelled using Adobe Illustrator CS5.

### Photometric Determination of Enzyme Activity

*Determination of pH−activity profiles*: A stock solution of NADPH (20 mM; final concentration in the reaction mixture: 0.2 mM) was prepared in potassium phosphate buffer (20 mM, pH 7.0), and a stock solution of the assay substrate (IREDs **A**, **C**, **D**, **G**, **J**, and **K**: **1 a**, IREDs **B**, **H**, and **L**: **1 b**, IREDs **E**, **F**, **I**, and **N**: **1 e**, IRED‐**M**: **1 g**; 200 mM; final concentration in the reaction mixture: 10 mM) was prepared in 2‐propanol. For each photometric assay reaction, the NADPH stock (10 μL) was mixed with enzyme solution (10 μL; protein concentration: 1.3–27.3 mg/mL; final concentration in the reaction mixture: 0.013–0.273 mg/mL), the appropriate buffer solution (930 μL; citrate−phosphate, 100 mM, pH 5.0–6.0; potassium phosphate, 100 mM, pH 6.0–8.0; Tris−HCl, 100 mM, pH 8.0–9.0; glycine−NaOH, 100 mM, pH 9.0–11.0), and the imine stock solution (50 μL) in a cuvette of 1 cm path length. In addition, negative control reactions lacking enzyme were set up. All reactions, including the negative controls, were performed in triplicate. The assay reactions were followed by measuring the absorbance at 370 nm every 2 s over a period of 5 min using a *Thermo Scientific* GeneSys 10 spectrophotometer. Slopes were determined by applying a linear fit to the linear range of the absorbance curve using the built‐in function of the photometer's *Thermo Scientific* VISIONlite 5 software. Slopes were corrected for spontaneous absorbance decrease (rate obtained from the negative control reactions) and the specific IRED activity was calculated using formula (1) given below.

(1) $$A=\frac{\Delta OD}{\epsilon \cdot l\cdot c_{P}}$$

where *A* [U ⋅ mg^−1^] … IRED activity; Δ*OD* [min^−1^] … slope of absorbance decrease; *ϵ* [L ⋅ mmol^−1^ ⋅ cm^−1^] … extinction coefficient of NADPH (2.216 at 370 nm); *l* [cm] … path length of sample (1.0 in this case); *c*_P_ [mg ⋅ mL^−1^] … concentration of enzyme in the reaction mixture

*Determination of thermostability*: The activity assays were carried out as described above for the determination of the pH–activity profiles, using potassium phosphate buffer (20 mM, pH 7.0) for all measurements. The undiluted enzyme solutions were incubated before the activity measurements at the appropriate temperature (25 °C, 30 °C, 37 °C, 40 °C, or 50 °C) on a benchtop thermoshaker for 1 h.

*Determination of specific activities*: Stock solutions of NADPH (10 mM; final concentration in the reaction mixture: 2 mM) and of the enzyme (0.0467–4.67 mg/mL; final concentration in the reaction mixture: 0.035–3.5 mM) were prepared in the appropriate buffer (IREDs **A**–**E**, **I**–**M**: Tris‐HCl, 100 mM, pH 7.5; IREDs **F**–**H**, **N**: potassium phosphate, 100 mM, pH 6.0). Stock solutions of the assay substrates (**1 a**–**1 g**, 200 mM; final concentration in the reaction mixture: 10 mM) were prepared in 2‐propanol. For each photometric assay reaction, the NADPH stock (20 μL) was mixed with substrate stock (5 μL) and enzyme stock solution (75 μL) in a 96‐well microtitre plate. In addition, negative control reactions (lacking either substrate or enzyme) were set up. All reactions, including the negative controls, were performed in triplicate. The assay reactions were followed by measuring the absorbance at 370 nm every 20 s over a period of 1 h using a *Molecular Devices* SpectraMax M2 plate reader. If needed, the enzyme concentration was adjusted so as to obtain at least 20 data points in the linear range of absorbance decrease. Slopes were determined by applying a linear fit to the linear range of the absorbance curve using the built‐in function of the plate reader's *Molecular Devices* Softmax Pro v6.4 software. Slopes were corrected for spontaneous absorbance decrease (rate obtained from the negative control reactions) and the specific IRED activity was calculated using formula (1), whereby the path length for each well was determined using the PathCheck feature of the Softmax Pro software (*via* a cuvette reference containing only buffer and 2‐propanol).

### Biotransformations

*Screening of IRED activity and stereoselectivity*: A stock solution of substrate **1 a**–**k** (200 mM; final concentration in reaction mixture: 10 mM) was prepared in 2‐propanol, and a stock solution of *Lb*‐ADH (2.11 mg/mL; final concentration in reaction mixture: 2.0 mg/mL) and NADP^+^ (1.05 mM; final concentration in reaction mixture: 1.0 mM) was prepared in the appropriate buffer (IREDs **A**–**E**, **I**–**‐M**: Tris‐HCl, 100 mM, pH 7.5; IREDs **F**–**H**, **N**: potassium phosphate, 100 mM, pH 6.0). The lyophilised IRED cell‐free extract (1.0 mg) was weighed into a microcentrifuge tube (2 mL) and the ADH/NADP^+^ stock (475 μL) as well as the substrate stock (25 μL) were added. The samples were incubated at 30 °C and 120 rpm in a shaking incubator for the appropriate time (2 h, 24 h). The biotransformations were then quenched by addition of sat. aq. Na_2_CO_3_ solution (200 μL) and the resulting solutions were extracted with ethyl acetate (2×500 μL; containing 10 mM *n*‐dodecane as internal standard). The combined extracts were dried over MgSO_4_, centrifuged (13,000 rpm, 1 min), and the supernatant was transferred to a glass vial for GC and/or HPLC analysis of conversion and enantiomeric excess of the product.

*Biotransformations at elevated substrate concentrations using isolated enzymes*: A stock solution of IRED (2.11 mg/mL; final concentration in reaction mixture: 2.0 mg/mL), *Lb*‐ADH (2.11 mg/mL; final concentration in reaction mixture: 2.0 mg/mL), and NADP^+^ (1.05 mM; final concentration in reaction mixture: 1.0 mM) was prepared in the appropriate buffer (IREDs **A**–**E**, **I**–**M**: Tris‐HCl, 100 mM, pH 7.5; IREDs **F**–**H**, **N**: potassium phosphate, 100 mM, pH 6.0). The substrate **1** (5–250 μmol; final concentration in reaction mixture: 10–500 mM) was weighed into a microcentrifuge tube (2 mL) and dissolved in 2‐propanol (25 μL). The enzyme/cofactor stock solution (475 μL) was added and the samples were incubated at 30 °C and 120 rpm in a shaking incubator for 24 h. The biotransformations were then quenched by addition of sat. aq. Na_2_CO_3_ solution (200 μL) and the resulting solutions were extracted with ethyl acetate (2×500 μL; containing 10 mM *n*‐dodecane as internal standard). The combined extracts were dried over MgSO_4_, centrifuged (13,000 rpm, 1 min), and the supernatant was transferred to a glass vial for GC and/or HPLC analysis of conversion and enantiomeric excess of the product. Extracts from reactions containing ≥200 mM of substrate were diluted 5‐fold prior to analysis.

*Preparative‐scale imine reductions using isolated enzymes*:

**(*S*)‐1‐(2‐Methylpiperidin‐1‐yl)ethanone**. In an Erlenmeyer flask (100 mL) with a glass joint, lyophilised, crude IRED‐**J** (100 mg; final concentration in reaction mixture: 2.0 mg/mL), lyophilised, crude *Lb*‐ADH (100 mg; final concentration in reaction mixture: 2.0 mg/mL), and NADP^+^ (40 mg; final concentration in reaction mixture: 1.0 mM) were dissolved in Tris‐HCl buffer (47.5 mL; 100 mM, pH 7.5). Imine **1 b** (486 mg, 5 mmol; final concentration in reaction mixture: 100 mM) was dissolved in 2‐propanol (2.5 mL; final concentration in reaction mixture: 5 % v/v) and added to the biocatalyst solution. The Erlenmeyer flask was closed with a rubber septum and incubated at 30 °C and 120 rpm for 24 h, at which time TLC (silica gel 60, MTBE:MeOH:NH_4_OH=90 : 9 : 1, basic permanganate staining) indicated completion of the biotransformation. The reaction mixture was transferred to a round‐bottom flask (250 mL) and saturated aqueous Na_2_CO_3_ solution (20 mL) as well as ethyl acetate (40 mL) were added. To the resulting biphasic mixture, a solution of acetic anhydride (1.54 g, 15 mmol, 3 eq.) in ethyl acetate (20 mL) was added dropwise over 15 min. The reaction mixture was stirred at room temperature overnight (16 h), at which time TLC (silica gel 60, MTBE:MeOH:NH_4_OH=90 : 9 : 1, basic permanganate staining) indicated completion of the derivatisation reaction. The phases were separated, the aqueous phase was saturated with NaCl and extracted with ethyl acetate (3×50 mL), and the combined organic phases were dried over Na_2_SO_4_, filtered and concentrated under reduced pressure to give 846 mg of a yellow liquid. Column chromatography (silica gel 60, EtOAc) afforded the title compound (520 mg, 74 %) as a pale‐yellowish liquid. TLC (silica gel 60, MTBE:MeOH:NH_4_OH=90 : 9 : 1): *R*_f_=0.83. GC‐MS (EI, 70 eV): *m*/*z*=141 (M^+^, 30), 126 (M^+^–CH_3_, 25), 84 (100), 70 (9), 56 (16), 43 (19). *ee* >99 % (GC). [α]_D_^20^=+62.7 (*c* 1.18, CHCl_3_). NMR analysis revealed that the product is a mixture of amide rotamers (ratio *trans*/*cis*=1.04 : 1), to which the individual NMR signals were assigned based on peak intensities as well as the DEPT, COSY, and HSQC spectra. ***trans*** **‐(*S*)‐1‐(2‐Methylpiperidin‐1‐yl)ethanone**: ^1^H‐NMR (300 MHz, CDCl_3_): *δ* [ppm]=1.13 (3H, d, *J*=7.0 Hz, N‐CH‐CH_3_), 1.32–1.70 (6H, m, 3×CH_2_), 2.06 (3H, s, COCH_3_), 3.15 (1H, td, *J*=13.3 Hz, 2.8 Hz, N‐CH_2_), 3.57 (1H, br d, *J*=13.2 Hz, N‐CH_2_), 4.07–4.13 (1H, m, N‐CH). ^13^C‐NMR (75 MHz, CDCl_3_): *δ* [ppm]=15.5 (CH_3_), 18.7 (CH_2_), 22.0 (CH_3_), 26.2 (CH_2_), 29.8 (CH_2_), 41.6 (CH_2_), 49.1 (CH), 168.9 (C=O). ***cis*** **‐(*S*)‐1‐(2‐Methylpiperidin‐1‐yl)ethanone**: ^1^H‐NMR (300 MHz, CDCl_3_): *δ* [ppm]=1.23 (3H, d, *J*=7.0 Hz, N‐CH‐CH_3_), 1.32–1.70 (6H, m, 3 × CH_2_), 2.09 (3H, s, COCH_3_), 2.63 (1H, td, *J*=13.5 Hz, 2.9 Hz, N‐CH_2_), 4.49 (1H, dd, *J*=13.0 Hz, 2.6 Hz, N‐CH_2_), 4.86–4.93 (1H, m, N‐CH). ^13^C‐NMR (75 MHz, CDCl_3_): *δ* [ppm]=16.5 (CH_3_), 18.7 (CH_2_), 21.4 (CH_3_), 25.4 (CH_2_), 30.7 (CH_2_), 36.1 (CH_2_), 43.4 (CH), 168.9 (C=O). The characterisation data are in agreement with literature values.^17^

**(*R*)‐1‐Methyl‐1,2,3,4‐tetrahydroisoquinoline (*R*)‐2 g**. In an Erlenmeyer flask (100 mL) with a glass joint, lyophilised, crude IRED‐**D** (100 mg; final concentration in reaction mixture: 2.0 mg/mL), lyophilised, crude *Lb*‐ADH (100 mg; final concentration in reaction mixture: 2.0 mg/mL), and NADP^+^ (40 mg; final concentration in reaction mixture: 1.0 mM) were dissolved in Tris‐HCl buffer (47.5 mL; 100 mM, pH 7.5). Imine **1 g**⋅HCl⋅H_2_O (990 mg, 5 mmol; final concentration in reaction mixture: 100 mM) was suspended in 2‐propanol (2.5 mL; final concentration in reaction mixture: 5 % v/v) and added to the biocatalyst solution. The Erlenmeyer flask was closed with a rubber septum and incubated at 30 °C and 120 rpm for 24 h, at which time TLC (silica gel 60, MTBE : MeOH : NH_4_OH=90 : 9 : 1, basic permanganate staining) indicated completion of the biotransformation. The reaction mixture was transferred to plastic centrifuge tubes (2×50 mL) and treated with saturated aqueous Na_2_CO_3_ solution (10 mL each). The product was extracted into ethyl acetate (3×10 mL each; phase separation accelerated by centrifugation at 4,000 rpm) and the combined organic phases were dried over Na_2_SO_4_, filtered and concentrated under reduced pressure to give 771 mg of a slightly yellowish liquid. Column chromatography (silica gel 60, MTBE:MeOH:NH_4_OH=85 : 14 : 1) afforded (*R*)‐**2 g** (673 mg, 91 %) as a pale‐yellowish liquid. TLC (silica gel 60, MTBE:MeOH:NH_4_OH=90 : 9 : 1): *R*_f_=0.26. *ee*=98 % (HPLC). [α]_D_^20^=+81.7 (*c* 1.25, CHCl_3_). ^1^H‐NMR (300 MHz, CDCl_3_): *δ* [ppm]=1.49 (3H, d, *J*=6.7 Hz, CH_3_), 1.79 (1H, s, NH), 2.76 (1H, dt, *J*=16.3 Hz, 4.7 Hz, Ar‐CH_2_), 2.85–2.95 (1H, m, Ar‐CH_2_), 3.05 (1H, ddd, *J*=12.4 Hz, 8.6 Hz, 4.7 Hz, N‐CH_2_), 3.29 (1H, dt, *J*=12.4 Hz, 5.0 Hz, N‐CH_2_), 4.13 (1H, q, *J*=6.7 Hz, N‐CH), 7.08–7.21 (4H, m, Ar−H). ^13^C‐NMR (75 MHz, CDCl_3_): *δ* [ppm]=22.7, 30.1, 41.8, 51.6, 125.9, 125.9, 125.9, 129.2, 134.8, 140.5. GC‐MS (EI, 70 eV): *m*/*z*=147 (M^+^, 2), 146 (M^+^−H, 11), 132 (100), 117 (20). The characterisation data are in agreement with literature values.^18^

*Biotransformations using E. coli ‘designer cells’ co‐expressing IREDs and Lb‐ADH*: Lyophilised *E. coli* ‘designer cells’ co‐expressing the appropriate IRED and *Lb*‐ADH (21.1 mg/mL; final concentration in reaction mixture: 20 mg/mL) were resuspended in the appropriate buffer (IREDs **A**–**E**, **I**–**M**: Tris‐HCl, 100 mM, pH 7.5; IREDs **F**–**H**, **N**: potassium phosphate, 100 mM, pH 6.0). The substrate **1** (5–250 μmol; final concentration in reaction mixture: 10–500 mM) was weighed into a microcentrifuge tube (2 mL) and dissolved in 2‐propanol (25 μL). The cell suspension (475 μL) was added and the samples were incubated at 30 °C and 120 rpm in a shaking incubator for 24 h. The biotransformations were then quenched by addition of sat. aq. Na_2_CO_3_ solution (200 μL) and the resulting suspensions were extracted with ethyl acetate (2×500 μL; containing 10 mM *n*‐dodecane as internal standard). The combined extracts were dried over MgSO_4_, centrifuged (13,000 rpm, 1 min), and the supernatant was transferred to a glass vial for GC and/or HPLC analysis of conversion and enantiomeric excess of the product. Extracts from reactions containing ≥200 mM of substrate were diluted 5‐fold prior to analysis.

*Preparative‐scale imine reductions using E. coli ‘designer cells’ co‐expressing IREDs and Lb‐ADH*:

**(*R*)‐1‐(2‐Methylazepan‐1‐yl)ethanone**. In an Erlenmeyer flask (250 mL) with a screw cap, lyophilised *E. coli* ‘designer cells’ co‐expressing the IRED‐**D** and *Lb*‐ADH (2.0 g; final concentration in reaction mixture: 20 g/L) were resuspended in Tris‐HCl buffer (100 mL; 100 mM, pH 7.5). Imine **1 c** (1.11 g, 10 mmol; final concentration in reaction mixture: 100 mM) was dissolved in 2‐propanol (5.0 mL; final concentration in reaction mixture: 5 % v/v) and added to the cell suspension. The Erlenmeyer flask was closed with a screw cap and incubated at 30 °C and 120 rpm for 24 h, at which time TLC (silica gel 60, MTBE : MeOH : NH_4_OH−90 : 9 : 1, basic permanganate staining) indicated completion of the biotransformation. The reaction mixture was transferred to plastic centrifuge tubes (2×50 mL) and centrifuged (4,000 rpm, 35 min) to pellet the cells. The supernatant was transferred to a round‐bottom flask (500 mL) and saturated aqueous Na_2_CO_3_ solution (40 mL) as well as ethyl acetate (80 mL) were added. To the resulting biphasic mixture, a solution of acetic anhydride (3.06 g, 30 mmol, 3 eq.) in ethyl acetate (20 mL) was added dropwise over 15 min. The reaction mixture was stirred at room temperature overnight (16 h), at which time TLC (silica gel 60, MTBE : MeOH : NH_4_OH=90 : 9 : 1, basic permanganate staining) indicated completion of the derivatisation reaction. The aqueous phase was saturated with NaCl and the entire mixture was filtered through a pad (2 cm) of Celite in a glass frit (Ø6 cm) to break the resulting emulsion. The phases were separated, the aqueous phase was extracted with ethyl acetate (4×75 mL), and the combined organic phases were dried over Na_2_SO_4_, filtered and concentrated under reduced pressure to give 1.33 g of a yellow liquid. Column chromatography (silica gel 60, EtOAc) afforded the title compound (1.01 g, 65 %) as a pale‐yellowish liquid. TLC (silica gel 60, MTBE : MeOH : NH_4_OH=90 : 9 : 1): *R*_f_=0.88. GC‐MS (EI, 70 eV): *m*/*z*=155 (M^+^, 41), 140 (M^+^−CH_3_, 30), 126 (17), 112 (38), 98 (100), 84 (15), 70 (37), 56 (19), 43 (27). *ee* >99 % (GC). [α]_D_^20^=−127.5 (*c* 1.12, CHCl_3_). NMR analysis revealed that the product is a mixture of amide rotamers (ratio *trans*/*cis*=1.07 : 1), to which the individual NMR signals were assigned based on peak intensities as well as the DEPT, COSY, and HSQC spectra. ***trans*** **‐(*R*)‐1‐(2‐Methylazepan‐1‐yl)ethanone**: ^1^H‐NMR (300 MHz, CDCl_3_): *δ* [ppm]=1.03 (3H, d, *J*=6.4 Hz, N‐CH‐CH_3_), 1.15–1.59 (4H, m, 2×CH_2_), 1.66–1.86 (3H, m, CH_2_), 1.96–2.09 (1H, m, CH_2_), 2.10 (3H, s, COCH_3_), 3.01 (1H, ddd, *J*=15.5 Hz, 12.0 Hz, 1.2 Hz, N‐CH_2_), 3.43–3.51 (1H, m, N‐CH_2_), 4.48 (1H, dp, *J*=12.6 Hz, 6.4 Hz, N‐CH). ^13^C‐NMR (75 MHz, CDCl_3_): *δ* [ppm]=19.4 (CH_3_), 21.9 (CH_3_), 25.1 (CH_2_), 29.5 (CH_2_), 30.1 (CH_2_), 35.5 (CH_2_), 42.6 (CH_2_), 49.5 (CH), 170.2 (C=O). ***cis*** **‐(*R*)‐1‐(2‐Methylazepan‐1‐yl)ethanone**: ^1^H‐NMR (300 MHz, CDCl_3_): *δ* [ppm]=1.12 (3H, d, *J*=6.4 Hz, N‐CH‐CH_3_), 1.15–1.59 (4H, m, 2 × CH_2_), 1.66–1.86 (3H, m, CH_2_), 1.96–2.09 (1H, m, CH_2_), 2.10 (3H, s, COCH_3_), 2.59 (1H, ddd, *J*=13.5 Hz, 11.9 Hz, 1.5 Hz, N‐CH_2_), 3.75 (1H, dp, *J*=10.6 Hz, 6.4 Hz, N‐CH), 4.04–4.11 (1H, m, N‐CH_2_). ^13^C‐NMR (75 MHz, CDCl_3_): *δ* [ppm]=20.4 (CH_3_), 21.7 (CH_3_), 25.2 (CH_2_), 28.2 (CH_2_), 29.8 (CH_2_), 36.5 (CH_2_), 39.6 (CH_2_), 53.5 (CH), 169.8 (C=O).

**(*S*)‐1‐Methyl‐1,2,3,4‐tetrahydroisoquinoline (*S*)‐2 g**. In an Erlenmeyer flask (250 mL) with a screw cap, lyophilised *E. coli* ‘designer cells’ co‐expressing the IRED‐**J** and *Lb*‐ADH (2.0 g; final concentration in reaction mixture: 20 g/L) were resuspended in Tris‐HCl buffer (100 mL; 100 mM, pH 7.5). Imine **1 g**⋅HCl (1.84 g, 10 mmol; final concentration in reaction mixture: 100 mM) was suspended in 2‐propanol (5.0 mL; final concentration in reaction mixture: 5 % v/v) and added to the cell suspension. The Erlenmeyer flask was closed with a screw cap and incubated at 30 °C and 120 rpm for 24 h, at which time TLC (silica gel 60, MTBE:MeOH:NH_4_OH=90 : 9 : 1, basic permanganate staining) indicated completion of the biotransformation. The reaction mixture was transferred to plastic centrifuge tubes (4×50 mL) and treated with saturated aqueous Na_2_CO_3_ solution (10 mL each). The product was extracted into ethyl acetate (3×10 mL each; phase separation accelerated by centrifugation at 4,000 rpm) and the combined organic phases were dried over Na_2_SO_4_, filtered and concentrated under reduced pressure to give 1.52 g of a slightly yellowish liquid. Column chromatography (silica gel 60, MTBE:MeOH:NH_4_OH=85 : 14 : 1) afforded (*S*)‐**2 g** (1.38 g, 94 %) as a pale‐yellowish liquid. TLC (silica gel 60, MTBE : MeOH : NH_4_OH=90 : 9 : 1): *R*_f_=0.26. *ee* >99 % (HPLC). [α]_D_^20^=−82.5 (*c* 1.10, CHCl_3_). ^1^H‐NMR (300 MHz, CDCl_3_): *δ* [ppm]=1.49 (3H, d, *J*=6.7 Hz, CH_3_), 2.02 (1H, s, NH), 2.76 (1H, dt, *J*=16.3 Hz, 4.7 Hz, Ar‐CH_2_), 2.85–2.96 (1H, m, Ar‐CH_2_), 3.05 (1H, ddd, *J*=12.4 Hz, 8.6 Hz, 4.7 Hz, N‐CH_2_), 3.29 (1H, dt, *J*=12.4 Hz, 5.1 Hz, N‐CH_2_), 4.14 (1H, q, *J*=6.7 Hz, N‐CH), 7.08–7.21 (4H, m, Ar−H). ^13^C‐NMR (75 MHz, CDCl_3_): *δ* [ppm]=22.7, 30.0, 41.8, 51.6, 125.9 (×2), 126.0, 129.2, 134.7, 140.4. GC‐MS (EI, 70 eV): *m*/*z*=147 (M^+^, 2), 146 (M^+^–H, 11), 132 (100), 117 (19). The characterisation data are in agreement with literature values.^18^

## Conflict of interest

The authors declare no conflict of interest.

## Supporting information

As a service to our authors and readers, this journal provides supporting information supplied by the authors. Such materials are peer reviewed and may be re‐organized for online delivery, but are not copy‐edited or typeset. Technical support issues arising from supporting information (other than missing files) should be addressed to the authors.

Supplementary
