# Supplementary material for: Sequence‐Based In‐silico Discovery, Characterisation, and Biocatalytic Application of a Set of Imine Reductases
Source: ChemCatChem. 2018 Jul 17;10(15):3236–46. doi: 10.1002/cctc.201800607 (PMC6120462; doi:10.1002/cctc.201800607)
Supplement: Supplementary file 1 — Supplementary [file CCTC-10-3236-s001.pdf]

Supporting Information

© Copyright Wiley-VCH Verlag GmbH & Co. KGaA, 69451 Weinheim, 2018

Sequence-Based *In-silico* Discovery, Characterisation, and Biocatalytic Application of a Set of Imine Reductases

Stefan Velikogne, Verena Resch, Carina Dertnig, Joerg H. Schrittwieser,* and Wolfgang Kroutil© 2018 The Authors. Published by Wiley-VCH Verlag GmbH & Co. KGaA. This is an open access article under the terms of the Creative Commons Attribution License, which permits use, distribution and reproduction in any medium, provided the original work is properly cited.This publication is part of the Young Researchers Series. More information regarding these excellent researchers can be found on the ChemCatChem homepage

Author Contributions

S.V. Investigation:Lead; Visualization:Supporting; Writing – review & editing:Equal
V.R. Investigation:Supporting; Visualization:Supporting; Writing – review & editing:Equal
C.D. Investigation:Supporting; Writing – review & editing:Equal
J.S. Conceptualization:Lead; Funding acquisition:Supporting; Investigation:Supporting; Methodology:Lead; Project administration:Lead; Supervision:Lead; Visualization:Lead; Writing – original draft:Lead; Writing – review & editing:Equal
W.K. Funding acquisition:Lead; Project administration:Supporting; Writing – review & editing:Equal

Contents:

Supplementary Data S2
- pH–Activity Profiles S2
- Thermostability S4
- Cofactor Preference S7
- Substrate Scope and Stereoselectivity S7
- Process Optimisation S9

Supplementary Methods S16
- Synthesis of Substrates and Reference Compounds S16
- Gene Synthesis and Subcloning S19
- Protein Expression and Purification S20
- Analytical Methods S24

DNA and Protein Sequences S41

NMR and MS Spectra S56

References S76

Supplementary Data

pH–Activity Profiles

The pH–activity profiles of IREDs **A–N** were determined spectrophotometrically using substrates **1a**, **1b**, **1e**, or **1g** as appropriate. The activity values in each figure have been normalised to 100% of maximum activity for clarity.

(A) IRED-**A** (M4ZRJ3)

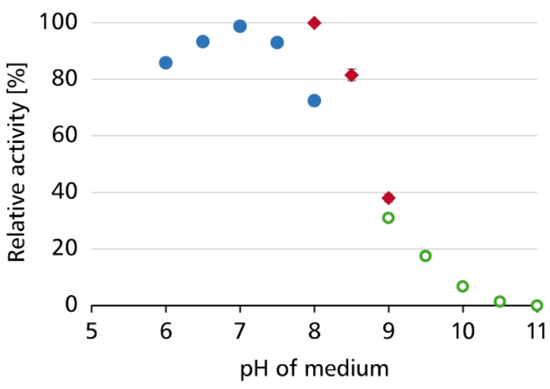

(B) IRED-**B** (Q1EQE0)

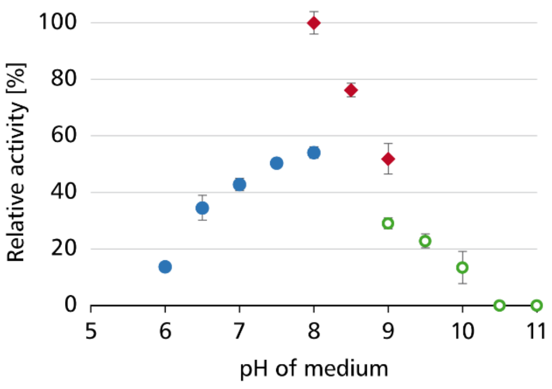

(C) IRED-**C** (W7VJL8)

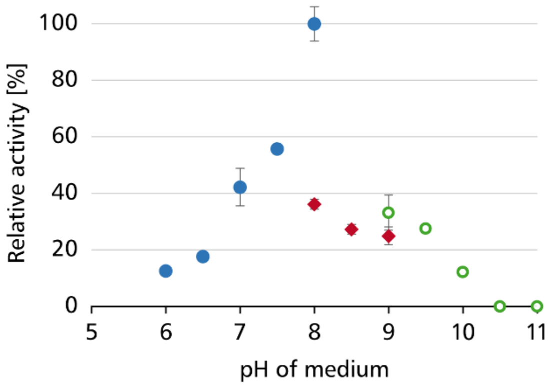

(D) IRED-**D** (V7GV82)

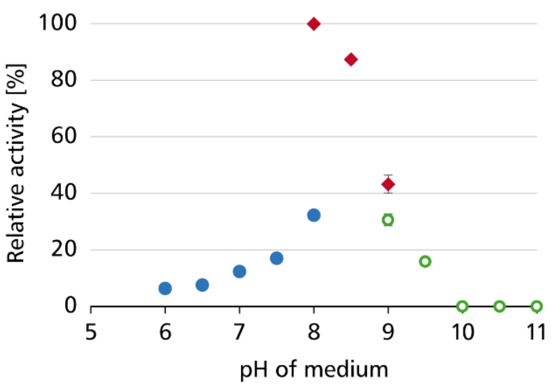

(E) IRED-**E** (J7LAY5)

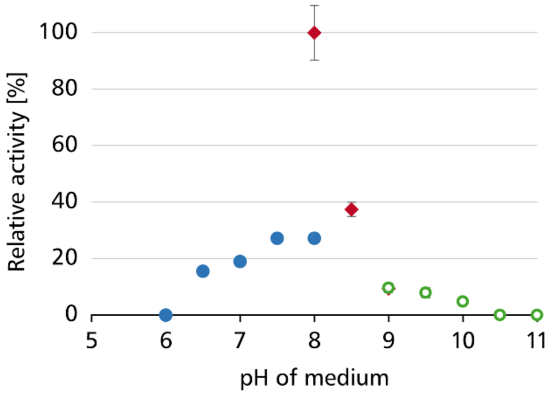

(F) IRED-**F** (V6KA13)

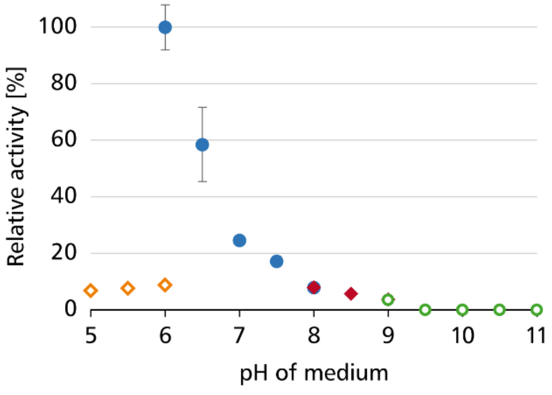

Supplementary Figure S1 (*continued on next page*).

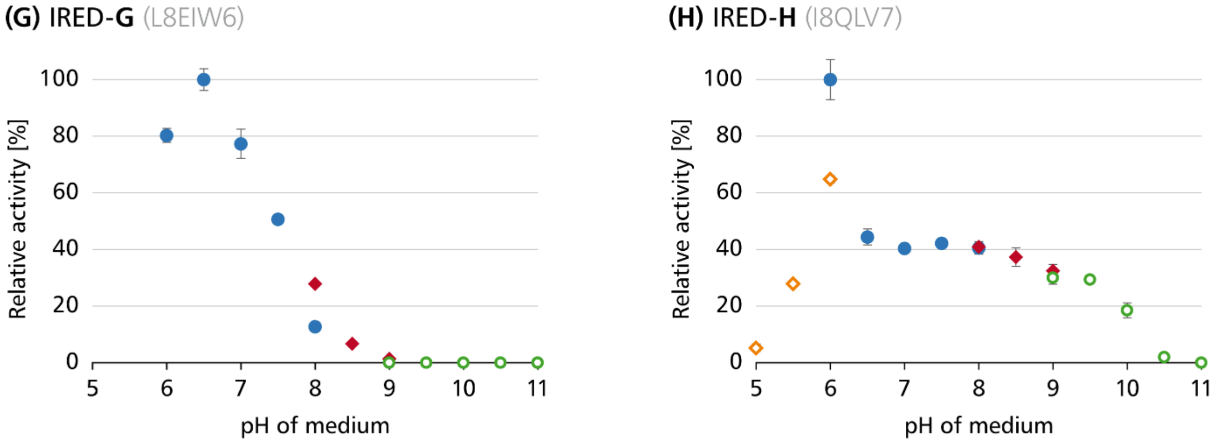

Supplementary Figure S1 (*continued from previous page*). pH–activity profiles of D-type IREDs **A–H**, determined using the following buffer systems: citrate–phosphate (◇), sodium phosphate (●), Tris–HCl (◆), and glycine–NaOH (○). An activity of 100% corresponds to: **(A)** 574.5 mU/mg, substrate **1a**; **(B)** 291.4 mU/mg, substrate **1b**; **(C)** 35.5 mU/mg, substrate **1a**; **(D)** 206.3 mU/mg, substrate **1a**; **(E)** 87.2 mU/mg, substrate **1e**; **(F)** 10.3 mU/mg, substrate **1e**; **(G)** 124.6 mU/mg, substrate **1a**; **(H)** 4.06 U/mg, substrate **1b**. Error bars represent standard deviations of triplicate experiments. *Assay conditions:* Substrate (10 mM), NADPH (0.2 mM), IRED (0.038–0.234 mg/mL), buffer (100 mM, pH 5.0–11.0), 30 °C.

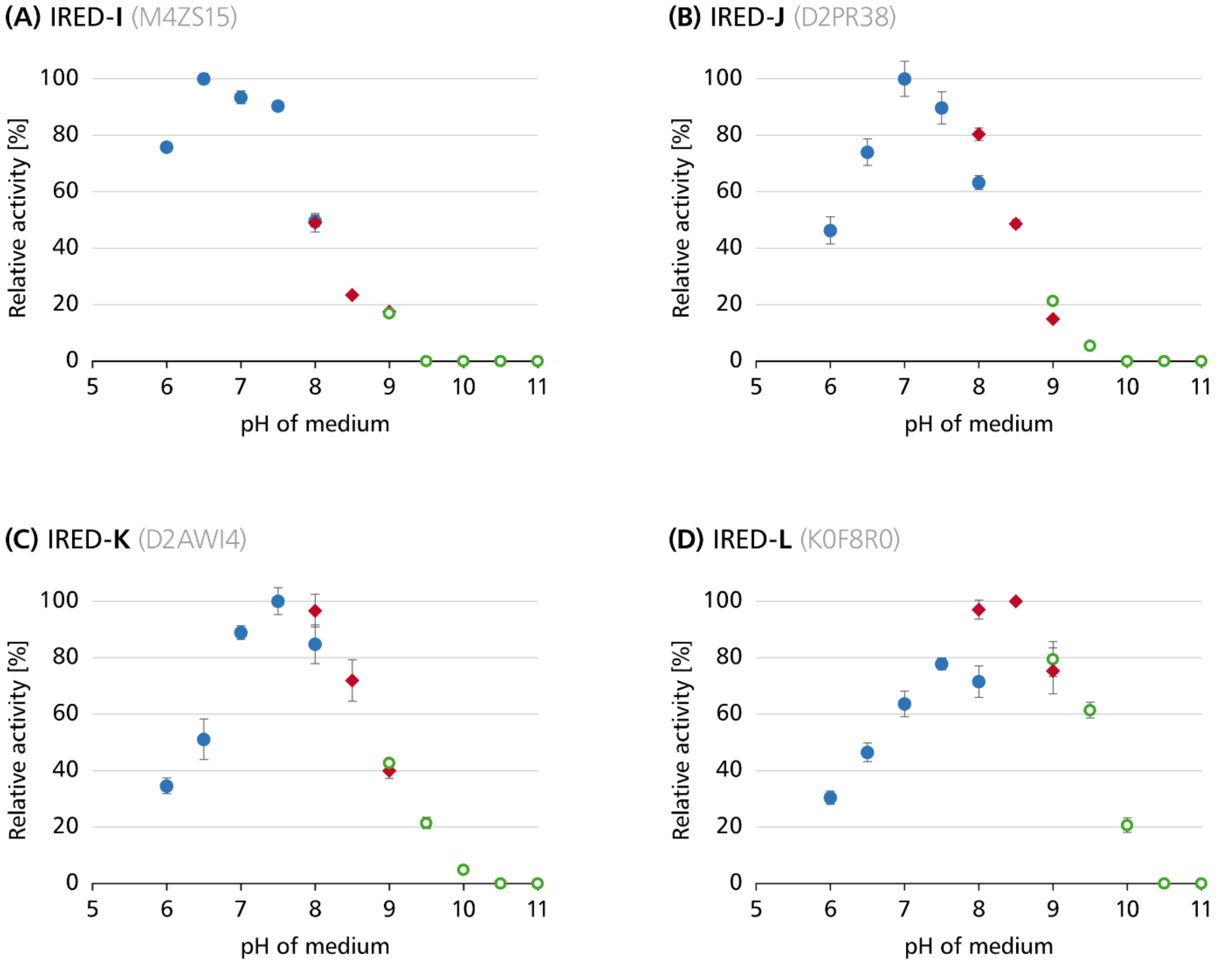

Supplementary Figure S2 (*continued on next page*).

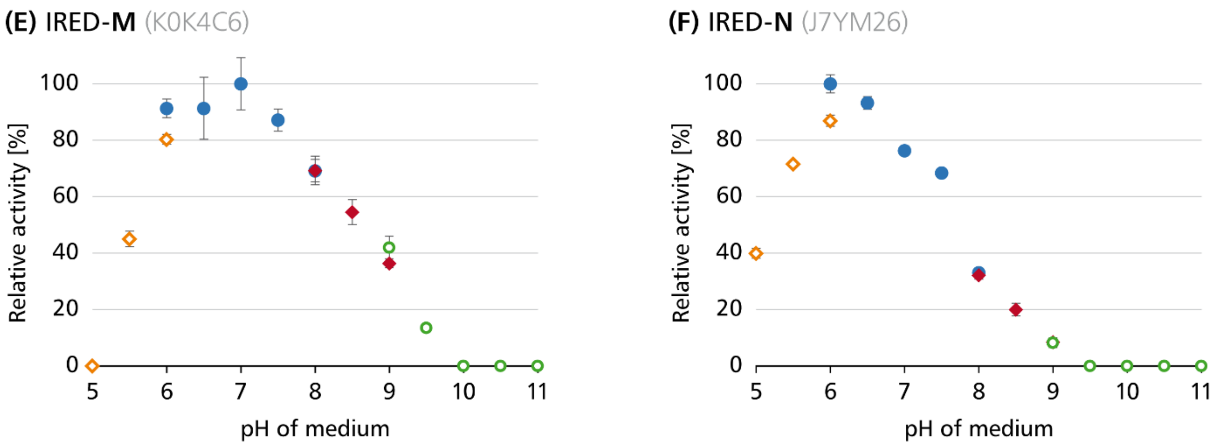

Supplementary Figure S2 (*continued from previous page*). pH–activity profiles of Y-type IREDs **I–N**, determined using the following buffer systems: citrate–phosphate (◇), sodium phosphate (●), Tris–HCl (◆), and glycine–NaOH (○). An activity of 100% corresponds to: **(A)** 1.54 U/mg, substrate **1e**; **(B)** 47.8 mU/mg, substrate **1a**; **(C)** 37.3 mU/mg, substrate **1a**; **(D)** 436.2 mU/mg, substrate **1b**; **(E)** 1.46 U/mg, substrate **1g**; **(F)** 426.4 mU/mg, substrate **1e**. Error bars represent standard deviations of triplicate experiments. *Assay conditions:* Substrate (10 mM), NADPH (0.2 mM), IRED (0.013–0.273 mg/mL), buffer (100 mM, pH 5.0–11.0), 30 °C.

Thermostability

The thermostability of IREDs **A–N** were assessed by spectrophotometric determination of their residual activity in the reduction of substrates **1a**, **1b**, **1e**, or **1g** after incubation at varied temperatures for 1 h. The activity values in each figure have been normalised to 100% of maximum activity for clarity.

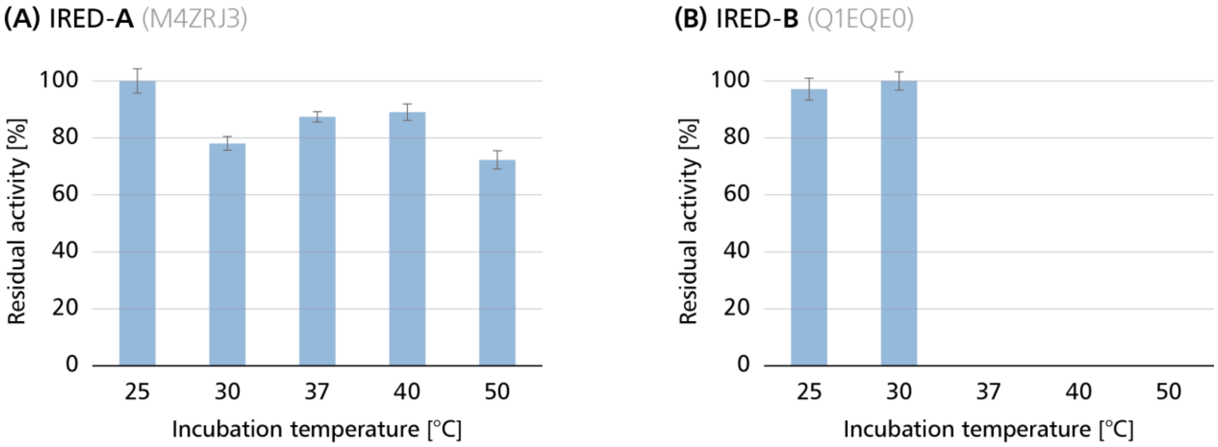

Supplementary Figure S3 (*continued on next page*).

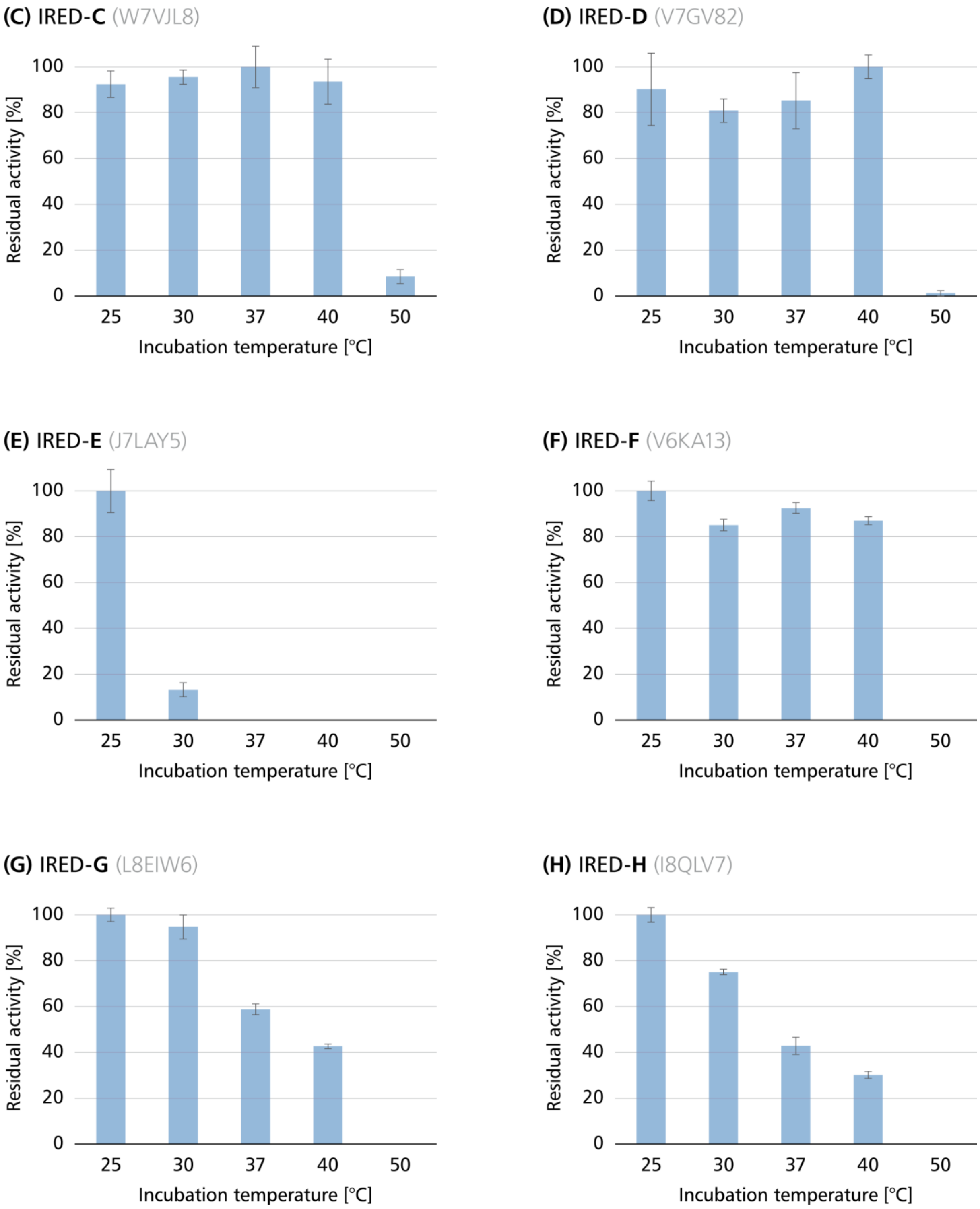

Supplementary Figure S3 (*continued from previous page*). Residual activities of D-type IREDs **A**–**H** after incubation of a concentrated enzyme solution (3.8–23.4 mg/mL) at the indicated temperatures for 1 h. An activity of 100% corresponds to: **(A)** 127.9 mU/mg, substrate **1a**; **(B)** 536.5 mU/mg, substrate **1b**; **(C)** 19.3 mU/mg, substrate **1a**; **(D)** 212.8 mU/mg, substrate **1a**; **(E)** 42.6 mU/mg, substrate **1e**; **(F)** 1.41 U/mg, substrate **1e**; **(G)** 149.9 mU/mg, substrate **1a**; **(H)** 912.8 U/mg, substrate **1b**. Error bars represent standard deviations of triplicate experiments. *Assay conditions:* Substrate (10 mM), NADPH (0.2 mM), IRED (0.038–0.234 mg/mL), potassium phosphate buffer (20 mM, pH 7.0), 30 °C.

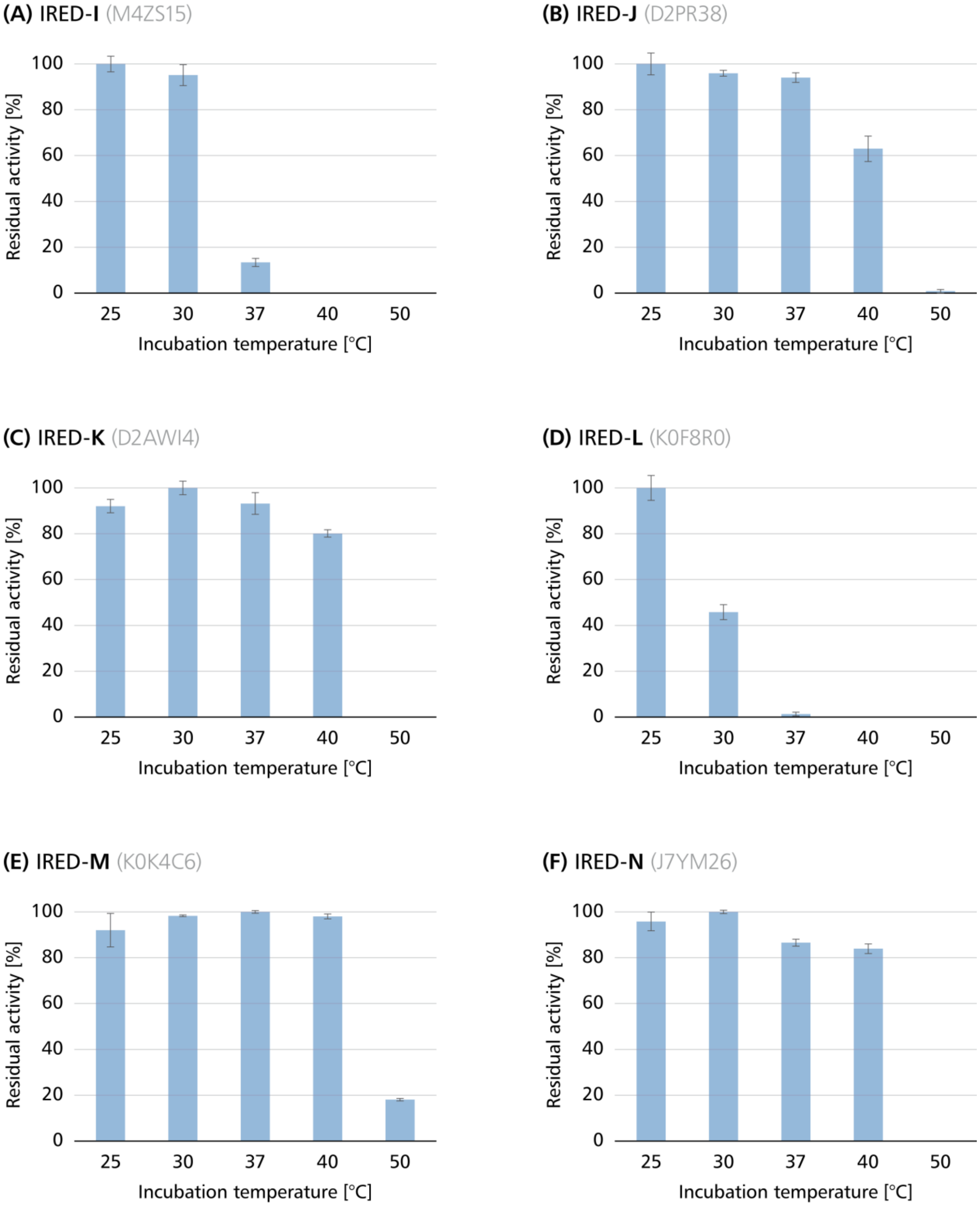

Supplementary Figure S4. Residual activities of Y-type IREDs **I**–**N** after incubation of a concentrated enzyme solution (1.3–27.3 mg/mL) at the indicated temperatures for 1 h. An activity of 100% corresponds to: **(A)** 1.62 U/mg, substrate **1e**; **(B)** 68.7 mU/mg, substrate **1a**; **(C)** 28.2 mU/mg, substrate **1a**; **(D)** 442.8 mU/mg, substrate **1b**; **(E)** 1.46 U/mg, substrate **1g**; **(F)** 283.4 mU/mg, substrate **1e**. Error bars represent standard deviations of triplicate experiments. *Assay conditions:* Substrate (10 mM), NADPH (0.2 mM), IRED (0.013–0.273 mg/mL), potassium phosphate buffer (20 mM, pH 7.0), 30 °C.

Cofactor Preference

The cofactor preference of IREDs **A**, **B**, **D**, **F**, **H**, **I**, and **J** was assessed by spectrophotometric determination of their activity in the reduction of substrates **1b**, **1f**, or **1g** in the presence of NADPH and NADH (2 mM of either cofactor).

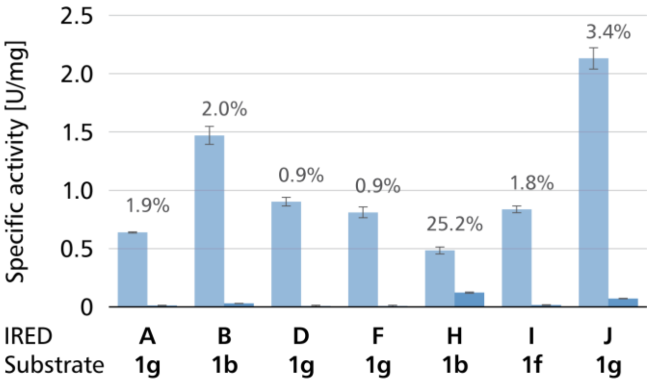

Supplementary Figure S5. Specific activities of various IREDs with NADPH (light blue bars) and NADH (dark blue bars) as cofactor. Error bars represent standard deviations of triplicate experiments. The percentage values shown above the bars are the relative activities of the enzymes with NADH compared to NADPH.

Substrate Scope and Stereoselectivity

Complete substrate scope and selectivity data (conversions after 2 h and after 24 h, and enantiomeric excess after 24 h) are shown in the following tables.

Substrate	1a			1b			1c		
	conv. [%]		*ee* [%]	*conv.* [%]		*ee* [%]	*conv.* [%]		*ee* [%]
	2 h	24 h		2 h	24 h		2 h	24 h	
IRED-**A**	>99	>99	>99 (*R*)	69	>99	>99 (*R*)	90	>99	>99 (*R*)
IRED-**B**	46	82	>99 (*R*)	68	>99	>99 (*R*)	83	>99	>99 (*R*)
IRED-**C**	12	71	>99 (*R*)	63	>99	99 (*R*)	>99	>99	>99 (*R*)
IRED-**D**	68	>99	>99 (*R*)	67	>99	99 (*R*)	>99	>99	>99 (*R*)
IRED-**E**	8	12	67 (*R*)	52	52	78 (*R*)	18	54	>99 (*R*)
IRED-**F**	–	4	>99 (*R*)	–	39	96 (*R*)	–	21	>99 (*R*)
IRED-**G**	57	>99	>99 (*S*)	55	69	99 (*S*)	88	>99	>99 (*S*)
IRED-**H**	63	>99	98 (*R*)	54	69	99 (*R*)	83	>99	>99 (*R*)
IRED-**I**	10	54	93 (*S*)	56	>99	96 (*S*)	61	87	97 (*S*)
IRED-**J**	99	>99	99 (*S*)	69	>99	99 (*S*)	89	>99	>99 (*S*)
IRED-**K**	30	>99	98 (*S*)	66	>99	98 (*S*)	80	90	>99 (*S*)
IRED-**L**	6	10	97 (*S*)	58	70	97 (*S*)	65	>99	>99 (*S*)
IRED-**M**	27	>99	97 (*S*)	61	>99	97 (*S*)	85	90	>99 (*S*)
IRED-**N**	7	12	88 (*S*)	38	54	53 (*S*)	19	69	92 (*S*)

Supplementary Table S1. Conversions and optical purities observed in the reduction of imines **1a**–**c** by IREDs **A**–**N**. *Reaction conditions:* Substrate **1** (10 mM), $NADP^+$ (1 mM), IRED (2 mg/mL crude preparation), *Lb*-ADH (2 mg/mL crude preparation), Tris-HCl buffer (100 mM, pH 7.5; IREDs **A**–**E**, **I**–**M**) or potassium phosphate buffer (100 mM, pH 6.0; IREDs **F**–**H**, **N**), 2-PrOH (5% v/v), 30 °C, 2–24 h.

**Substrate**	**1d**			**1e**			**1f**		
	conv. [%]		*ee* [%]	*conv.* [%]		*ee* [%]	*conv.* [%]		*ee* [%]
	2 h	24 h		2 h	24 h		2 h	24 h	
IRED-**A**	98	>99	13 (*S*)	>99	>99	*n.a.*	>99	>99	*n.a.*
IRED-**B**	77	>99	>99 (*S*)	–	36	*n.a.*	63	>99	*n.a.*
IRED-**C**	–	11	83 (*S*)	>99	>99	*n.a.*	>99	>99	*n.a.*
IRED-**D**	22	>99	96 (*S*)	>99	>99	*n.a.*	>99	>99	*n.a.*
IRED-**E**	99	>99	>99 (*S*)	>99	>99	*n.a.*	>99	>99	*n.a.*
IRED-**F**	–	–	–	>99	>99	*n.a.*	21	49	*n.a.*
IRED-**G**	24	89	7 (*S*)	>99	>99	*n.a.*	81	>99	*n.a.*
IRED-**H**	97	>99	82 (*R*)	78	>99	*n.a.*	93	>99	*n.a.*
IRED-**I**	2	40	91 (*R*)	>99	>99	*n.a.*	>99	>99	*n.a.*
IRED-**J**	5	32	3 (*R*)	>99	>99	*n.a.*	>99	>99	*n.a.*
IRED-**K**	2	24	16 (*R*)	>99	>99	*n.a.*	>99	>99	*n.a.*
IRED-**L**	–	–	–	>99	>99	*n.a.*	58	>99	*n.a.*
IRED-**M**	3	55	37 (*S*)	>99	>99	*n.a.*	>99	>99	*n.a.*
IRED-**N**	6	55	86 (*R*)	74	>99	*n.a.*	>99	>99	*n.a.*

Supplementary Table S2. Conversions and optical purities observed in the reduction of imines **1d**–**f** by IREDs **A**–**N**. *Reaction conditions:* Substrate **1** (10 mM), NADP+ (1 mM), IRED (2 mg/mL crude preparation), *Lb*-ADH (2 mg/mL crude preparation), Tris-HCl buffer (100 mM, pH 7.5; IREDs **A**–**E**, **I**–**M**) or potassium phosphate buffer (100 mM, pH 6.0; IREDs **F**–**H**, **N**), 2-PrOH (5% v/v), 30 °C, 2–24 h. *n.a.*: not applicable (achiral reduction product)

**Substrate**	**1g**			**1h**			**1i**		
	conv. [%]		*ee* [%]	*conv.* [%]		*ee* [%]	*conv.* [%]		*ee* [%]
	2 h	24 h		2 h	24 h		2 h	24 h	
IRED-**A**	84	>99	71 (*R*)	*n.d.*	–	–	69	82	13 (*R*)
IRED-**B**	16	19	*n.d.*	*n.d.*	–	–	5	5	*n.d.*
IRED-**C**	84	>99	>99 (*R*)	*n.d.*	–	–	35	>99	>99 (*S*)
IRED-**D**	84	>99	>99 (*R*)	*n.d.*	46	71 (*R*)	11	49	70 (*S*)
IRED-**E**	83	>99	>99 (*R*)	*n.d.*	31	58 (*R*)	7	6	*n.d.*
IRED-**F**	36	83	>99 (*R*)	*n.d.*	15		56	>99	>99 (*S*)
IRED-**G**	58	>99	>99 (*S*)	*n.d.*	49	>99 (*S*)	93	>99	>99 (*R*)
IRED-**H**	35	84	91 (*R*)	*n.d.*	–	–	17	35	36 (*R*)
IRED-**I**	51	96	>99 (*S*)	*n.d.*	>99	>99 (*S*)	92	93	>99 (*S*)
IRED-**J**	85	>99	>99 (*S*)	*n.d.*	>99	>99 (*S*)	71	94	68 (*R*)
IRED-**K**	23	76	>99 (*S*)	*n.d.*	>99	>99 (*S*)	93	93	78 (*S*)
IRED-**L**	85	>99	>99 (*S*)	*n.d.*	>99	>99 (*S*)	–	6	*n.d.*
IRED-**M**	85	>99	>99 (*S*)	*n.d.*	>99	>99 (*S*)	27	50	61 (*R*)
IRED-**N**	20	59	91 (*S*)	*n.d.*	–	–	93	>99	>99 (*S*)

Supplementary Table S3. Conversions and optical purities observed in the reduction of imines **1g**–**i** by IREDs **A**–**N**. *Reaction conditions:* Substrate **1** (10 mM), NADP+ (1 mM), IRED (2 mg/mL crude preparation), *Lb*-ADH (2 mg/mL crude preparation), Tris-HCl buffer (100 mM, pH 7.5; IREDs **A**–**E**, **I**–**M**) or potassium phosphate buffer (100 mM, pH 6.0; IREDs **F**–**H**, **N**), 2-PrOH (5% v/v), 30 °C, 2–24 h. *n.d.*: not determined

Substrate	1j			1k		
	conv. [%]		*ee* [%]	*conv.* [%]		*ee* [%]
	2 h	24 h		2 h	24 h	
IRED-**A**	1	8	93 (*S*)	*n.d.*	2	*n.d.*
IRED-**B**	–	–	–	*n.d.*	–	–
IRED-**C**	1	–	–	*n.d.*	1	*n.d.*
IRED-**D**	–	27	78 (*R*)	*n.d.*	5	1 (*R*)
IRED-**E**	2	1	*n.d.*	*n.d.*	1	*n.d.*
IRED-**F**	1	13	>99 (*R*)	*n.d.*	3	*n.d.*
IRED-**G**	–	21	>99 (*S*)	*n.d.*	6	>99 (*S*)
IRED-**H**	–	1	*n.d.*	*n.d.*	1	*n.d.*
IRED-**I**	61	90	>99 (*S*)	*n.d.*	9	>99 (*S*)
IRED-**J**	91	91	96 (*S*)	*n.d.*	86	92 (*S*)
IRED-**K**	92	91	>99 (*S*)	*n.d.*	88	>99 (*S*)
IRED-**L**	1	91	96 (*S*)	*n.d.*	88	>99 (*S*)
IRED-**M**	90	92	>99 (*S*)	*n.d.*	85	>99 (*S*)
IRED-**N**	91	5	>99 (*S*)	*n.d.*	2	*n.d.*

Supplementary Table S4. Conversions and optical purities observed in the reduction of imines **1j** and **1k** by IREDs **A**–**N**. *Reaction conditions:* Substrate **1** (10 mM), $NADP^+$ (1 mM), IRED (2 mg/mL crude preparation), *Lb*-ADH (2 mg/mL crude preparation), Tris-HCl buffer (100 mM, pH 7.5; IREDs **A**–**E**, **I**–**M**) or potassium phosphate buffer (100 mM, pH 6.0; IREDs **F**–**H**, **N**), 2-PrOH (5% v/v), 30 °C, 2–24 h. *n.d.*: not determined

Process Optimisation

Biotransformations at elevated substrate concentrations (10–500 mM) employing cell-free enzyme preparations are shown in Supplementary Figures S6–S9, those employing lyophilised cells of *E. coli* BL21 (DE3) co-expressing *Lb*-ADH and the respective IRED are shown in Supplementary Figures S10–S16.

(A) IRED-**A** (M4ZRJ3)

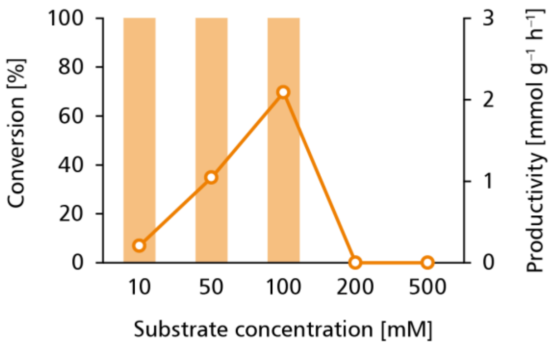

(B) IRED-**D** (V7GV82)

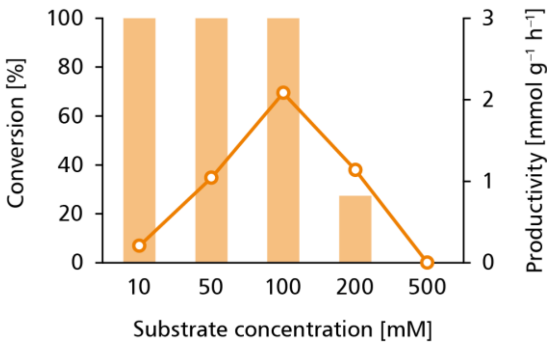

Supplementary Figure S6 (*continued on next page*).

(C) IRED-J (D2PR38)

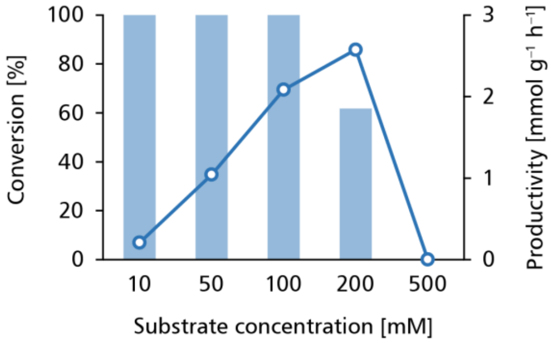

Supplementary Figure S6 (*continued from previous page*). Conversions (bars) and productivities (circles) achieved in the reduction of imine **1b** using different IREDs. Data from (*R*)-selective reductions are shown in orange, those from (*S*)-selective reductions are shown in blue. *Reaction conditions:* Substrate **1b** (10–500 mM), $NADP^+$ (1 mM), IRED (2 mg/mL crude preparation), *Lb*-ADH (2 mg/mL crude preparation), Tris-HCl buffer (100 mM, pH 7.5), 2-PrOH (5% v/v), 30 °C, 24 h.

(A) IRED-A (M4ZRJ3)

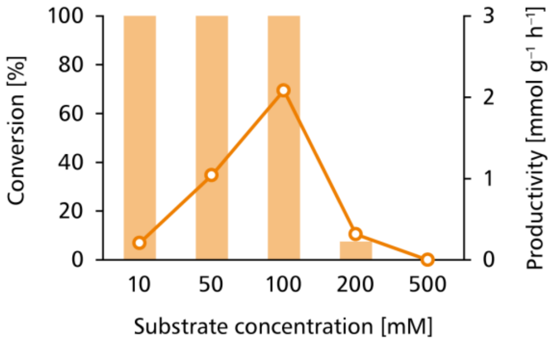

(B) IRED-D (V7GV82)

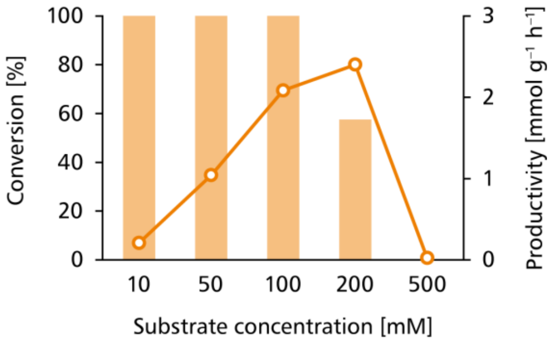

(C) IRED-J (D2PR38)

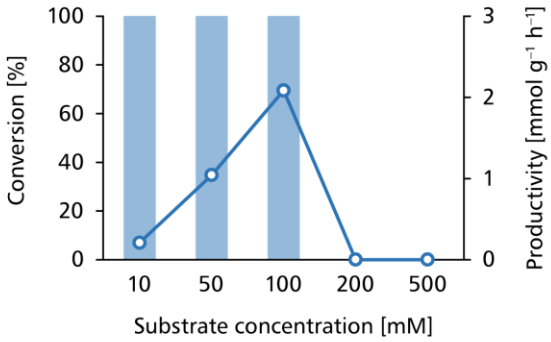

Supplementary Figure S7. Conversions (bars) and productivities (circles) achieved in the reduction of imine **1c** using different IREDs. Data from (*R*)-selective reductions are shown in orange, those from (*S*)-selective reductions are shown in blue. *Reaction conditions:* Substrate **1c** (10–500 mM), $NADP^+$ (1 mM), IRED (2 mg/mL crude preparation), *Lb*-ADH (2 mg/mL crude preparation), Tris-HCl buffer (100 mM, pH 7.5), 2-PrOH (5% v/v), 30 °C, 24 h.

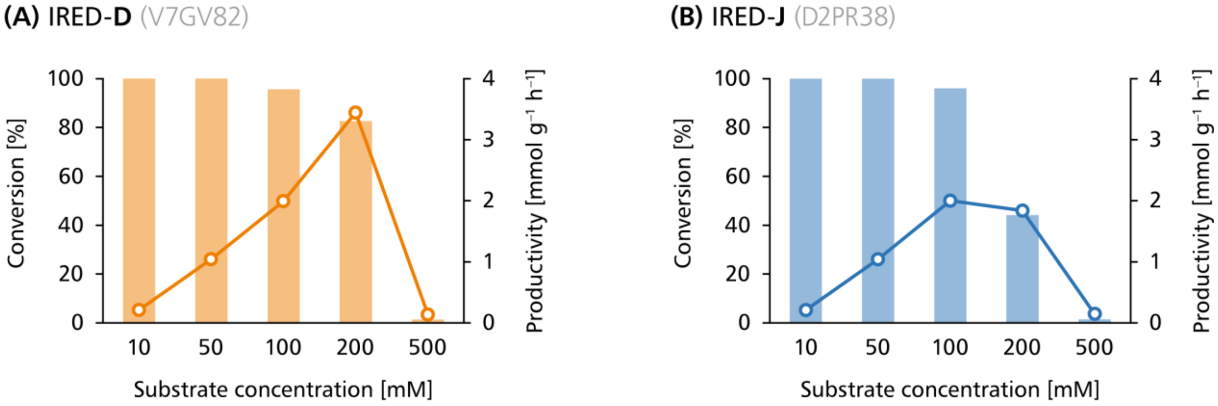

Supplementary Figure S8. Conversions (bars) and productivities (circles) achieved in the reduction of imine **1g** using different IREDs. Data from (*R*)-selective reductions are shown in orange, those from (*S*)-selective reductions are shown in blue. *Reaction conditions:* Substrate **1g** (10–500 mM), $NADP^+$ (1 mM), IRED (2 mg/mL crude preparation), *Lb*-ADH (2 mg/mL crude preparation), Tris-HCl buffer (100 mM, pH 7.5), 2-PrOH (5% v/v), 30 °C, 24 h.

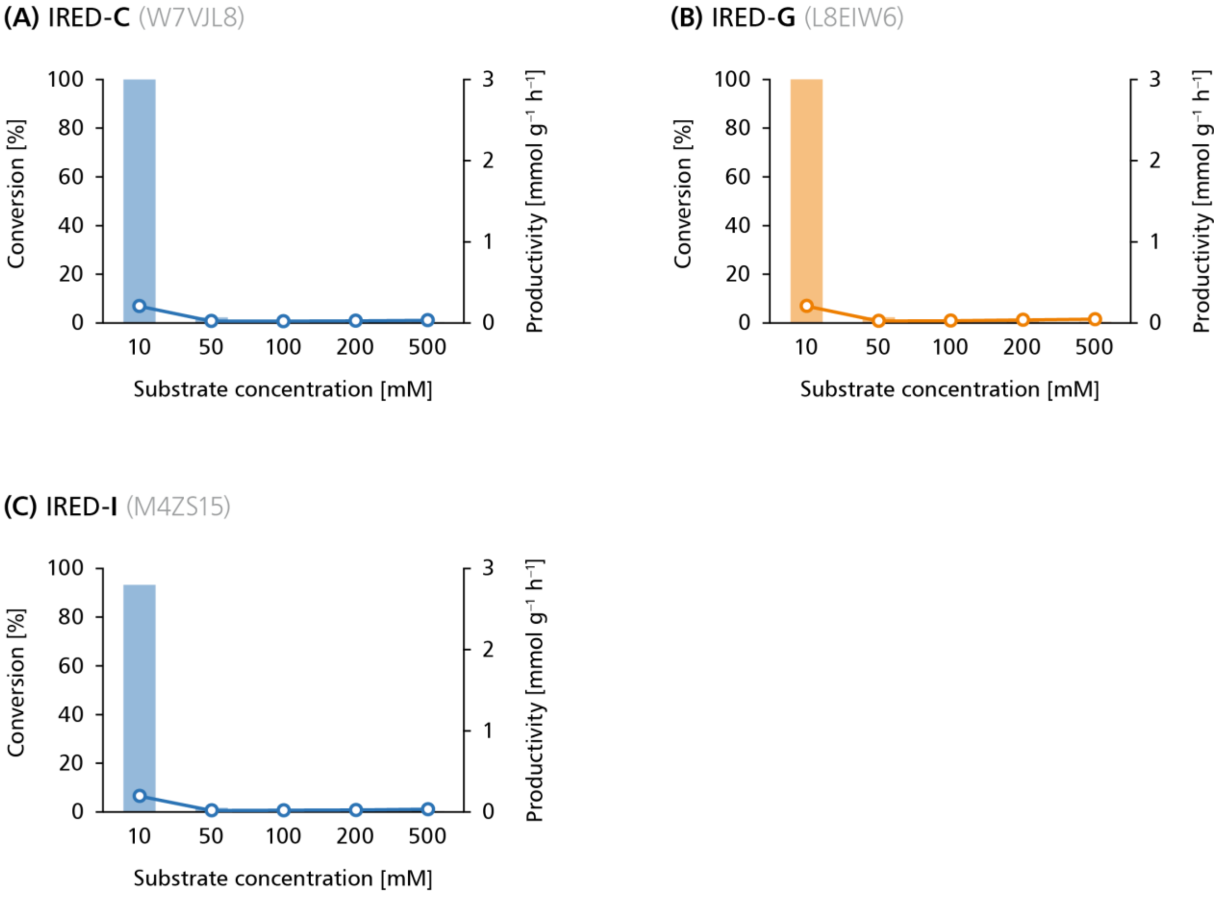

Supplementary Figure S9. Conversions (bars) and productivities (circles) achieved in the reduction of imine **1i** using different IREDs. Data from (*R*)-selective reductions are shown in orange, those from (*S*)-selective reductions are shown in blue. *Reaction conditions:* Substrate **1i** (10–500 mM), $NADP^+$ (1 mM), IRED (2 mg/mL crude preparation), *Lb*-ADH (2 mg/mL crude preparation), Tris-HCl buffer (100 mM, pH 7.5; IREDs **C** and **I**) or potassium phosphate buffer (100 mM, pH 6.0; IRED-**G**), 2-PrOH (5% v/v), 30 °C, 24 h.

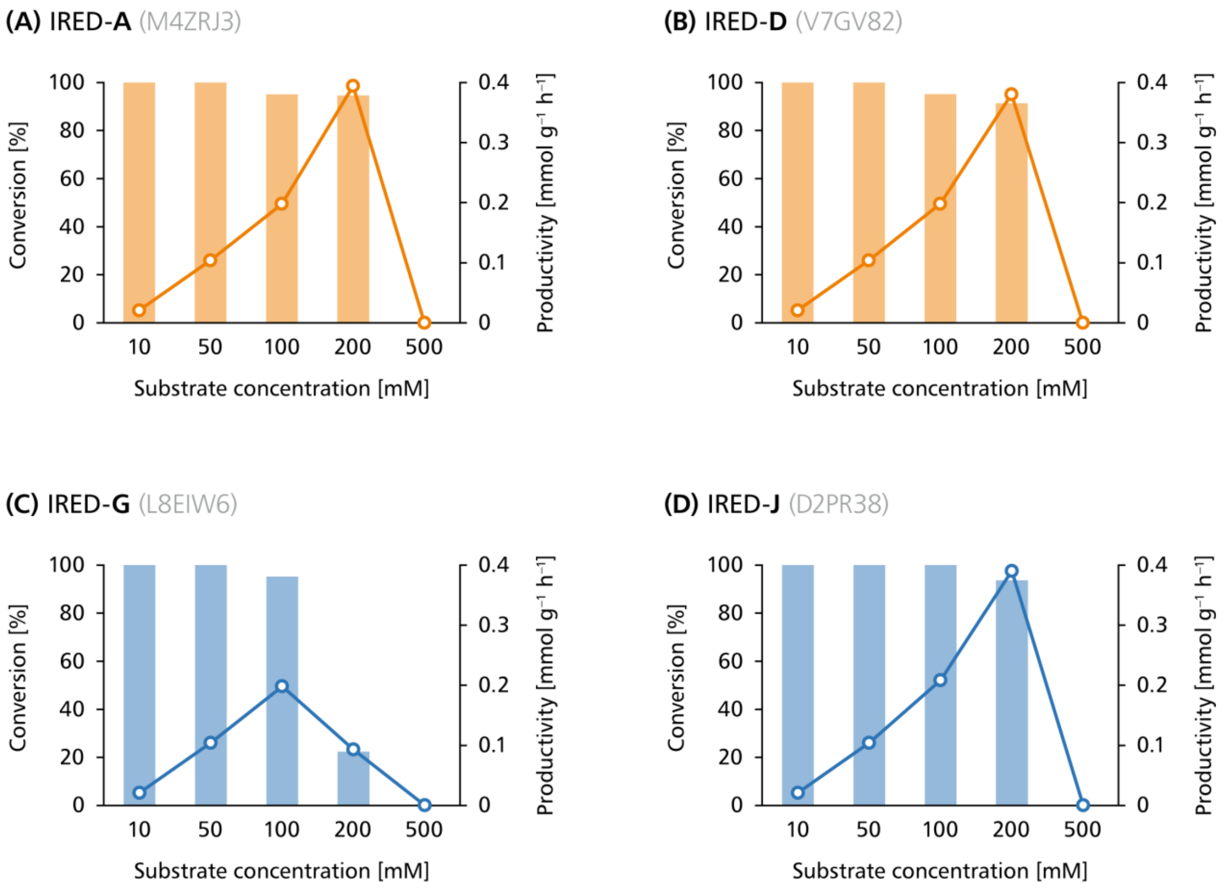

Supplementary Figure S10. Conversions (bars) and productivities (circles) achieved in the reduction of imine **1a** using a whole-cell biocatalyst co-expressing *Lb*-ADH and different IREDs. Data from (*R*)-selective reductions are shown in orange, those from (*S*)-selective reductions are shown in blue. *Reaction conditions:* Substrate **1a** (10–500 mM), *E. coli* BL21 (DE3) co-expressing IRED and *Lb*-ADH (20 mg/mL lyophilised cells), Tris-HCl buffer (100 mM, pH 7.5), 2-PrOH (5% v/v), 30 °C, 24 h.

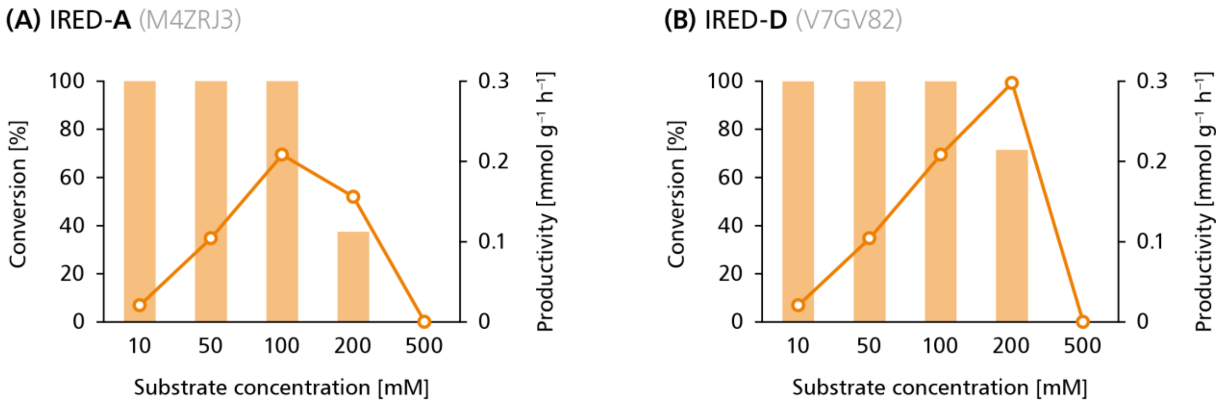

Supplementary Figure S11 (*continued on next page*).

(C) IRED-**J** (D2PR38)

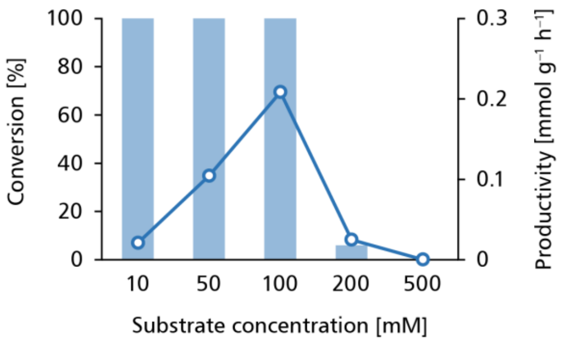

Supplementary Figure S11 (*continued from previous page*). Conversions (bars) and productivities (circles) achieved in the reduction of imine **1b** using a whole-cell biocatalyst co-expressing *Lb*-ADH and different IREDs. Data from (*R*)-selective reductions are shown in orange, those from (*S*)-selective reductions are shown in blue. *Reaction conditions:* Substrate **1b** (10–500 mM), *E. coli* BL21 (DE3) co-expressing IRED and *Lb*-ADH (20 mg/mL lyophilised cells), Tris-HCl buffer (100 mM, pH 7.5), 2-PrOH (5% v/v), 30 °C, 24 h.

(A) IRED-**A** (M4ZRJ3)

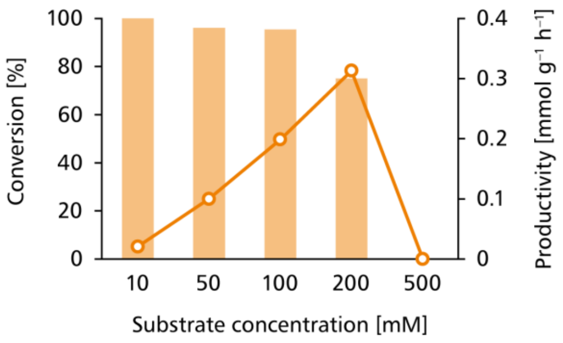

(B) IRED-**D** (V7GV82)

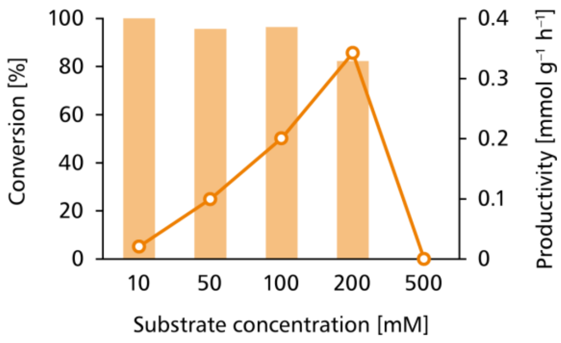

(C) IRED-**J** (D2PR38)

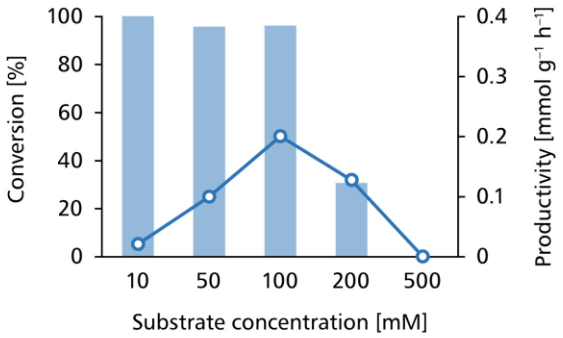

Supplementary Figure S12. Conversions (bars) and productivities (circles) achieved in the reduction of imine **1c** using a whole-cell biocatalyst co-expressing *Lb*-ADH and different IREDs. Data from (*R*)-selective reductions are shown in orange, those from (*S*)-selective reductions are shown in blue. *Reaction conditions:* Substrate **1c** (10–500 mM), *E. coli* BL21 (DE3) co-expressing IRED and *Lb*-ADH (20 mg/mL lyophilised cells), Tris-HCl buffer (100 mM, pH 7.5), 2-PrOH (5% v/v), 30 °C, 24 h.

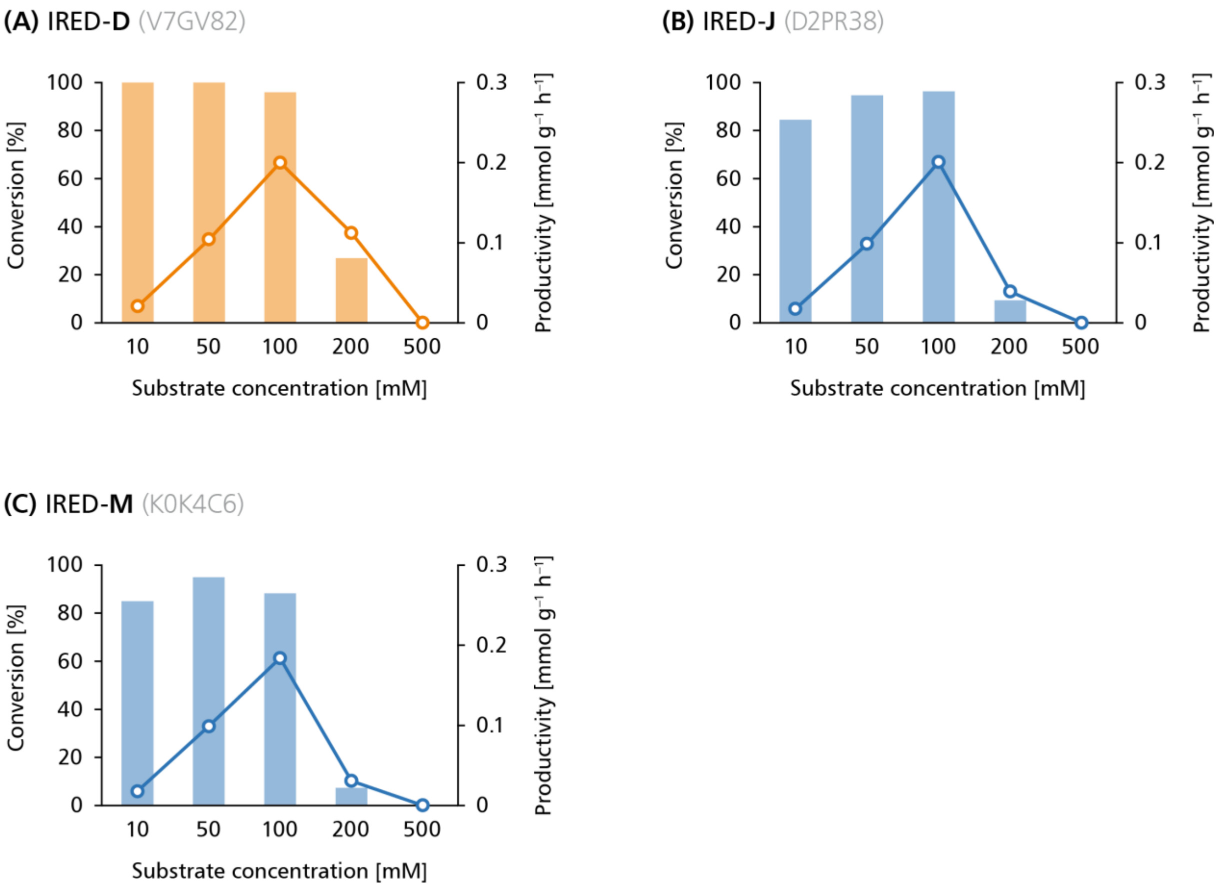

Supplementary Figure S13. Conversions (bars) and productivities (circles) achieved in the reduction of imine **1g** using a whole-cell biocatalyst co-expressing *Lb*-ADH and different IREDs. Data from (*R*)-selective reductions are shown in orange, those from (*S*)-selective reductions are shown in blue. *Reaction conditions:* Substrate **1g** (10–500 mM), *E. coli* BL21 (DE3) co-expressing IRED and *Lb*-ADH (20 mg/mL lyophilised cells), Tris-HCl buffer (100 mM, pH 7.5), 2-PrOH (5% v/v), 30 °C, 24 h.

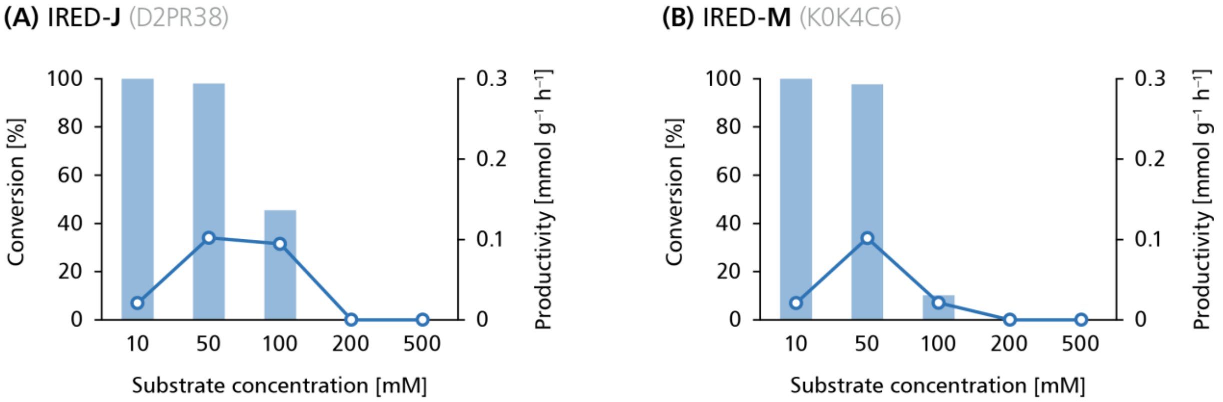

Supplementary Figure S14. Conversions (bars) and productivities (circles) achieved in the reduction of imine **1h** using a whole-cell biocatalyst co-expressing *Lb*-ADH and different IREDs. The (*S*)-enantiomer of the product was formed in all cases. *Reaction conditions:* Substrate **1h** (10–500 mM), *E. coli* BL21 (DE3) co-expressing IRED and *Lb*-ADH (20 mg/mL lyophilised cells), Tris-HCl buffer (100 mM, pH 7.5), 2-PrOH (5% v/v), 30 °C, 24 h.

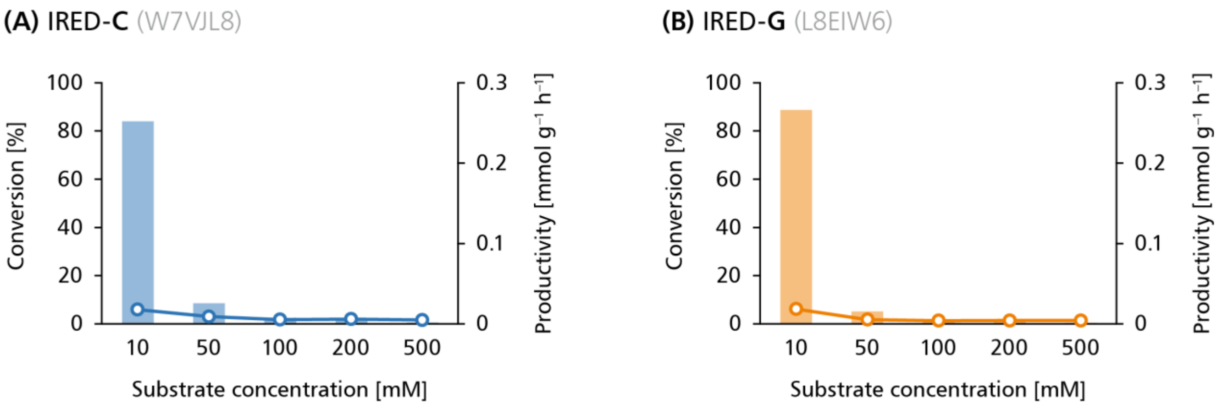

Supplementary Figure S15. Conversions (bars) and productivities (circles) achieved in the reduction of imine **1i** using a whole-cell biocatalyst co-expressing *Lb*-ADH and different IREDs. Data from (*R*)-selective reductions are shown in orange, those from (*S*)-selective reductions are shown in blue. *Reaction conditions:* Substrate **1i** (10–500 mM), *E. coli* BL21 (DE3) co-expressing IRED and *Lb*-ADH (20 mg/mL lyophilised cells), Tris-HCl buffer (100 mM, pH 7.5; IRED-**C**) or potassium phosphate buffer (100 mM, pH 6.0; IRED-**G**), 2-PrOH (5% v/v), 30 °C, 24 h.

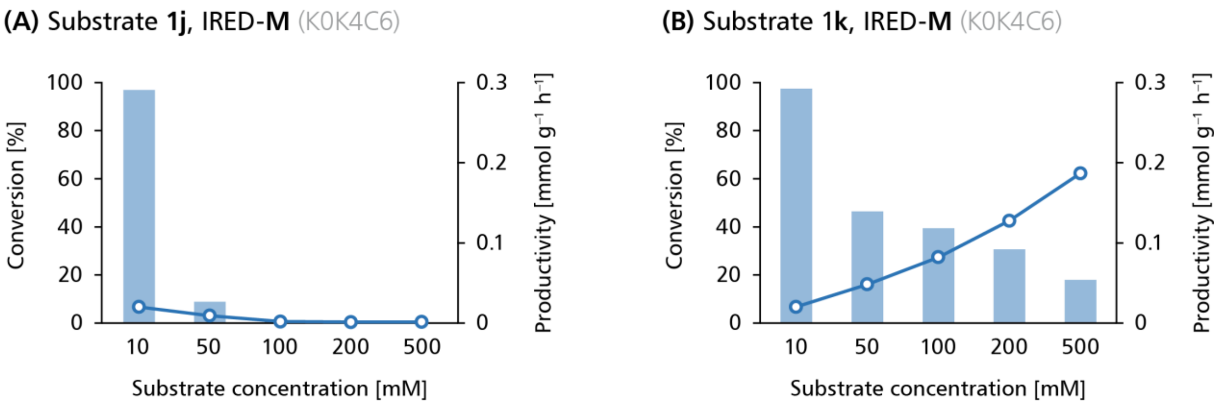

Supplementary Figure S16. Conversions (bars) and productivities (circles) achieved in the reduction of imines **1j** and **1k** using a whole-cell biocatalyst co-expressing *Lb*-ADH and IRED-**M**. The (*S*)-enantiomer of the product was formed in all cases. *Reaction conditions:* Substrate **1j** (10–500 mM), *E. coli* BL21 (DE3) co-expressing IRED and *Lb*-ADH (20 mg/mL lyophilised cells), Tris-HCl buffer (100 mM, pH 7.5; IRED-**C**) or potassium phosphate buffer (100 mM, pH 6.0; IRED-**G**), 2-PrOH (5% v/v), 30 °C, 24 h.

Supplementary Methods

Synthesis of Substrates and Reference Compounds

Imines **1a**, **1e**, **1g**, **1h**, **1i**, and **1k** as well as amines **2a**, **2b**, and **2e** were obtained from commercial suppliers and used as received. The other imines and amines were synthesised following procedures reported in the literature (in some cases with slight modifications).

6-Methyl-2,3,4,5-tetrahydropyridine (1b) [CAS 1462-92-6].[1] To an ice-cooled, stirred solution of *N*-chlorosuccinimide (NCS; 6.40 g, 47.9 mmol, 1.14 eq.) in anhydrous diethyl ether (90 mL) was added a solution of 2-methylpiperidine (**2a**; 4.18 g, 42.1 mmol, 1 eq.) in anhydrous diethyl ether (10 mL) dropwise over 30 min. The ice bath was then removed and the reaction mixture was stirred at room temperature for 28 h, at which time TLC (silica gel 60, MTBE:MeOH:NH_4OH = 90:9:1, basic permanganate staining) indicated completion of the reaction. The white precipitate of succinimide that had formed was removed by filtration through Celite, and the filtrate was washed with water (3 × 50 mL), dried over $MgSO_4$, filtered, and transferred to a round-bottom flask. To the stirred solution was added 1,8-diazabicyclo-[5.4.0]undec-7-ene (DBU; 7.2 mL, 7.31 g, 48 mmol, 1.14 eq.) and the resulting mixture was stirred at room temperature for 16 h, at which time TLC (silica gel 60 F_{254}, MTBE:MeOH:NH_4OH = 90:9:1, $KMnO_4$ staining) indicated completion of the reaction. The solvent was evaporated under reduced pressure to give 6.4 g of a brown liquid. Short-path distillation (Kugelrohr) afforded **1b** (2.32 g, 57%) as a colourless liquid. TLC (silica gel 60, MTBE:MeOH:NH_4OH = 90:9:1): R_f = 0.35. ^{1}H-NMR (300 MHz, $CDCl_3$): δ [ppm] = 1.49–1.57 (2H, m, CH_2), 1.61–1.69 (2H, m, CH_2), 1.89 (3H, t, J = 1.7 Hz, CH_3), 2.11 (2H, tt, J = 6.6 Hz, 1.9 Hz, CH_2–C=N), 3.48–3.53 (2H, m, CH_2–N). ^{13}C-NMR (75 MHz, $CDCl_3$): δ [ppm] = 19.5, 21.6, 27.4, 30.2, 49.0, 168.3. GC–MS (EI, 70 eV): m/z = 97 (M^+, 100), 69 (67), 56 (21), 42 (57). The characterisation data are in agreement with literature values.[1]

7-Methyl-3,4,5,6-tetrahydro-2*H*-azepine (1c) [CAS 3338-03-2].[2,3] To a stirred solution of ϵ-caprolactam (4.53 g, 40 mmol, 1 eq.) and triethylamine (5.85 mL, 4.25 g, 42 mmol, 1.05 eq.) in anhydrous diethyl ether (100 mL) under argon atmosphere was added trimethylsilyl chloride (TMSCl; 5.33 mL, 4.56 g, 42 mmol, 1.05 eq.) over 10 min *via* a syringe. The resulting mixture was heated to gentle reflux on a water bath for 2 h; afterwards, the water bath was removed and the reaction mixture was allowed to cool to room temperature. The white precipitate of triethylammonium chloride that had formed was removed by filtration through a glass frit. The filtrate was transferred to a three-necked round-bottom flask, put under an argon atmosphere and cooled to –50 °C on an EtOH/N_2(l) bath. A solution of methyl lithium in anhydrous diethyl ether (25 mL of a 1.6 M solution; 40 mmol, 1 eq.) was added over 15 min *via* a syringe, the cooling bath was removed, and the reaction mixture was stirred at room temperature overnight, resulting in the formation of a clear, yellow solution. TLC analysis of a 100 μL sample (silica gel 60 F_{254}, EtOAc, $KMnO_4$ staining) indicated complete consumption of ϵ-caprolactam. The reaction was quenched by addition of half-saturated aqueous NH_4Cl solution (50 mL), the phases were separated, and the aqueous phase was extracted with dichloromethane (3 × 50 mL). The combined organic phases were dried over $MgSO_4$, filtered, and the solvent was evaporated under reduced pressure to give 5.21 g of a yellowish liquid. Short-path distillation (Kugelrohr) afforded **1c** (1.58 g, 35%) as a colourless liquid. TLC (silica gel 60, EtOAc): R_f = 0.50. ^{1}H-NMR (300 MHz, $CDCl_3$): δ [ppm] = 1.40–1.55 (4H, m, 3 × CH_2), 1.74–1.82 (2H, m, CH_2), 2.04 (3H, s, CH_3), 2.34–2.38 (2H, m, CH_2–C=N), 3.50–3.54 (2H, m, CH_2–N). ^{13}C-NMR (75 MHz, $CDCl_3$): δ [ppm] = 23.0, 26.3, 28.9, 31.5, 34.3, 51.2, 176.9. GC–

MS (EI, 70 eV): m/z = 111 (M^+, 100), 96 (24), 83 (74), 68 (54), 55 (75), 42 (81). The characterisation data are in agreement with literature values.[2]

5-Phenyl-3,4-dihydro-2*H*-pyrrole (1d) [CAS 700-91-4].[3] To an ice-cooled, stirred solution of 2-pyrrolidone (3.40 g, 40 mmol, 1 eq.) and triethylamine (5.85 mL, 4.25 g, 42 mmol, 1.05 eq.) in anhydrous diethyl ether (100 mL) under argon atmosphere was added trimethylsilyl chloride (TMSCl; 5.33 mL, 4.56 g, 42 mmol, 1.05 eq.) over 20 min *via* a syringe. The resulting mixture was heated to gentle reflux on a water bath for 1 h; afterwards, the water bath was removed and the reaction mixture was allowed to cool to room temperature. The white precipitate of triethylammonium chloride that had formed was removed by filtration through a glass frit. The filtrate was transferred to a three-necked round-bottom flask and put under an argon atmosphere. A solution of phenylmagnesium bromide in anhydrous tetrahydrofuran (40 mL of a 1.0 M solution; 40 mmol, 1 eq.) was added over 15 min *via* a syringe. After the addition was complete, the reaction mixture was heated to reflux for 2 h. The heating source was then removed and the reaction mixture was stirred at room temperature overnight, resulting in the formation of a clear, orange solution. TLC analysis of a 100 μL sample (silica gel 60 F_{254}, EtOAc, UV visualisation, $KMnO_4$ staining) indicated complete consumption of 2-pyrrolidone and formation of a UV-active product. The reaction was quenched by addition of 1 M hydrochloric acid (10 mL) and the resulting mixture was basified (pH 10–11) by addition of 10% (w/v) aqueous NaOH solution (20 mL). The phases were separated and the aqueous phase was diluted with water (30 mL) and extracted with diethyl ether (2 × 50 mL). The combined organic phases were dried over $MgSO_4$, filtered, and the solvent was evaporated under reduced pressure to give 7.63 g of a yellowish liquid. Column chromatography (silica gel 60, hexanes:EtOAc = 3:1) afforded **1d** (1.29 g, 22%) as a pale-yellowish liquid. TLC (silica gel 60, EtOAc): R_f = 0.66. ^{1}H-NMR (300 MHz, $CDCl_3$): δ [ppm] = 2.00–2.10 (2H, m, CH_2–C=N), 2.96 (2H, ddt, J = 8.3 Hz, 7.3 Hz, 2.1 Hz, C$\underline{H}_2$–CH_2–C=N), 4.08 (2H, tt, J = 7.4 Hz, 2.1 Hz, CH_2–N), 7.41–7.44 (3H, m, Ar-*m*, Ar-*p*), 7.84–7.88 (2H, m, Ar-*o*). ^{13}C-NMR (75 MHz, $CDCl_3$): δ [ppm] = 22.7, 34.9, 61.5, 127.6, 128.4, 130.3, 134.6, 173.3. GC–MS (EI, 70 eV): m/z = 145 (M^+, 54), 117 (100), 104 (12), 77 (14). The characterisation data are in agreement with literature values.[3]

2-Methyl-3,4-dihydroisoquinolin-2-ium trifluoromethanesulfonate (1f).[4]
To a stirred solution of 3,4-dihydroisoquinoline (328 mg, 2.5 mmol, 1 eq.) in dichloromethane (2 mL) was added methyl trifluoromethanesulfonate (285 μL, 427 mg, 1.25 mmol, 1.04 eq.) *via* a pipette. The reaction mixture was stirred for 1 h, at which time TLC analysis (silica gel 60 F_{254}, EtOAc, UV visualisation) indicated complete consumption of 3,4-dihydroisoquinoline. The solvent was evaporated under reduced pressure, and the oily residue was stirred with 5 mL of pentane for 10 min and afterwards stored in the fridge overnight. The next day, the product had deposited as a pale-yellowish solid, which was collected by filtration through a small glass frit and dried in the desiccator overnight. **1f** (724 mg, 98%). ^{1}H-NMR (300 MHz, MeOH-d_4): δ [ppm] = 3.33 (2H, t, J = 8.2 Hz, C4-H_2), 3.83 (3H, s, CH_3), 4.10 (2H, t, J = 8.2 Hz, C3-H_2), 7.50–7.60 (2H, m, Ar), 7.80–7.84 (2H, m, Ar), 9.08 (1H, s, C1-H). ^{13}C-NMR (75 MHz, MeOH-d_4): δ [ppm] = 25.5, 49.8, 118.3, 122.5, 124.6, 128.1, 133.3, 136.2, 137.7, 167.1. The characterisation data are in agreement with literature values.[4]

1-Methyl-4,9-dihydro-3*H*-β-carboline (1j) [CAS 525-41-7].[5] To an ice-cooled, stirred solution of tryptamine (2.00 g, 12.5 mmol, 1 eq.) and triethylamine (4.0 mL, 2.90 g, 28.7 mmol, 2.3 eq.) in dichloromethane (40 mL) was added acetyl chloride (976 μL, 1.08 g, 13.8 mmol, 1.1 eq.) dropwise over 2 min *via* a pipette. The ice bath was then removed and the reaction mixture was stirred for 20 h at room temperature, at which time TLC analysis (silica gel 60 F_{254}, MTBE:MeOH:NH_4OH = 90:9:1, UV visualisation) indicated completion of the reaction. The

reaction was quenched by addition of water (30 mL), the phases were separated, and the aqueous phase was extracted with dichloromethane (20 mL). The combined organic phases were washed with 1 M hydrochloric acid (20 mL) and water (10 mL), dried over $MgSO_4$, and the solvent was evaporated under reduced pressure to give *N*-(2-(1*H*-indol-3-yl)ethyl)-acetamide (2.57 g, quant.) as an orange, highly viscous oil.

A solution of the crude intermediate (368 mg, 182 mmol, 1 eq.) and phosphorous oxychloride ($POCl_3$; 1 mL 1.65 g, 10.7 mmol, 5.9 eq.) in anhydrous acetonitrile (9 mL) under argon atmosphere was heated to 120 °C for 5 min in a glass tube sealed with a Teflon-fitted crimp cap using a *Biotage* Initiator laboratory microwave instrument. The resulting brown solution was allowed to cool to room temperature and water (10 mL) was carefully added. The resulting solution was concentrated under reduced pressure to remove the acetonitrile before being basified (pH 11–12) by addition of 10 M aqueous NaOH solution (approx. 5 mL). The product was extracted into dichloromethane (3 × 30 mL), the combined extracts were dried over $MgSO_4$, filtered, and the solvent was evaporated under reduced pressure to give 307 mg of an orange liquid. The microwave reaction was repeated with two larger batches of the intermediate (1.11 g, 5.47 mmol each; using 4.94 g, 32.2 mmol each of $POCl_3$ in 12 mL each of acetonitrile) and the crude products thus obtained were combined with the crude product of the first batch to give a combined 1.96 g of a brown foamy solid. Column chromatography (silica gel 60, MTBE→MTBE:MeOH = 85:15) afforded **1j** (0.63 g, 27%) as a yellowish foamy solid. TLC (silica gel 60, MTBE:MeOH:NH_4OH = 90:9:1): R_f = 0.28. mp = 180–181 °C; lit.[6] mp = 180–183 °C. ^{1}H-NMR (300 MHz, $CDCl_3$): δ [ppm] = 2.46 (3H, d, J = 1.0 Hz, CH_3), 2.96 (2H, t, J = 8.4 Hz, C<u>H</u>$_2$–CH_2–N), 3.96 (2H, t, J = 8.3 Hz, CH_2–C<u>H</u>$_2$–N), 7.21 (1H, t, J = 7.5 Hz, C6-H), 7.32 (1H, t, J = 7.6 Hz, C7-H), 7.47 (1H, d, J = 8.2 Hz, C8-H), 7.67 (1H, d, J = 7.9 Hz, C5-H), 10.56 (1H, br d, J = 20.2 Hz, NH). ^{13}C-NMR (75 MHz, $CDCl_3$): δ [ppm] = 19.5, 22.0, 48.0, 112.3, 116.4, 120.1, 120.2, 124.4, 125.5, 129.5, 137.2, 158.8. GC–MS (EI, 70 eV): m/z = 184 (M^+, 73), 183 (M^+–H, 100), 154 (12). The characterisation data are in agreement with literature values.[7]

Synthesis of amines 2c, 2d, and 2g–k by $NaBH_4$-reduction of the corresponding imines (General procedure). To a solution of imine **1** (0.2–0.5 mmol, 1 eq.) in methanol (500 μL) in a microcentrifuge tube (2 mL) was added sodium borohydride (38 mg, 1.0 mmol, 2–5 eq.). The vial was closed, shaken briefly to ensure proper mixing of the reagents, opened again (pressure build-up!), and covered with a laboratory tissue. The reaction mixture was allowed to stand at room temperature overnight. The reaction was then quenched by addition of half-saturated aqueous Na_2CO_3 solution (500 μL) and the product was extracted into ethyl acetate (3 × 500 μL). The combined organic phases were dried over $MgSO_4$, centrifuged (13,000 rpm, 1 min), and the supernatant was evaporated under reduced pressure to give the crude amine **2**, which was characterised by GC–MS analysis and used as reference compound without further purification.

2-Methylazepane (2c) [CAS 7496-99-3]. 20 mg (35%) clear, colourless liquid from 55.6 mg (0.5 mmol) of **1c**. GC–MS (EI, 70 eV): m/z = 113 (M^+, 22), 98 (85), 84 (27), 70 (100), 57 (48), 41 (21).

2-Phenylpyrrolidine (2d) [CAS 1006-64-0]. 36 mg (49%) pale-yellowish liquid from 72.6 mg (0.5 mmol) of **1d**. GC–MS (EI, 70 eV): m/z = 147 (M^+, 28), 146 (M^+–H, 53), 118 (100), 70 (31).

1-Methyl-1,2,3,4-tetrahydroisoquinoline (2g) [CAS 4965-09-7]. 27 mg (92%) clear, colourless liquid from 40 mg (0.2 mmol) of **1g**. GC–MS (EI, 70 eV): m/z = 147 (M^+, 2), 146 (M^+–H, 11), 132 (M^+–CH_3, 100), 117 (19), 115 (10), 105 (9), 77(6).

6,7-Dimethoxy-1-methyl-1,2,3,4-tetrahydroisoquinoline (Salsolidine, 2h) [CAS 493-48-1]. 36 mg (87%) pale-yellowish liquid from 41 mg (0.2 mmol) of **1h**. GC–MS (EI, 70 eV): m/z = 207 (M^+, 7), 206 (M^+–H, 9), 192 (M^+–CH_3, 100), 176 (12), 148 (5).

2,3,3-Trimethylindoline (2i) [CAS 18781-58-3]. 45 mg (57%) yellowish liquid from 80 mg (0.5 mmol) of **1i**. GC–MS (EI, 70 eV): m/z = 161 (M^+, 33), 146 (100), 131 (42).

1-Methyl-2,3,4,9-tetrahydro-β-carboline (Eleagnine, 2j) [CAS 2506-10-7]. 11 mg (30%) yellowish solid from 37 mg (0.2 mmol) of **1j**. GC–MS (EI, 70 eV): m/z = 186 (M^+, 55), 171 (M^+–CH_3, 100), 157 (42), 156 (38), 144 (14), 130 (14), 115 (8), 85 (10).

7-Methoxy-1-methyl-2,3,4,9-tetrahydro-β-carboline (Leptaflorine, 2k) [CAS 486-93-1]. 12 mg (28%) white solid from 43 mg (0.2 mmol) of **1k**. GC–MS (EI, 70 eV): m/z = 216 (M^+, 41), 201 (M^+–CH_3, 100), 187 (18), 186 (18), 172 (20).

2-Methyl-1,2,3,4-tetrahydroisoquinoline (2f) [CAS 1612-65-3]. To a solution of 1,2,3,4-tetrahydroisoquinoline (66.6 mg, 0.5 mmol, 1 eq.) in 2-propanol (500 μL) in a microcentrifuge tube (2 mL) were added sodium cyanoborohydride (212 mg, 1.0 mmol, 2 eq.) and paraformaldehyde (30 mg, 1.0 mmol, 2 eq.). The vial was closed, shaken briefly to ensure proper mixing of the reagents, and incubated in a Thermoshaker at 30 °C and 500 rpm overnight. The reaction was then quenched by addition of 6 M hydrochloric acid (20 μL) and the resulting solution was basified by addition of saturated aqueous Na_2CO_3 solution (500 μL). The product was extracted into ethyl acetate (3 × 500 μL), the combined organic phases were dried over $MgSO_4$, centrifuged (13,000 rpm, 1 min), and the supernatant was evaporated under reduced pressure to give 30 mg (41%) of the crude amine **2f** as a yellowish liquid, which was characterised by GC–MS analysis and used as reference compound without further purification. GC–MS (EI, 70 eV): m/z = 147 (M^+, 42), 146 (M^+–H, 100), 104 (52).

Gene Synthesis and Subcloning

Gene sequences encoding the investigated IREDs were obtained from the Genbank database entries associated with the corresponding protein sequence entries in the UniProt database. Synthetic genes were obtained from *Invitrogen* (GeneArt®; now part of *Thermo Fisher Scientific*) in the form of linear double-stranded DNA (GeneArt® Strings™), in which the coding sequence was codon-optimised for expression in *E. coli*, supplemented with *Nde*I and *Xho*I restriction sites at the 5' and 3' ends, respectively, and flanked by a 15-bp "stuffer DNA" at both ends to enable subcloning into a pET28a(+) vector (*Novagen*). The integrity of the obtained constructs was verified by DNA sequencing (*LGC Genomics*, Berlin, Germany).

The gene sequence encoding *Lb*-ADH was obtained from the GenBank database (accession code: AJ544275.1). A synthetic gene was obtained from *Invitrogen* (GeneArt®; now part of *Thermo Fisher Scientific*) in the form of a 'working plasmid', in which the coding sequence was codon-optimised for expression in *E. coli* and flanked by *Nde*I and *Xho*I restriction sites at the 5' and 3' ends, respectively. The gene was excised and subcloned into a pET21a(+) vector (*Novagen*) and the integrity of the obtained construct was verified by DNA sequencing (*LGC Genomics*, Berlin, Germany). For re-cloning into pASK-IBA5plus, the gene was amplified by overhang PCR using the primers *Lb*-ADH_fw (TATTAAGGTCTCGGCGCCATGAGCAATCGTCTGGAT) and *Lb*-ADH_rv (GCTACTGGTCTCATATCAATTCTGTGCGGTATAACCACCATCCAC), introducing *Eco*31I restriction sites at both ends of the gene. The PCR product was restricted using FastDigest *Eco*31I (*Thermo Fisher Scientific*) and subcloned into a pASK-IBA5plus vector (*IBA Life Sciences*). A non-native C-terminal Asn residue was deleted by mutagenesis PCR using the primers *Lb*-

ADH_N252*_fw (GGTGGTTATACCGCACAGTAATGATATCTAAC) and *Lb*-ADH_N252*_rv (GTTAGATATCATTACTGTGCGGTATAACCACC) and the integrity of the obtained construct was verified by DNA sequencing (*LGC Genomics*, Berlin, Germany).

DNA and amino acid sequences of all investigated proteins can be found in a later section of this Electronic Supplementary Information. The plasmids used in the present study are listed in Supplementary Table S5:

			**Restriction Sites**			
**Internal ID**	**Vector**	**Insert**	5′	3′	**Antibiotic**	**Inducer**[a]
pEG48	pET21a(+)	*Lb*-ADH	*Nde*I	*Xho*I	Ampicillin	IPTG
pEG180	pASK-IBA5plus	*Lb*-ADH	*Eco*31I	*Eco*31I	Ampicillin	AHT
pEG373	pET28a(+)	IRED-**A** (M4ZRJ3)	*Nde*I	*Xho*I	Kanamycin	IPTG
pEG374	pET28a(+)	IRED-**B** (Q1EQE0)	*Nde*I	*Xho*I	Kanamycin	IPTG
pEG375	pET28a(+)	IRED-**C** (W7VJL8)	*Nde*I	*Xho*I	Kanamycin	IPTG
PEG376	pET28a(+)	IRED-**D** (V7GV82)	*Nde*I	*Xho*I	Kanamycin	IPTG
pEG377	pET28a(+)	IRED-**E** (J7LAY5)	*Nde*I	*Xho*I	Kanamycin	IPTG
pEG378	pET28a(+)	IRED-**F** (V6KA13)	*Nde*I	*Xho*I	Kanamycin	IPTG
pEG379	pET28a(+)	IRED-**G** (L8EIW6)	*Nde*I	*Xho*I	Kanamycin	IPTG
pEG380	pET28a(+)	IRED-**H** (I8QLV7)	*Nde*I	*Xho*I	Kanamycin	IPTG
pEG381	pET28a(+)	IRED-**I** (M4ZS15)	*Nde*I	*Xho*I	Kanamycin	IPTG
pEG382	pET28a(+)	IRED-**J** (D2PR38)	*Nde*I	*Xho*I	Kanamycin	IPTG
pEG383	pET28a(+)	IRED-**K** (D2AWI4)	*Nde*I	*Xho*I	Kanamycin	IPTG
pEG384	pET28a(+)	IRED-**L** (K0F8R0)	*Nde*I	*Xho*I	Kanamycin	IPTG
pEG385	pET28a(+)	IRED-**M** (K0K4C6)	*Nde*I	*Xho*I	Kanamycin	IPTG
pEG386	pET28a(+)	IRED-**N** (J7YM26)	*Nde*I	*Xho*I	Kanamycin	IPTG

Supplementary Table S5. Expression vectors used in the present study. [a] IPTG, isopropyl β-D-1-thiogalactopyranoside; AHT, anhydrotetracycline

Protein Expression and Purification

Expression of IREDs in *E. coli* BL21(DE3):

Chemically competent cells of *E. coli* BL21(DE3) (*New England Biolabs*) were transformed with the plasmids encoding the investigated IREDs according to the cell supplier's instructions. A single colony from a transformant agar plate was used to inoculate LB medium (15 mL, cont. 50 μg/mL kanamycin) and the resulting culture was incubated at 30 °C and 120 rpm overnight in a closed plastic tube (50 mL). Glycerol stocks were prepared by diluting the cell suspension of the overnight culture (700 μL) with sterile 60% (v/v) aqueous glycerol (300 μL) and were stored at –20 °C (working stock) and –80 °C (backup stock). For expression, LB medium (15 mL, cont. 50 μg/mL kanamycin) was inoculated with 50 μL of cell suspension from a glycerol stock and the resulting culture was incubated at 30 °C and 120 rpm overnight in a closed plastic tube (50 mL). 1 mL of this overnight culture was used to inoculate TB medium (100 mL, cont. 50 μg/mL kanamycin) and the resulting culture was incubated at 30 °C and 120 rpm in a baffled Erlenmeyer flask (300 mL). Samples (1 mL) of the culture were taken at regular intervals and analysed for their optical density at 600 nm (OD_{600}). When the OD_{600} reached a value of 0.8–1.0 (approx. after 3.5 h), protein expression was induced by the addition of IPTG (1 mM) and incubation was continued at 20 °C and 120 rpm overnight. The culture was transferred to a centrifuge beaker (500 mL) and centrifuged (8,000 rpm, 4 °C, 20 min) to pellet the cells. The supernatant was discarded and the cell pellet was resuspended in potassium phosphate buffer (20 mM, pH 7.0). The cell suspension was transferred to a plastic tube (50 mL), centrifuged again (4,000 rpm, 4 °C, 20 min), and the supernatant was again discarded. The resulting cell pellet (typically 0.8–1.3 g) was stored at –20 °C until use.

Cell disruption:
The cell pellet was thawed and resuspended in Tris-HCl buffer (10 mL/g cell weight; 100 mM, pH 7.5; for preparation of crude cell-free extracts) or HisTrap buffer A (10 mL/g cell weight; potassium phosphate buffer, 100 mM, pH 7.0, 300 mM NaCl; for protein purification). The resulting cell suspension was supplemented with lysozyme (1 mg/mL), incubated at 30 °C for 30 min and cooled to 0 °C on ice. The cells were disrupted by ultrasonication using a *Branson* Digital Sonifier 250 at 20% amplitude (50 W), 2 s pulse, 4 s pause, for a total pulse time of 2:30 min (75 cycles). The resulting sample was transferred to a centrifuge tube (50 mL) and cell debris was pelleted by centrifugation (16,000 rpm, 4 °C, 20 min). The supernatant was either lyophilised overnight to give a lyophilised, crude cell-free extract or subjected to protein purification by immobilised-metal affinity chromatography (IMAC) as described below.

Purification of IREDs by immobilised-metal affinity chromatography (IMAC):
A 5 mL HisTrap FF column (*GE Healthcare*; Ni^{2+} on NTA-modified cross-linked agarose) was equilibrated with HisTrap buffer A (100 mL; potassium phosphate buffer, 100 mM, pH 7.0, 300 mM NaCl). The supernatant obtained by cell disruption of IRED-expressing *E. coli* BL21(DE3) cells was loaded onto the column using a syringe and a 0.45 μm syringe filter for removal of insoluble matter. The column was then connected to a *Biorad* BioLogic DuoFlow FPLC system equipped with a BioFrac fraction collector and eluted using the following gradient elution programme while collecting fractions of 10 mL: 50 mL of 95% HisTrap buffer A / 5% HisTrap buffer B (potassium phosphate buffer, 100 mM, pH 7.0, 300 mM NaCl, 500 mM imidazole); 100 mL linear gradient of 95% A / 5% B to 100% B; 50 mL of 100% HisTrap buffer B. Alternatively, a step-elution protocol could be used: 75 mL of 95% HisTrap buffer A / 5% HisTrap buffer B, 75 mL of 85% A / 15% B, 50 mL of 40% A / 60% B (IRED elutes in this step), 50 mL of 100% HisTrap buffer B. The protein-containing fractions were assayed for IRED activity using the photometric method described in the main paper. The active fractions were pooled, concentrated using *Sartorius* VivaSpin 20 centrifugal filters with a 10 kDa molecular weight cut-off, and desalted using a *GE Healthcare* PD-10 column and Tris-HCl buffer (100 mM, pH 7.5) as eluent. In the case of IREDs **B**, **E**, and **N**, a buffer with a higher ionic strength (Tris-HCl, 100 mM, pH 7.5, 300 mM NaCl) was used in the desalting step to avoid precipitation of the protein. The desalted protein solution was analysed for protein concentration using the *Biorad* Bradford protein assay, lyophilised overnight, and stored at –20 °C until use.

A representative FPLC chromatogram is shown in Supplementary Figure S17 and the corresponding SDS-PAGE gel is shown in Supplementary Figure S18 (overleaf).

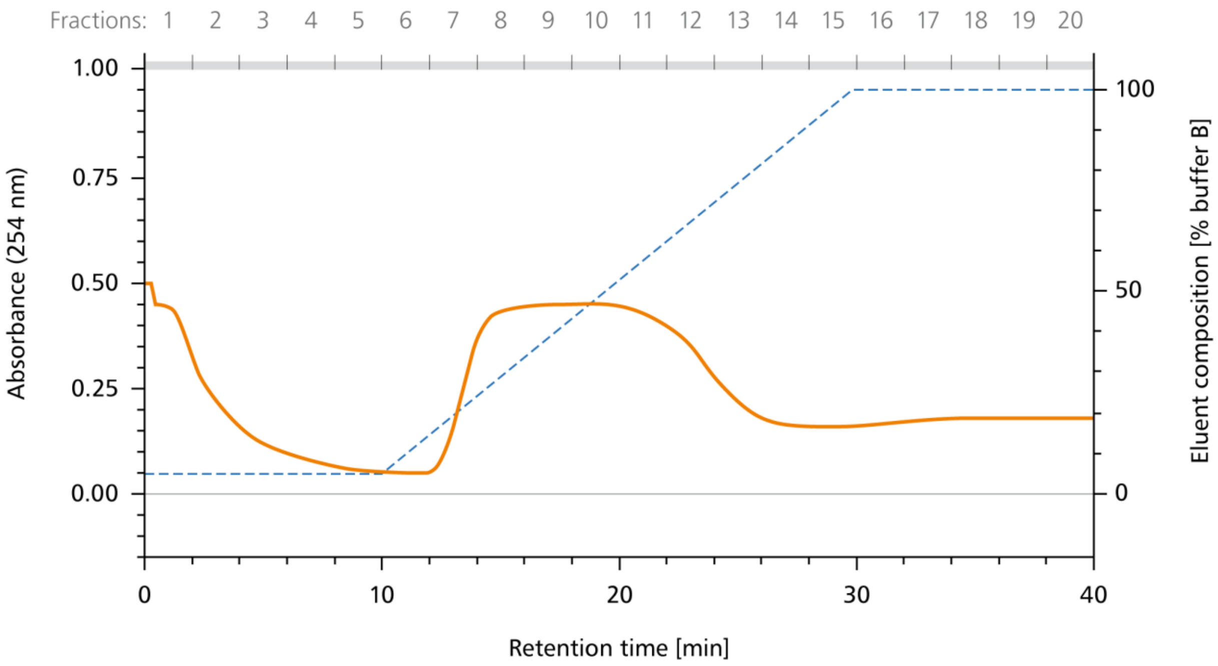

Supplementary Figure S17. FPLC chromatogram for the HisTrap purification of IRED-**I**, showing eluent composition (dashed blue line) and absorbance reading (solid orange line).

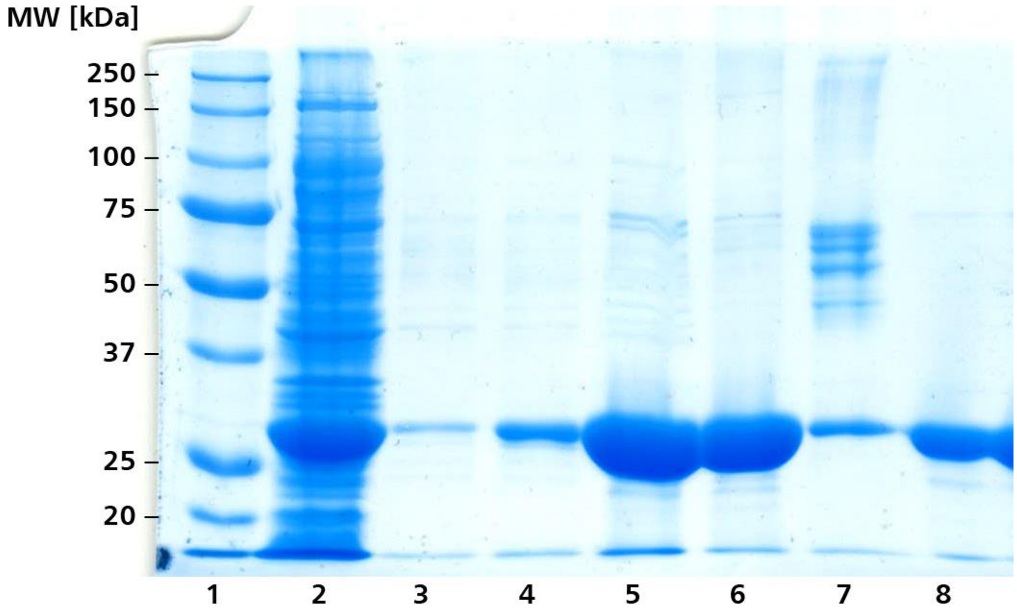

Supplementary Figure S18. SDS-PAGE of the HisTrap purification of IRED-**I**. Lanes: **1**, molecular weight standard (*Biorad* Precision Plus); **2**, column flow-through; **3**, fraction 5; **4**, fraction 7; **5**, fraction 9; **6**, fraction 11; **7**, fraction 14; **8**, pooled IRED-containing fractions after desalting

Expression of *Lb*-ADH in *E. coli* BL21(DE3):
Chemically competent cells of *E. coli* BL21(DE3) (*New England Biolabs*) were transformed with the [pASK-IBA5plus/*Lb*-ADH] plasmid according to the cell supplier's instructions. A single colony from a transformant agar plate was used to inoculate LB medium (15 mL, cont. 100 μg/mL ampicillin) and the resulting culture was incubated at 30 °C and 120 rpm overnight in a closed plastic tube (50 mL). Glycerol stocks were prepared by diluting the cell suspension of the overnight culture (700 μL) with sterile 60% (v/v) aqueous glycerol (300 μL) and were stored at –20 °C (working stock) and –80 °C (backup stock). For expression, LB medium (15 mL, cont. 100 μg/mL ampicillin) was inoculated with 50 μL of cell suspension from a glycerol stock and the resulting culture was incubated at 30 °C and 120 rpm overnight in a closed plastic tube (50 mL). 3 mL of this overnight culture were used to inoculate TB medium (300 mL, cont. 100 μg/mL kanamycin and 1 mM $MgCl_2$) and the resulting culture was incubated at 30 °C and 120 rpm in a baffled Erlenmeyer flask (300 mL). Samples (1 mL) of the culture were taken at regular intervals and analysed for their optical density at 600 nm (OD_{600}). When the OD_{600} reached a value of 0.8–1.0 (approx. after 3.5 h), protein expression was induced by the addition of anhydrotetracyclin (AHT, 0.2 mg/L) and incubation was continued at 20 °C and 120 rpm overnight. The culture was transferred to a centrifuge beaker (500 mL) and centrifuged (8,000 rpm, 4 °C, 20 min) to pellet the cells. The supernatant was discarded and the cell pellet was resuspended in Tris-HCl buffer (100 mM, pH 7.5). The cell suspension was transferred to a plastic tube (50 mL), centrifuged again (4,000 rpm, 4 °C, 20 min), and the supernatant was again discarded. The resulting cell pellet (typically 4.5–5.0 g) was stored at –20 °C until use.

A lyophilised crude cell-free extract was prepared as described above ('Cell disruption').

Co-expression of *Lb*-ADH and IREDs in *E. coli* BL21(DE3):
LB medium (200 mL, cont. 100 μg/mL ampicillin) was inoculated with 50 μL of cell suspension from a glycerol stock of *E. coli* BL21(DE3) [pASK-IBA5plus/*Lb*-ADH] and the resulting culture was used for the preparation of competent cells by the calcium chloride method.[8] The resulting competent cells were transformed with the plasmids encoding the investigated IREDs, using both ampicillin (100 μg/mL) and kanamycin (50 μg/mL) for selection. A single colony from a transformant agar plate was used to inoculate LB medium (15 mL, cont. 50 μg/mL kanamycin) and the resulting culture was incubated at 30 °C and 120 rpm overnight in a closed plastic tube (50 mL). Glycerol stocks were prepared by diluting the cell suspension of the overnight culture (700 μL) with sterile 60% (v/v) aqueous glycerol (300 μL) and were stored at –20 °C (working stock) and –80 °C (backup stock). For expression, LB medium (15 mL, cont. 100 μg/mL ampicillin and 50 μg/mL kanamycin) was inoculated with 50 μL of cell suspension from a glycerol stock and the resulting culture was incubated at 30 °C and 120 rpm overnight in a closed plastic tube (50 mL). 3 mL of this overnight culture were used to inoculate TB medium (300 mL, cont. 100 μg/mL ampicillin, 50 μg/mL kanamycin, and 1 mM $MgCl_2$) and the resulting culture was incubated at 30 °C and 120 rpm in a baffled Erlenmeyer flask (300 mL). Samples (1 mL) of the culture were taken at regular intervals and analysed for their optical density at 600 nm (OD_{600}). When the OD_{600} reached a value of 0.8–1.0 (approx. after 5 h), protein expression was induced by the addition of IPTG (1 mM) and AHT (0.2 mg/L) and incubation was continued at 20 °C and 120 rpm overnight. The culture was transferred to a centrifuge beaker (500 mL) and centrifuged (8,000 rpm, 4 °C, 20 min) to pellet the cells. The supernatant was discarded and the cell pellet was resuspended in Tris-HCl buffer (20 mL; 100 mM, pH 7.5, 1 mM $MgCl_2$). The cell suspension was transferred to a round-bottom flask, flash-frozen in liquid nitrogen, and lyophilised overnight to afford a dry cell powder (typically 1.0–1.5 g), which was stored at 4 °C until use.

Analytical Methods

Gas chromatography (achiral stationary phase):

Achiral-phase GC analyses were carried out on an *Agilent* 7890A GC system equipped with a flame-ionisation detector (FID) and either an *Agilent J&W* HP-5 capillary column (dimensions: 30 m × 0.32 mm × 0.25 μm; stationary phase: bonded & cross-linked 5%-phenyl-methylpoly-siloxane) or an *Agilent J&W* DB1701 capillary column (dimensions: 30 m × 0.25 mm × 0.25 μm; stationary phase: bonded & cross-linked 14%-cyanopropylphenyl-methylpolysiloxane), using helium (1 mL/min flow rate) as carrier gas. Compounds were quantified based on calibration curves that were set up using *n*-dodecane (10 mM in EtOAc used for extraction) as internal standard.

**Method GC-A1 (1a, 1b, 1c)**	
column:	HP-5 (30 m × 0.32 mm × 0.25 μm)
injector temperature:	300 °C
detector temperature:	300 °C
split ratio:	30:1
oven temperature program:	40 °C, 0.5 min; 10 °C/min to 90 °C; 30 °C/min to 300 °C; 300 °C, 1 min
total run time:	13.5 min

**Method GC-A2 (1d, 1e, 1f)**	
column:	HP-5 (30 m × 0.32 mm × 0.25 μm)
injector temperature:	300 °C
detector temperature:	300 °C
split ratio:	15:1
oven temperature program:	60 °C, 0.5 min; 10 °C/min to 160 °C; 40 °C/min to 300 °C; 300 °C, 1 min
total run time:	15 min

**Method GC-A3 (1g, 1h)**	
column:	DB-1701 (30 m × 0.25 mm × 0.25 μm)
injector temperature:	280 °C
detector temperature:	280 °C
split ratio:	15:1
oven temperature program:	80 °C, 1 min; 10 °C/min to 250 °C; 250 °C, 1 min
total run time:	19 min

**Method GC-A4 (1i, 1j)**	
column:	HP-5 (30 m × 0.32 mm × 0.25 μm)
injector temperature:	280 °C
detector temperature:	280 °C
split ratio:	15:1
oven temperature program:	80 °C, 1 min; 10 °C/min to 250 °C; 250 °C, 1 min
total run time:	19 min

**Method GC-A5 (1k)**	
column:	HP-5 (30 m × 0.32 mm × 0.25 μm)
injector temperature:	300 °C
detector temperature:	300 °C
split ratio:	15:1
oven temperature program:	140 °C, 1 min; 10 °C/min to 300 °C; 300 °C, 1 min
total run time:	18 min

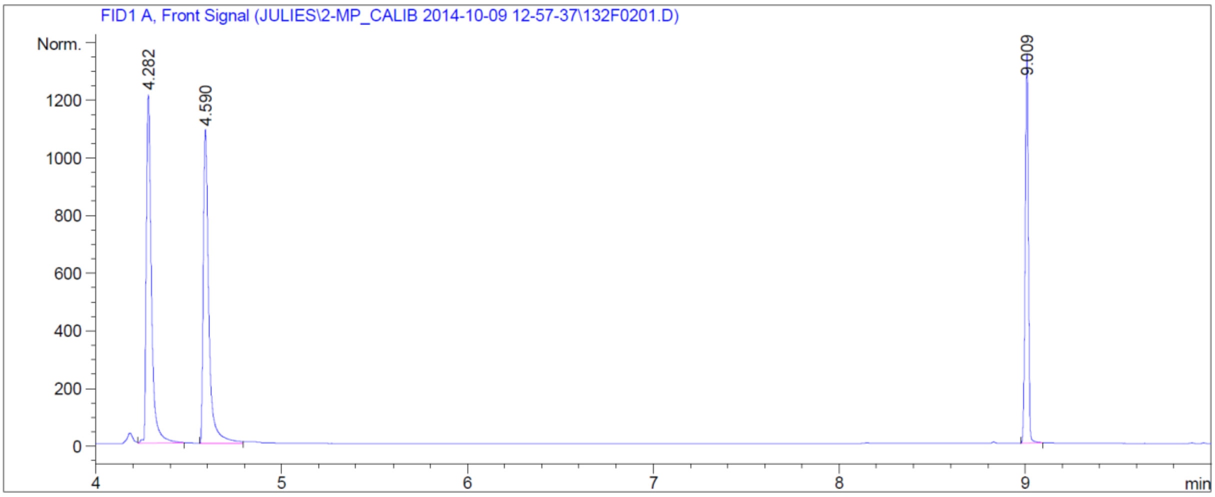

Supplementary Figure S19. Chromatographic separation of imine **1a** (t_r 4.6 min), amine **2a** (t_r 4.3 min), and *n*-dodecane (t_r 9.0 min) using GC method GC-A1.

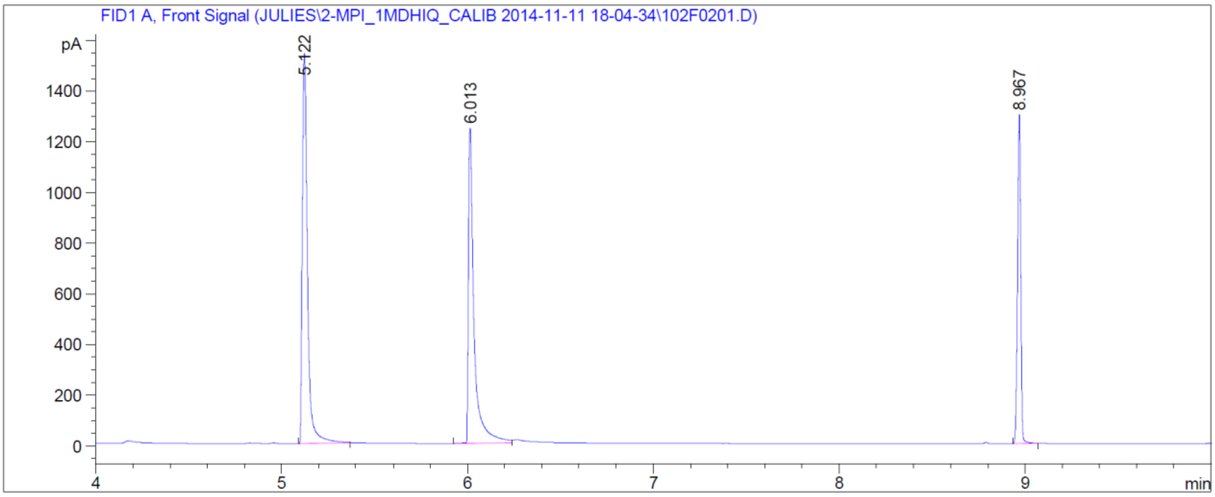

Supplementary Figure S20. Chromatographic separation of imine **1b** (t_r 6.0 min), amine **2b** (t_r 5.1 min), and *n*-dodecane (t_r 9.0 min) using GC method GC-A1.

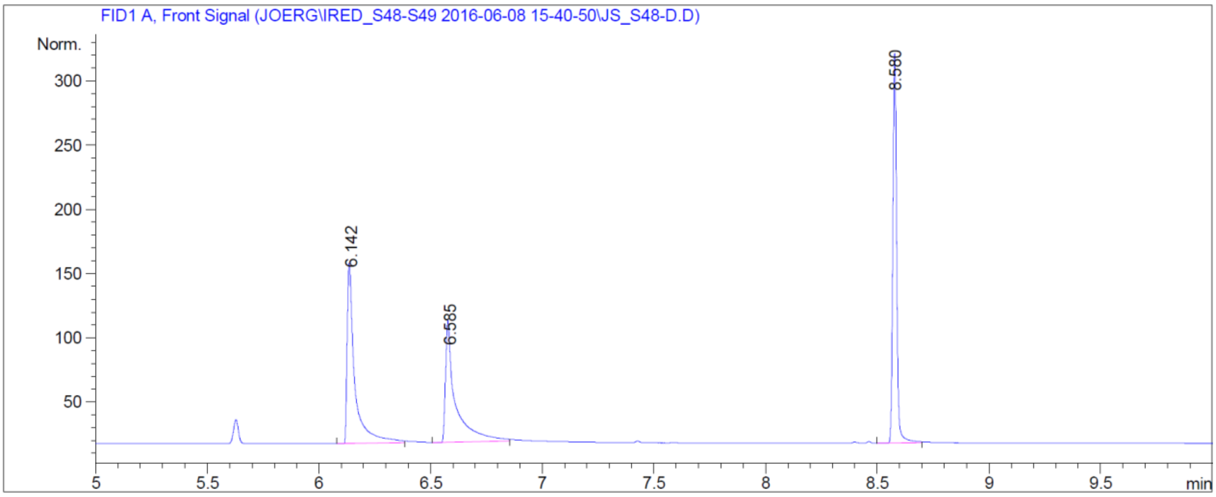

Supplementary Figure S21. Chromatographic separation of imine **1c** (t_r 6.6 min), amine **2c** (t_r 6.1 min), and *n*-dodecane (t_r 8.6 min) using GC method GC-A1.

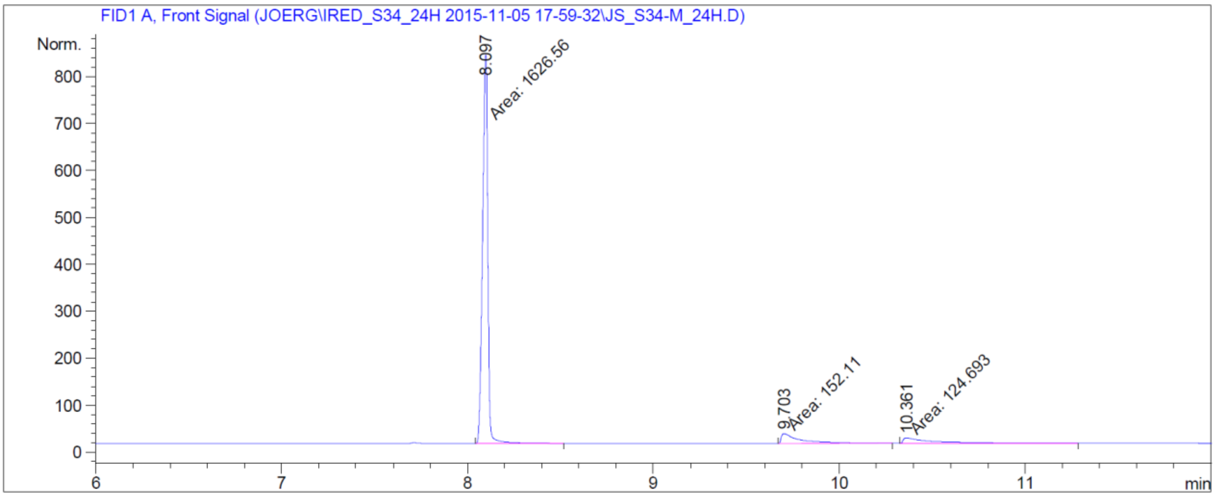

Supplementary Figure S22. Chromatographic separation of imine **1d** (t_r 10.4 min), amine **2d** (t_r 9.7 min), and *n*-dodecane (t_r 8.1 min) using GC method GC-A2.

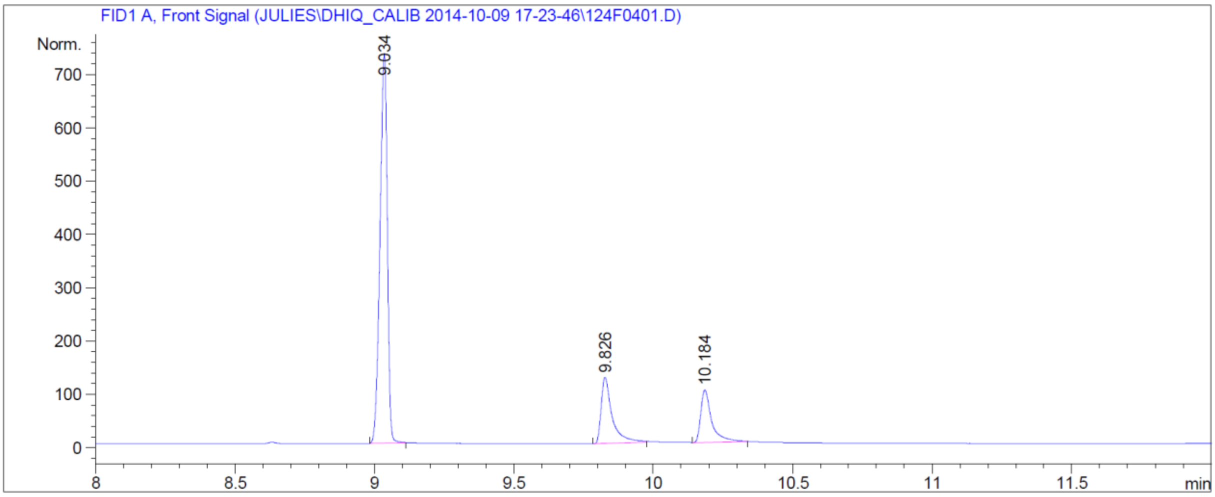

Supplementary Figure S23. Chromatographic separation of imine **1e** (t_r 9.8 min), amine **2e** (t_r 10.2 min), and *n*-dodecane (t_r 9.0 min) using GC method GC-A2.

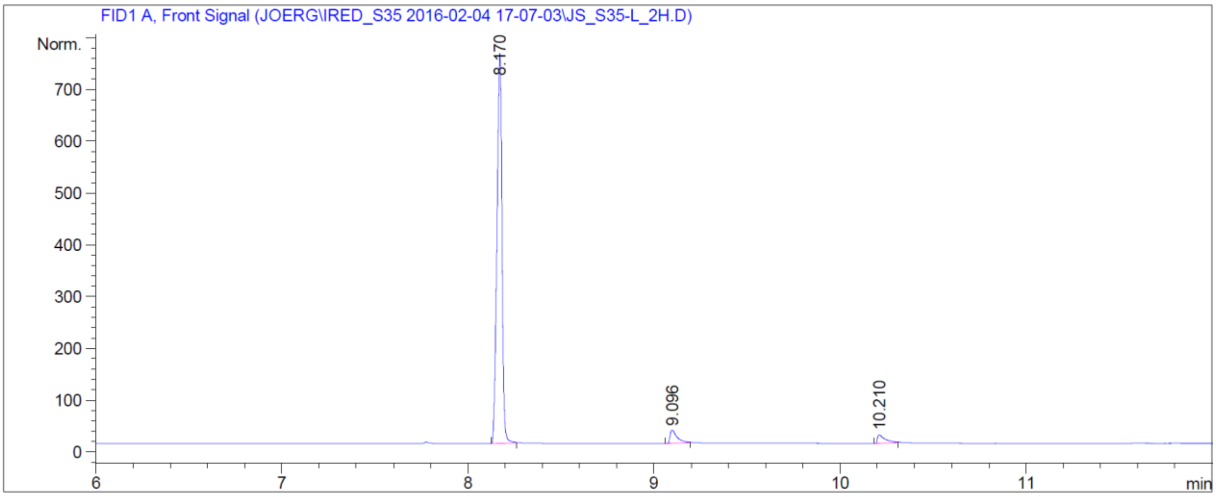

Supplementary Figure S24. Chromatographic separation of iminium ion **1f** (t_r 10.2 min), amine **2f** (t_r 9.1 min), and *n*-dodecane (t_r 8.2 min) using GC method GC-A2.

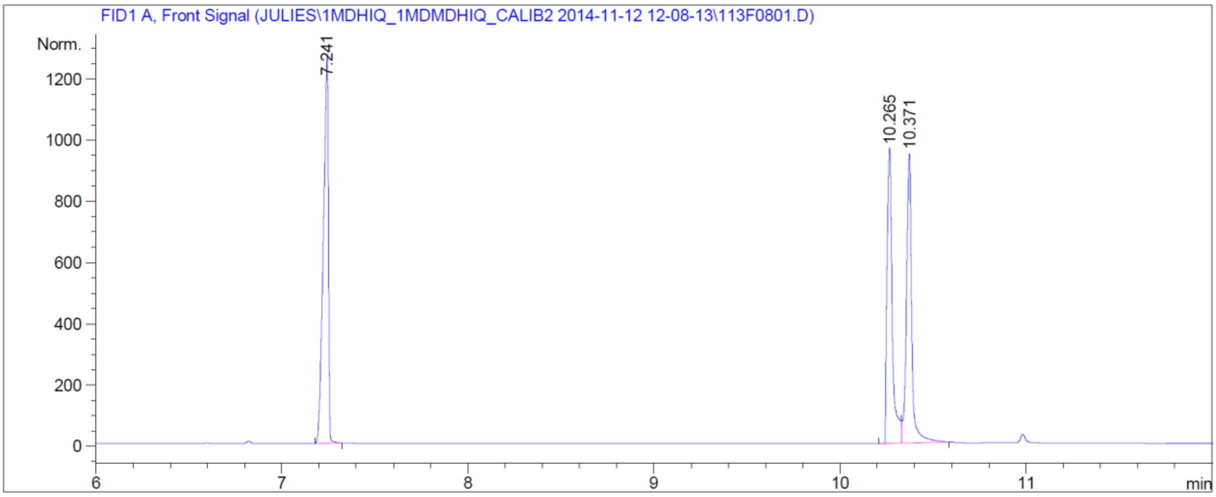

Supplementary Figure S25. Chromatographic separation of imine **1g** (t_r 10.4 min), amine **2g** (t_r 10.3 min), and *n*-dodecane (t_r 7.2 min) using GC method GC-A3.

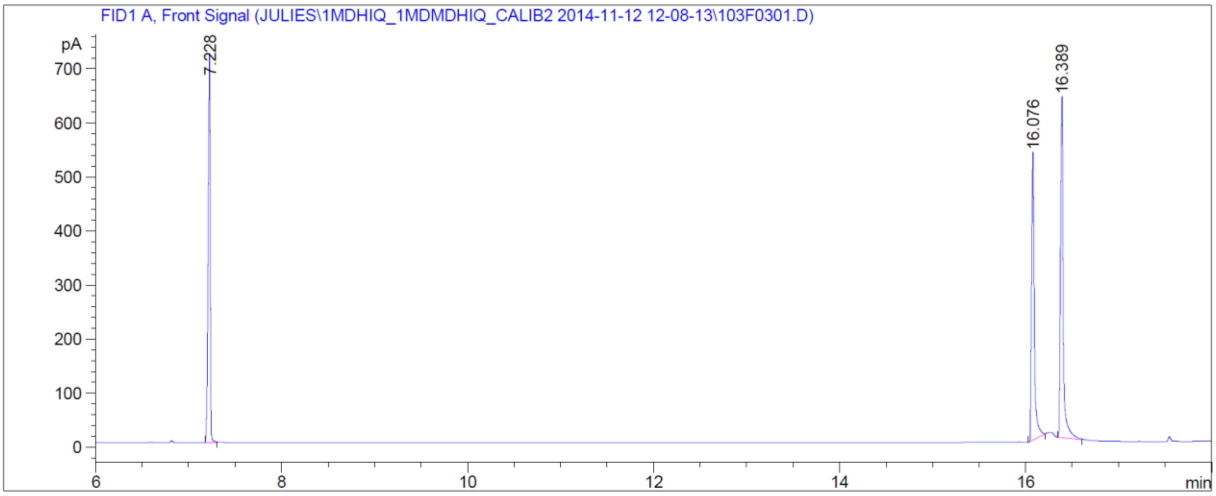

Supplementary Figure S26. Chromatographic separation of imine **1h** (t_r 16.4 min), amine **2h** (t_r 16.1 min), and *n*-dodecane (t_r 7.2 min) using GC method GC-A3.

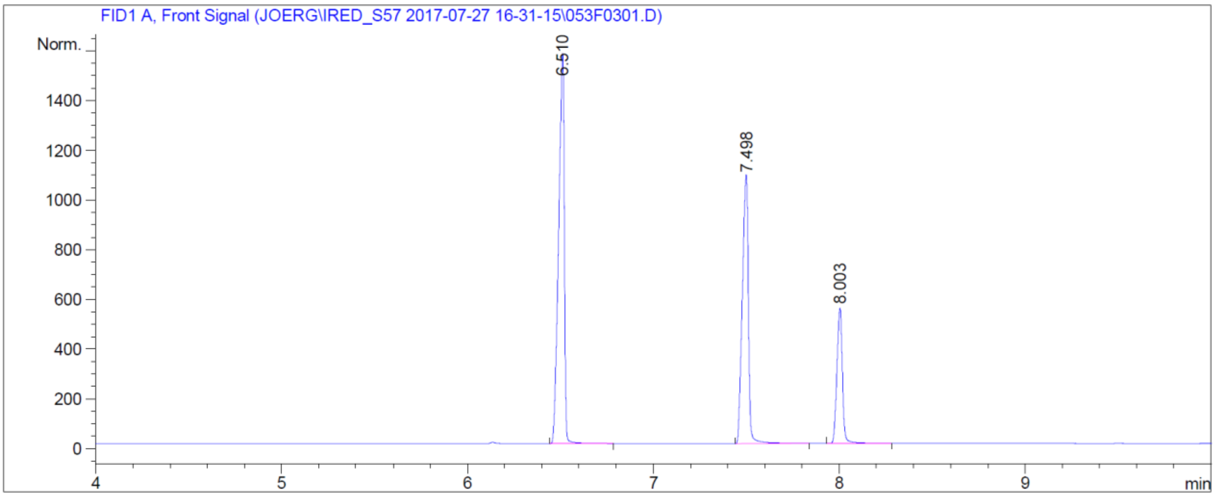

Supplementary Figure S27. Chromatographic separation of imine **1i** (t_r 7.5 min), amine **2i** (t_r 8.0 min), and *n*-dodecane (t_r 6.5 min) using GC method GC-A4.

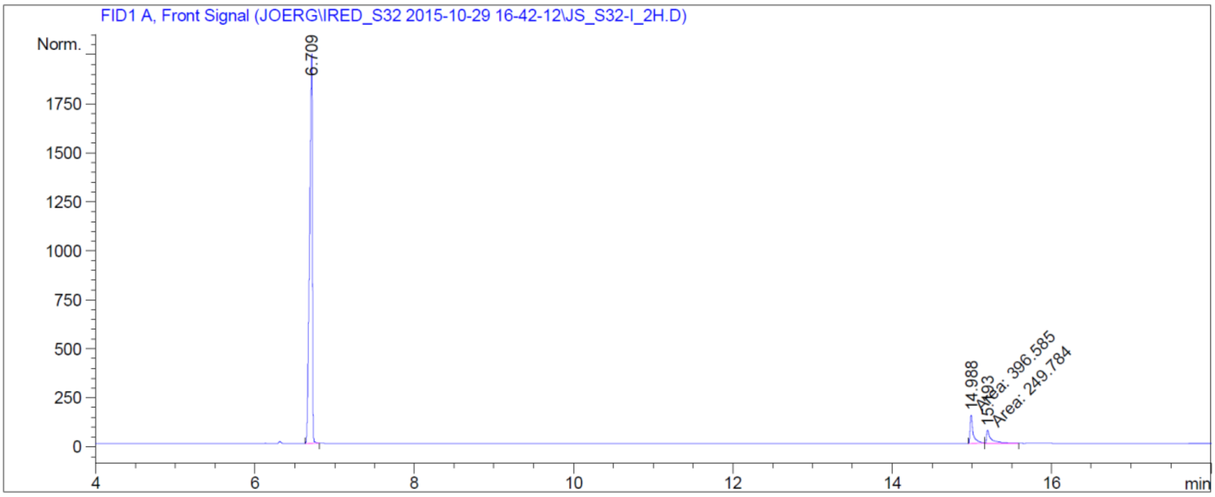

Supplementary Figure S28. Chromatographic separation of imine **1j** (t_r 15.2 min), amine **2j** (t_r 15.0 min), and *n*-dodecane (t_r 6.7 min) using GC method GC-A4.

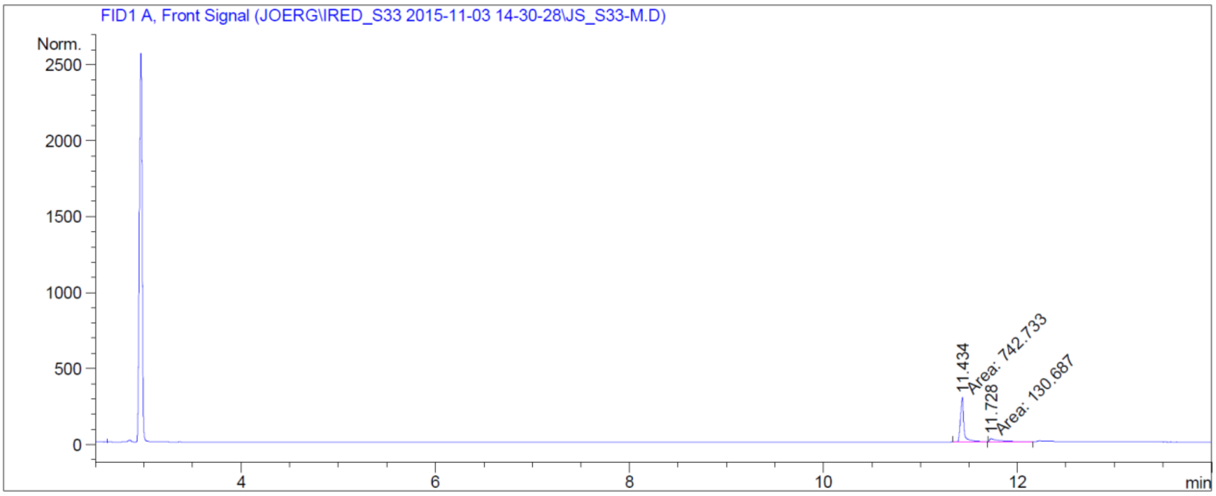

Supplementary Figure S29. Chromatographic separation of imine **1k** (t_r 11.7 min), amine **2k** (t_r 11.4 min), and *n*-dodecane (t_r 5.0 min) using GC method GC-A5.

Gas chromatography (chiral stationary phase):

Chiral-phase GC analyses were carried out on an *Agilent* 7890A GC system equipped with a flame-ionisation detector (FID) and either a *Macherey-Nagel* Hydrodex β-TBDAc capillary column (dimensions: 50 m × 0.25 mm × 0.25 μm; stationary phase: heptakis-(2,3-di-*O*-acetyl-6-*O*-*t*-butyldimethylsilyl)-β-cyclodextrin diluted with optimised polysiloxane) or a *RESTEK* Rt-βDEXse capillary column (dimensions: 30 m × 0.32 mm × 0.25 μm; stationary phase: 2,3-di-*O*-ethyl-6-*O*-*tert*-butyldimethylsilyl-β-cyclodextrin added into 14%-cyanopropylphenyl-methyl-polysiloxane), using hydrogen as carrier gas.

**Method GC-C1 (2a, 2b)**	
column:	Hydrodex β-TBDAc (50 m × 0.25 mm × 0.25 μm)
injector temperature:	230 °C
detector temperature:	250 °C
carrier gas flow rate:	1 mL/min
split ratio:	50:1
oven temperature program:	70 °C, 1 min; 20 °C/min to 150 °C; 150 °C, 10 min; 20 °C/min to 210 °C; 210 °C, 2 min
total run time:	20 min

**Method GC-C2 (2c)**	
column:	Rt-βDEXse (30 m × 0.32 mm × 0.25 μm)
injector temperature:	220 °C
detector temperature:	250 °C
carrier gas flow rate:	2 mL/min
split ratio:	90:1
oven temperature program:	80 °C, 1 min; 10 °C/min to 140 °C; 140 °C, 6 min; 10 °C/min to 200 °C; 200 °C, 1 min
total run time:	20 min

**Method GC-C3 (2d)**	
column:	Rt-βDEXse (30 m × 0.32 mm × 0.25 μm)
injector temperature:	220 °C
detector temperature:	250 °C
carrier gas flow rate:	2 mL/min
split ratio:	50:1
oven temperature program:	80 °C, 1 min; 20 °C/min to 130 °C; 130 °C, 9 min; 20 °C/min to 180 °C; 180 °C, 1 min
total run time:	20 min

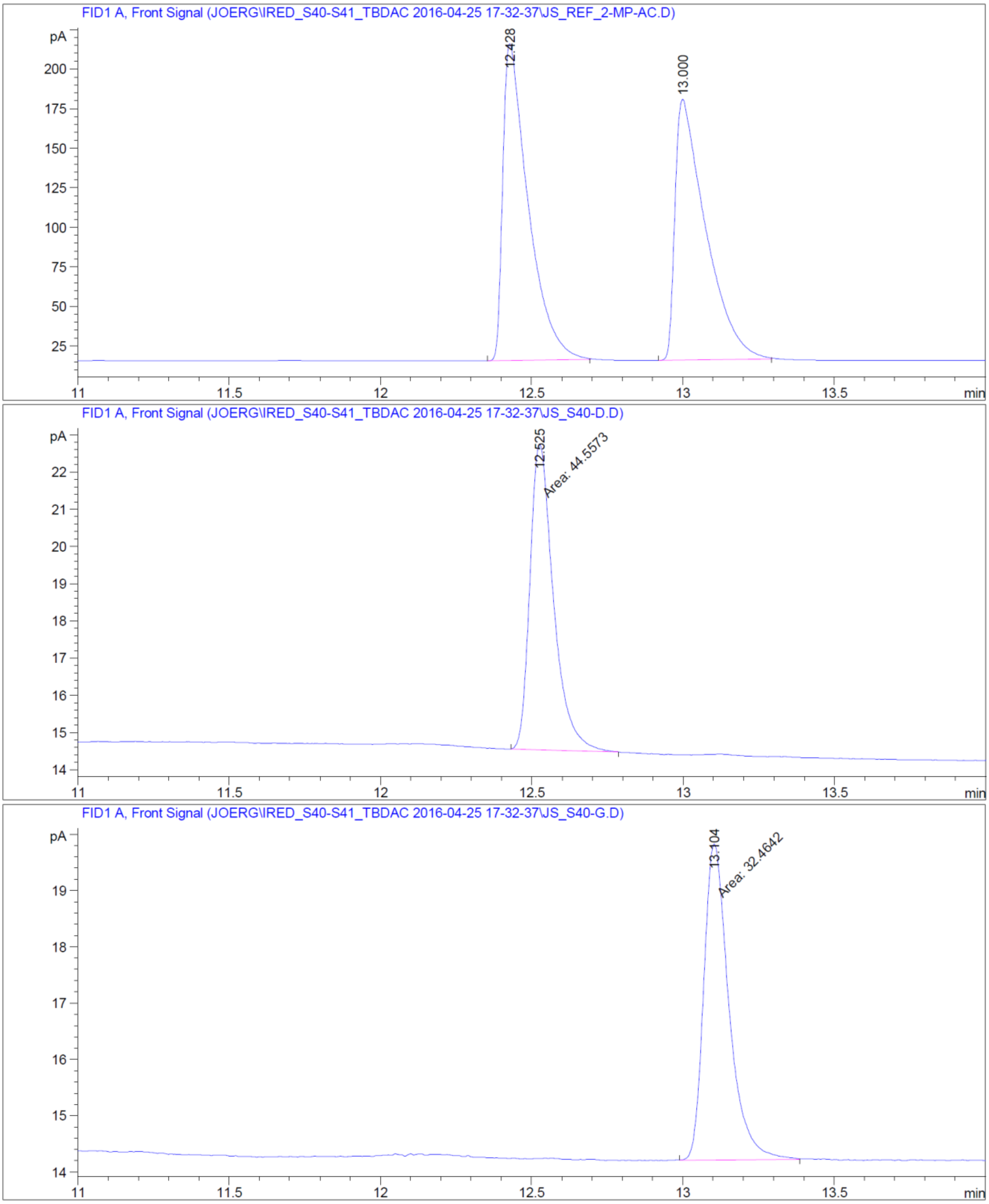

Supplementary Figure S30. Chromatographic separation of the enantiomers of amine **2a** using GC method GC-C1. Racemic standard (top), (*R*)-selective biotransformation using IRED-**D** (middle), (*S*)-selective biotransformation using IRED-**G** (bottom).

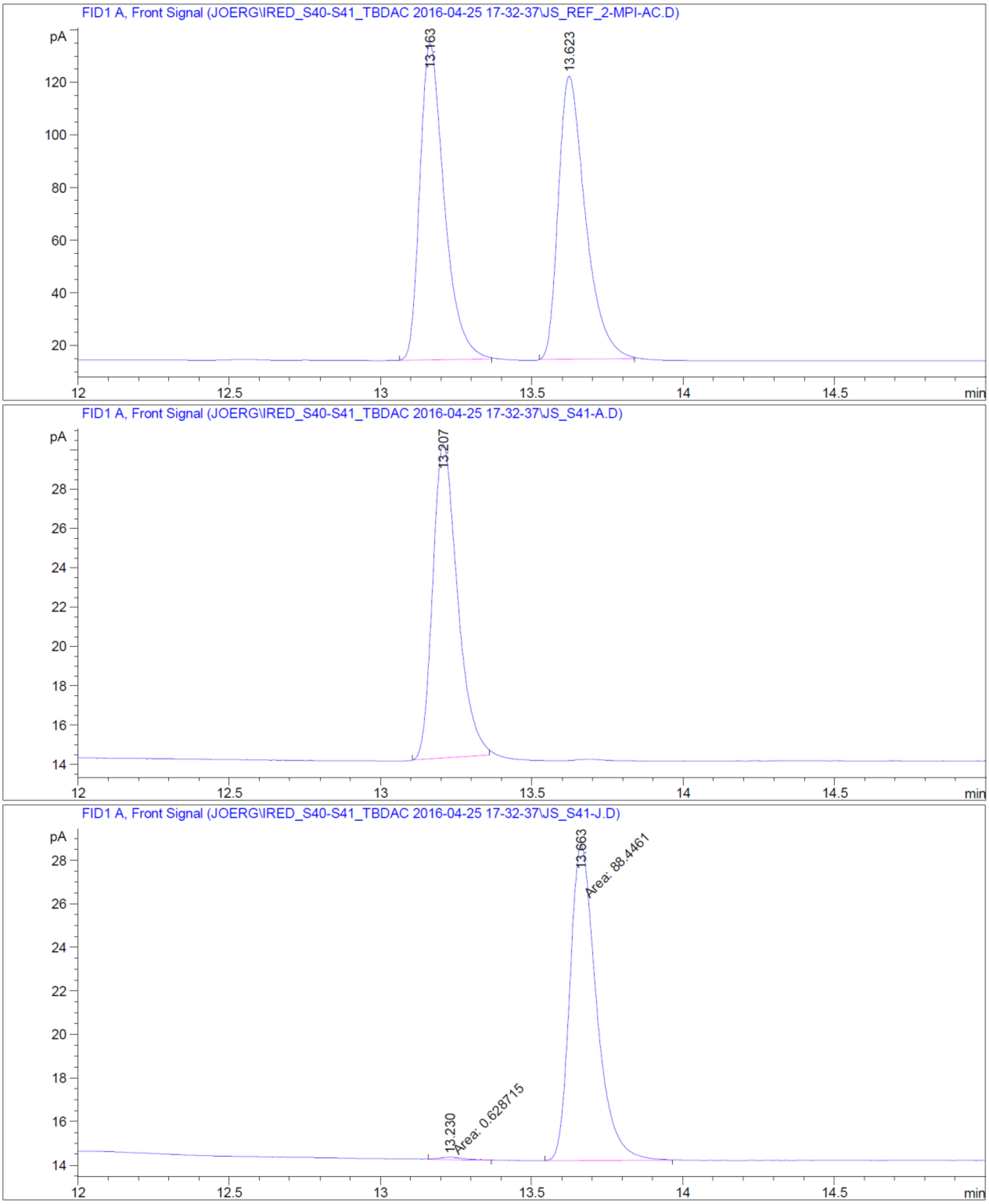

Supplementary Figure S31. Chromatographic separation of the enantiomers of amine **2b** using GC method GC-C1. Racemic standard (top), (*R*)-selective biotransformation using IRED-**A** (middle), (*S*)-selective biotransformation using IRED-**J** (bottom).

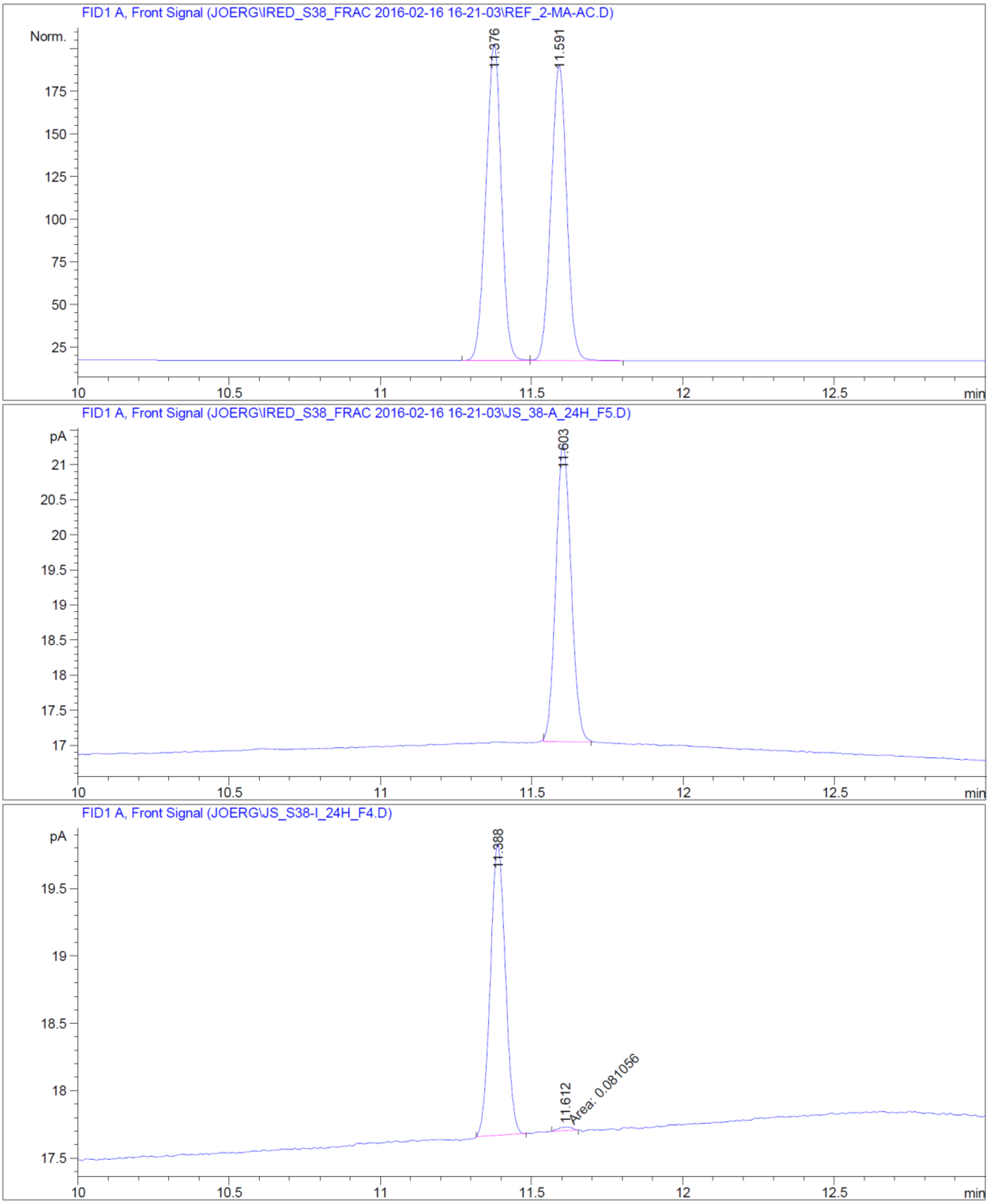

Supplementary Figure S32. Chromatographic separation of the enantiomers of amine **2c** using GC method GC-C2. Racemic standard (top), (*R*)-selective biotransformation using IRED-**A** (middle), (*S*)-selective biotransformation using IRED-**N** (bottom).

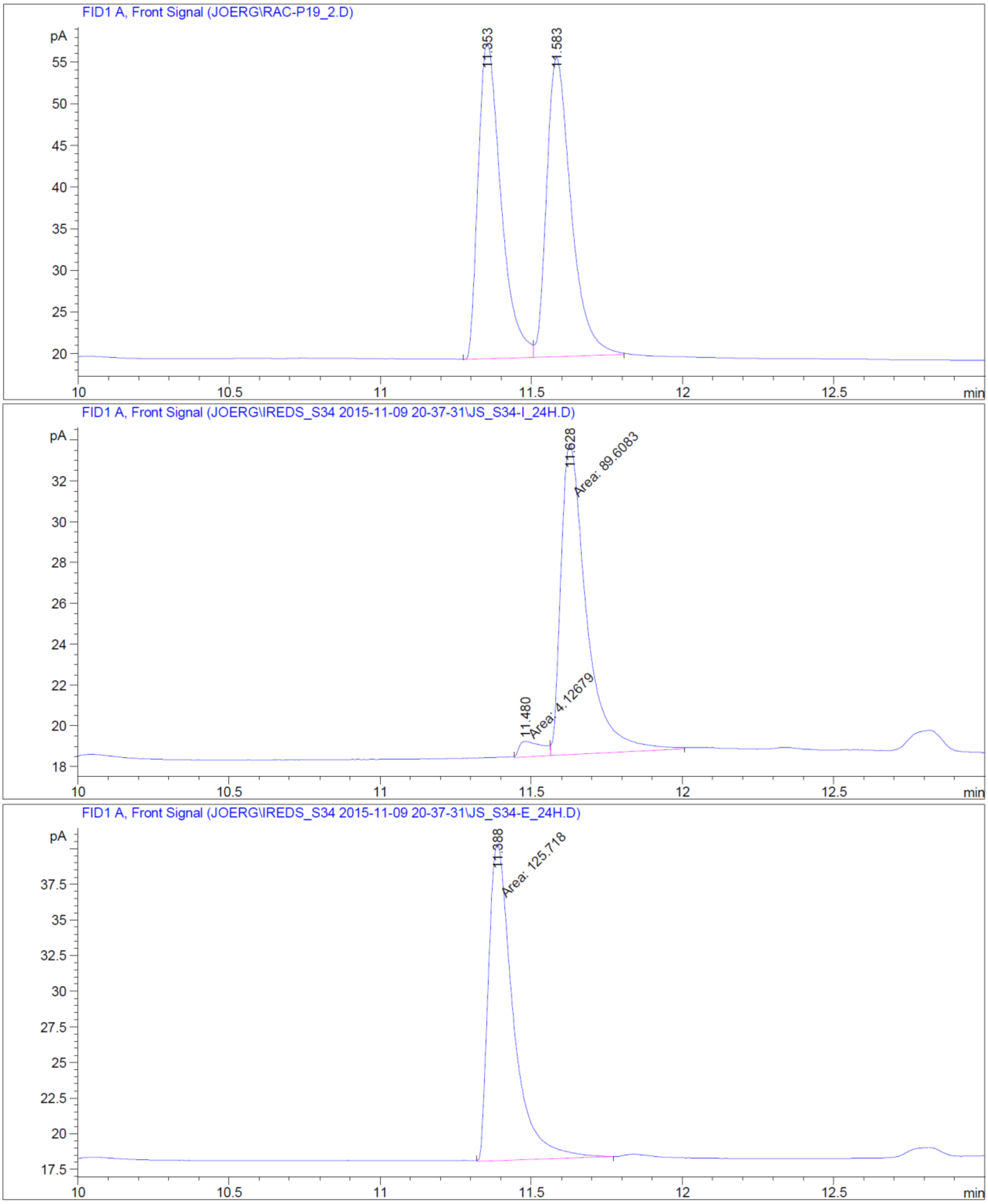

Supplementary Figure S33. Chromatographic separation of the enantiomers of amine **2c** using GC method GC-C3. Racemic standard (top), (*R*)-selective biotransformation using IRED-**I** (middle), (*S*)-selective biotransformation using IRED-**E** (bottom).

High-performance liquid chromatography (chiral stationary phase):
Chiral-phase HPLC analyses were carried out on a *Shimadzu* HPLC system (Communication Bus Module CBM-20 A, Column Oven CTO-20 AC, Degasser DGU-20 A5, Liquid Chromatograph LC-20 AD, Auto sampler SIL-20 AC, Diode Array Detector SPD-M20 A) equipped with either a *Daicel* Chiralcel OD-H column (dimensions: 250 mm × 4.6 mm; stationary phase: coated cellulose-tris-(3,5-dimethylphenylcarbamate); particle size 5 μm) or a *Daicel* Chiralpak IC column (dimensions: 250 mm × 4.6 mm; stationary phase: immobilised cellulose-tris-(3,5-dichloro-phenylcarbamate), using *n*-heptane and 2-propanol, each containing 0.1% (v/v) diethylamine, as eluents.

**Method HPLC-C1 (2g)**	
column:	Chiralcel OD-H
column temperature:	30 °C
eluent:	*n*-heptane : 2-propanol = 99:1 (+ 0.1% DEA)
eluent flow rate:	0.5 mL/min
run time:	40 min
integration wavelengths:	265 nm, 272 nm

**Method-HPLC-C2 (2h)**	
column:	Chiralcel OD-H
column temperature:	30 °C
eluent:	*n*-heptane : 2-propanol = 90:10 (+ 0.1% DEA)
eluent flow rate:	1 mL/min
run time:	40 min
integration wavelengths:	215 nm, 282 nm

**Method HPLC-C3 (2i)**	
column:	Chiralcel OD-H
column temperature:	30 °C
eluent:	*n*-heptane : 2-propanol = 98:2 (+ 0.1% DEA)
eluent flow rate:	0.5 mL/min
run time:	40 min
integration wavelengths:	242 nm, 294 nm

**Method-HPLC-C4 (2j)**	
column:	Chiralcel OD-H
column temperature:	30 °C
eluent:	*n*-heptane : 2-propanol = 80:20 (+ 0.1% DEA)
eluent flow rate:	1 mL/min
run time:	40 min
integration wavelengths:	231 nm, 271 nm

**Method HPLC-C5 (2k)**	
column:	Chiralpak IC
column temperature:	30 °C
eluent:	*n*-heptane : 2-propanol = 95:5 (+ 0.1% DEA)
eluent flow rate:	1 mL/min
run time:	40 min
integration wavelengths:	225 nm, 297 nm

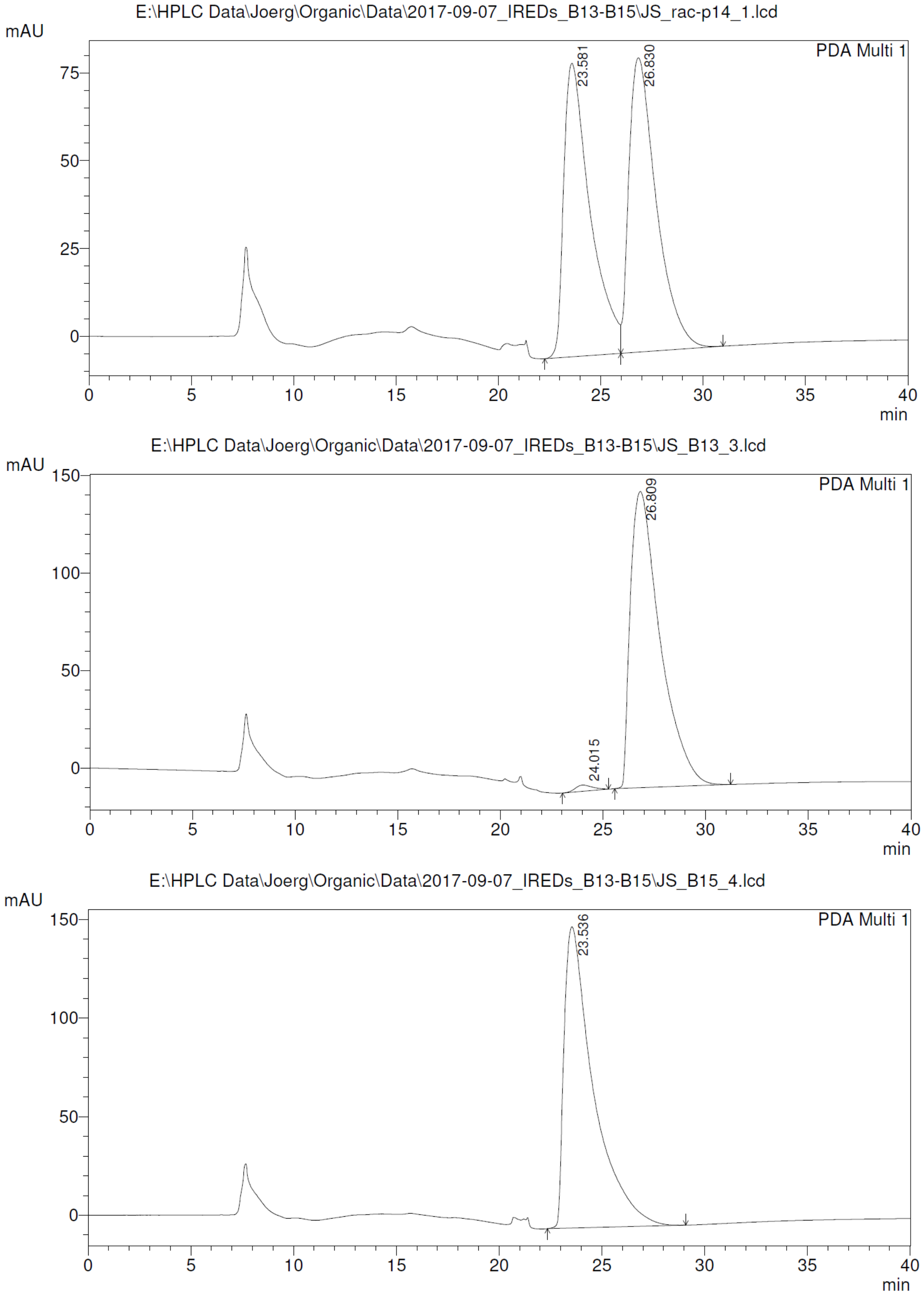

Supplementary Figure S34. Chromatographic separation of the enantiomers of amine **2g** using HPLC method HPLC-C1. Racemic standard (top), (*R*)-selective biotransformation using IRED-**D** (middle), (*S*)-selective biotransformation using IRED-**J** (bottom).

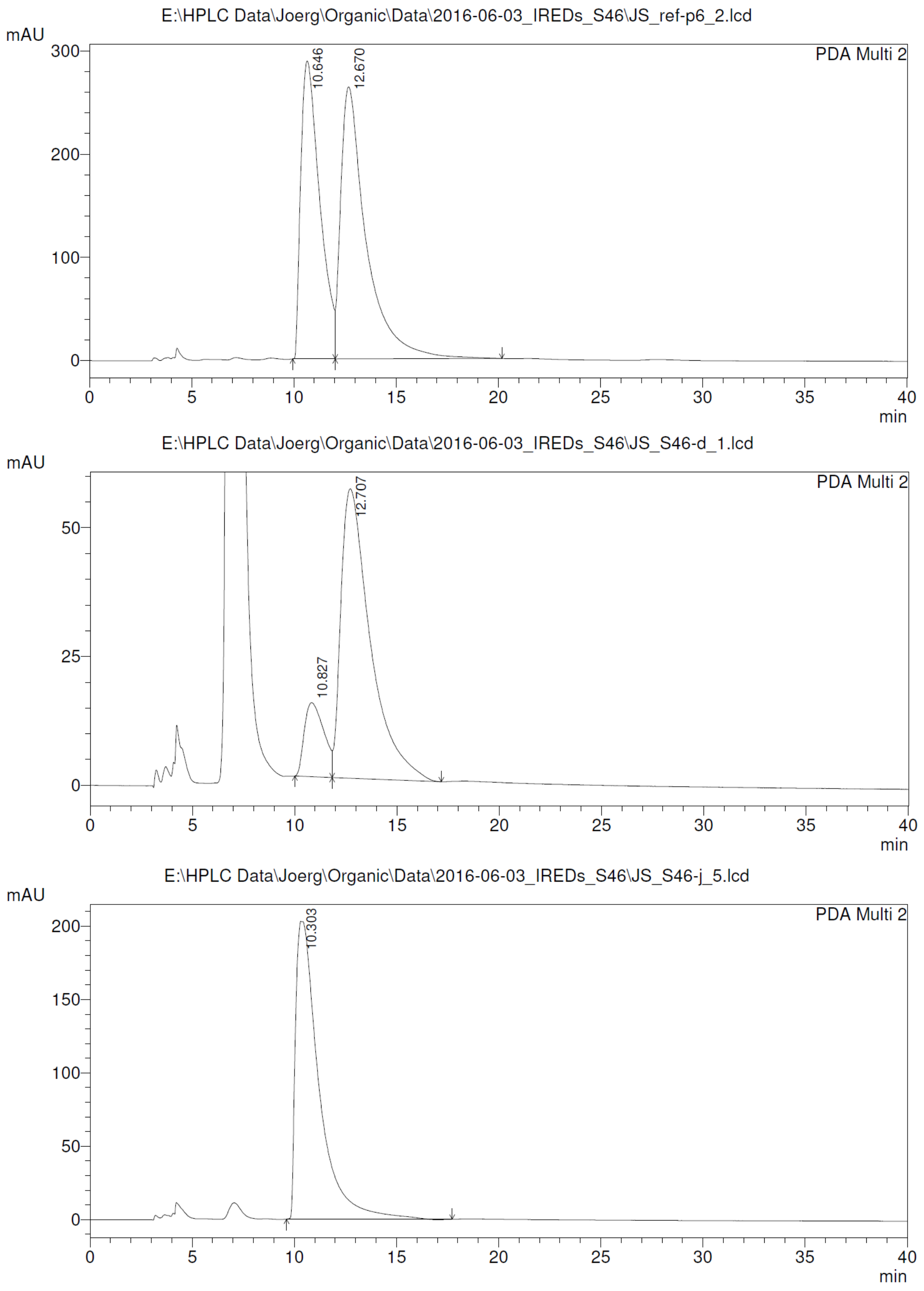

Supplementary Figure S35. Chromatographic separation of the enantiomers of amine **2h** using HPLC method HPLC-C2. Racemic standard (top), (*R*)-selective biotransformation using IRED-**D** (middle), (*S*)-selective biotransformation using IRED-**J** (bottom).

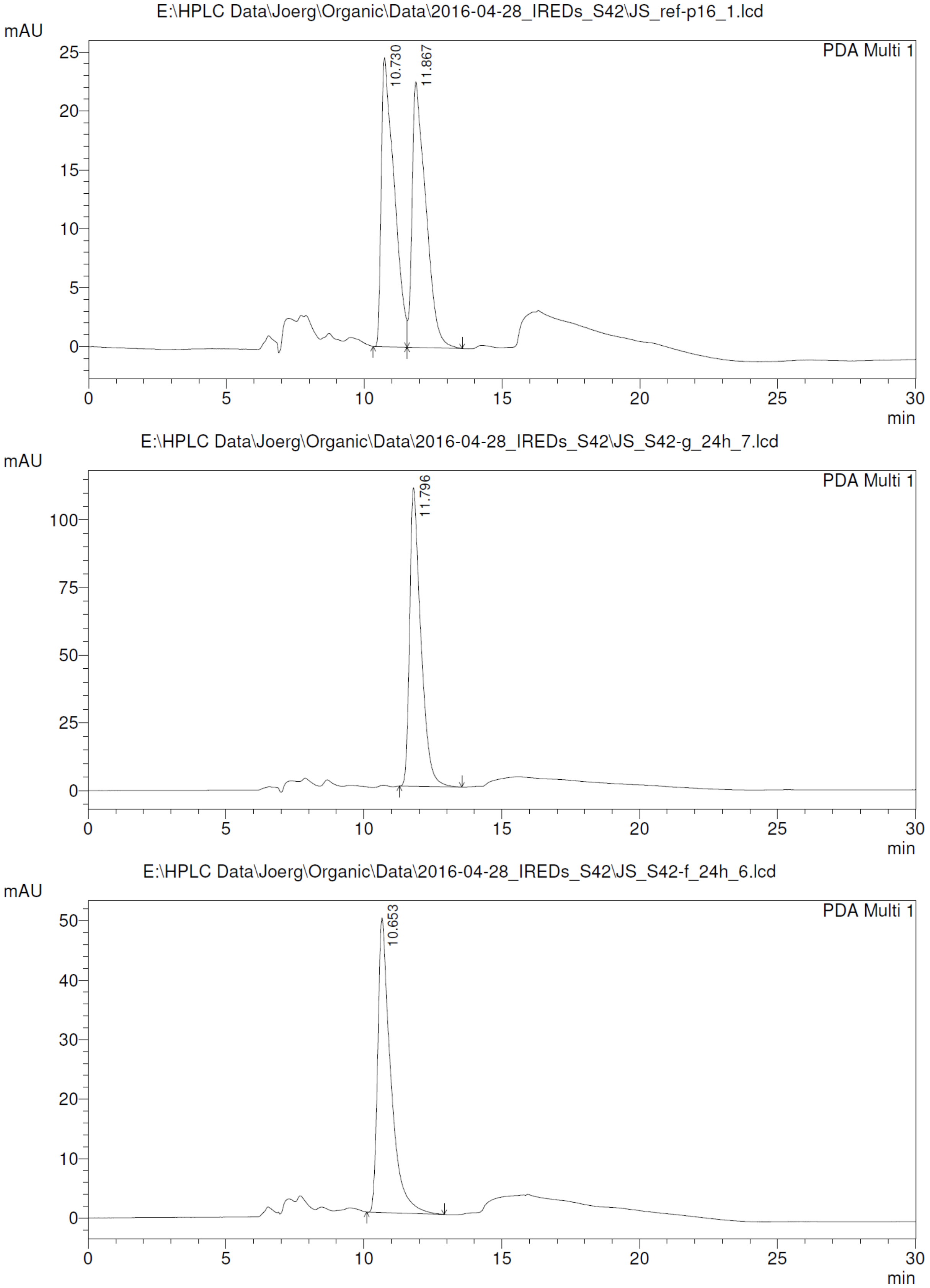

Supplementary Figure S36. Chromatographic separation of the enantiomers of amine **2i** using HPLC method HPLC-C3. Racemic standard (top), (*R*)-selective biotransformation using IRED-**G** (middle), (*S*)-selective biotransformation using IRED-**F** (bottom).

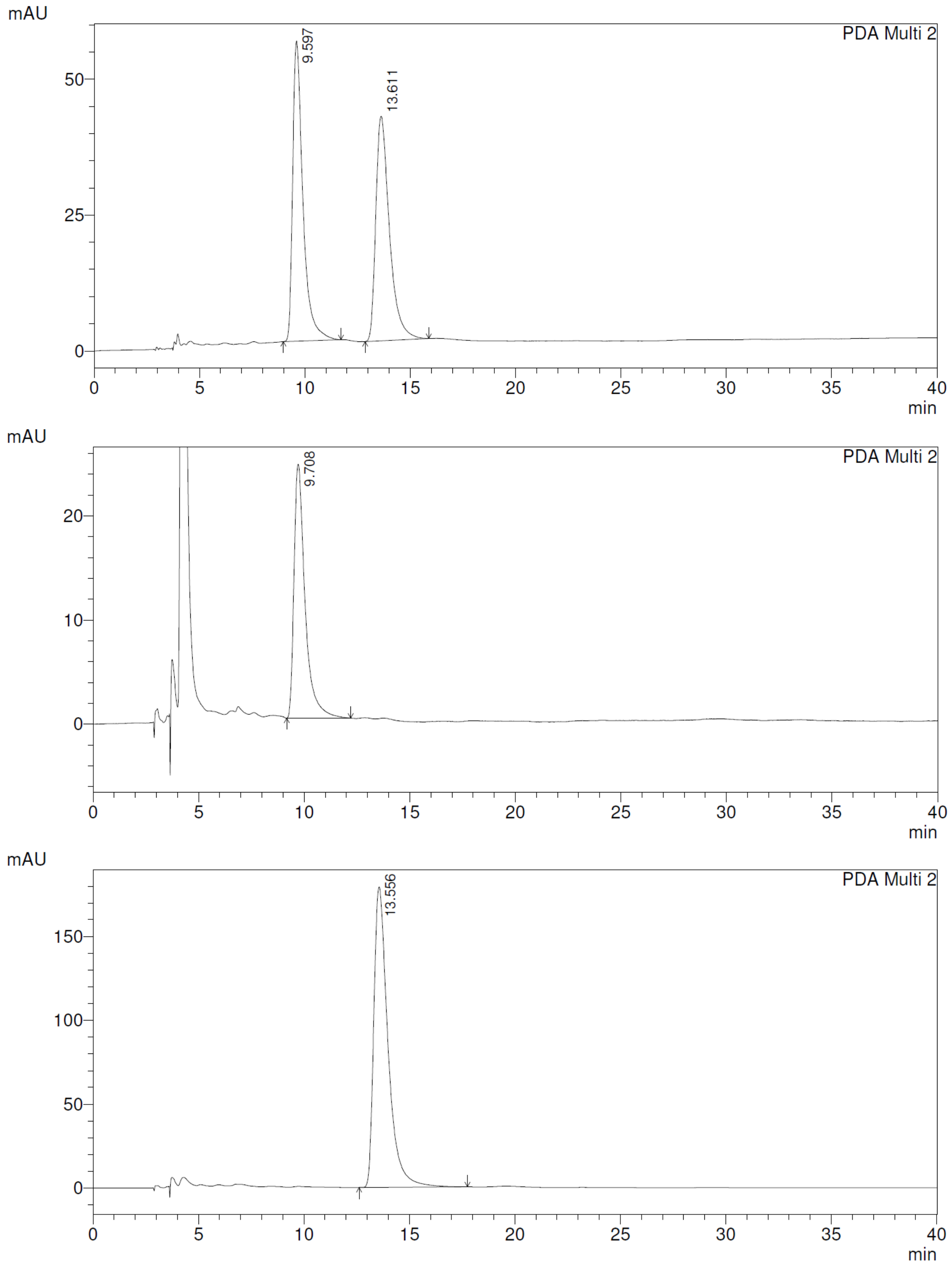

Supplementary Figure S37. Chromatographic separation of the enantiomers of amine **2j** using HPLC method HPLC-C4. Racemic standard (top), (*R*)-selective biotransformation using IRED-**C** (middle), (*S*)-selective biotransformation using IRED-**I** (bottom).

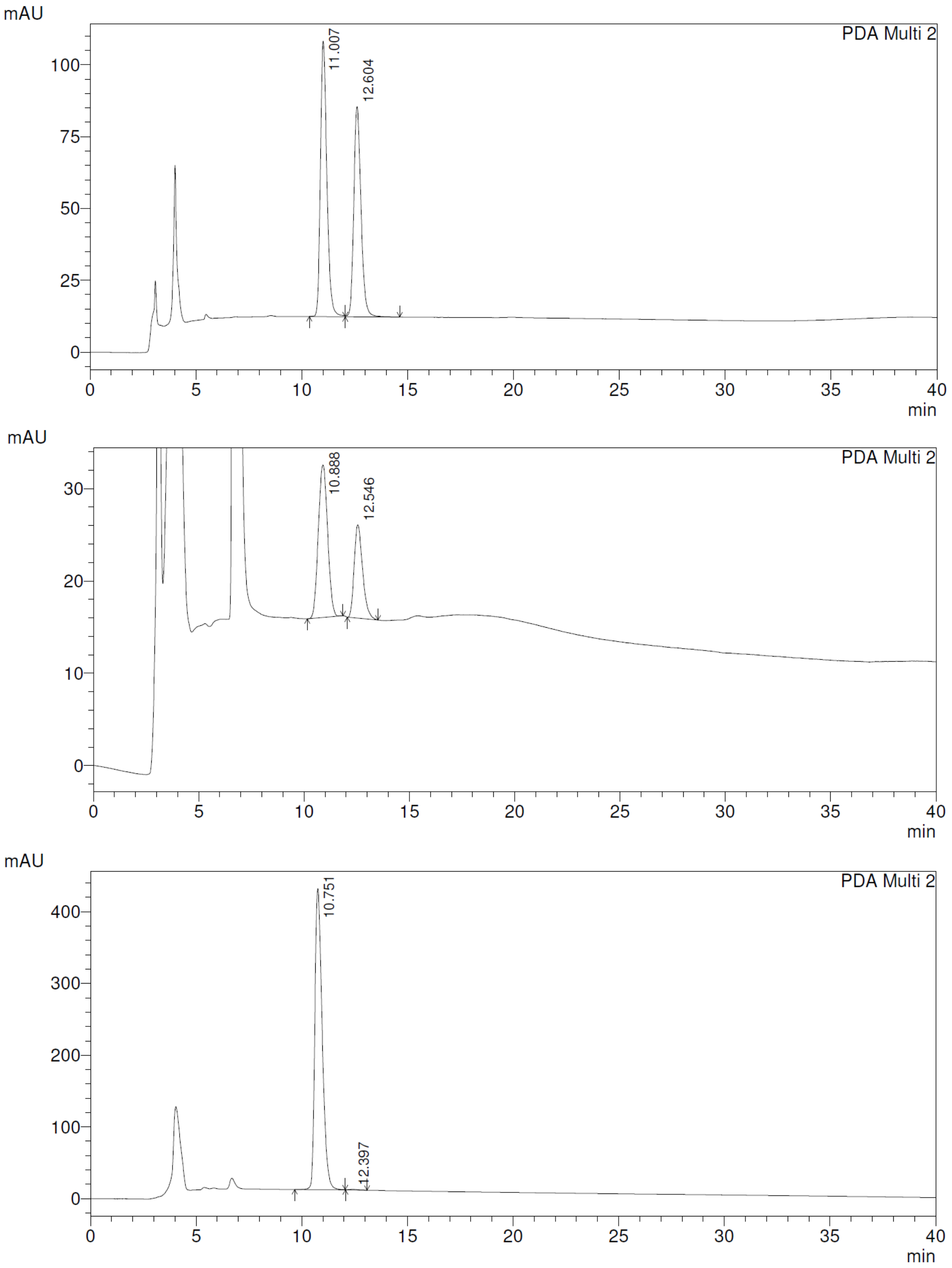

Supplementary Figure S38. Chromatographic separation of the enantiomers of amine **2k** using HPLC method HPLC-C5. Racemic standard (top), poorly (*S*)-selective biotransformation using IRED-**D** (middle), (*S*)-selective biotransformation using IRED-**K** (bottom).

Determination of absolute configurations:

The absolute configurations of amines **2a–d** and **2g–k** were determined by one or more of the following methods: (*i*) Comparison of GC or HPLC retention time with a commercially available, enantiomerically pure reference compound, (*ii*) comparison of GC or HPLC retention time with an IRED product of literature-known configuration, (*iii*) comparison of GC or HPLC elution order of enantiomers with the elution order reported in the literature for identical chromatographic conditions, (*iv*) comparison of the optical rotation of the isolated compound with values reported in the literature. Supplementary Table S6 shows the methods used for each chiral amine and lists the literature references used, if applicable.

Method	2a	2b	2c	2d	2g	2h	2i	2j	2k
Reference compound	×								
Known IRED product	4, 9-11	4, 10-11	4, 11	4, 11	4, 10-11	4		4	4
Elution order					12	4	13		
Optical rotation					14				

Supplementary Table S6. Summary of methods used for the determination of absolute configurations, including literature references.

DNA and Protein Sequences

NOTE: In the DNA sequences, start codons are underlined, stop codons are typeset in lowercase letters, restrictions sites are highlighted by a light blue box, and the 'stuffer DNA' appended at the 5' and 3' ends of the gene (required for the restriction digest) is typeset in grey. In the protein sequences, the N-terminal methionine residue of the native sequence is underlined and the N-terminal elongation containing the His-tag is typeset in grey.

IRED-A (M4ZRJ3)

Native Gene Sequence (Genbank ID AB747176.1):

ATGGGCGACAACCGCACCCCGGTGACGGTCATCGGCCTCGGCCTCATGGGACAGGCGCTCGCCGCCGC
GTTCCTCGAGGCCGGCCACACCACGACCGTGTGGAACCGTTCGGCGGGCAAGGCCGAGCAGCTCGTCT
CGCAGGGCGCGGTGCAGGCCGCCACGCCCGCCGACGCGGTGGCCGCCAGTGAGCTGGTCGTGGTCTGC
CTGTCGACGTACGACAACATGCACGACGTGATCGGTTCGCTCGGTGAGTCCCTCCGGGGCAAGGTCAT
CGTGAACCTGACGTCCGGCTCGTCGGACCAGGGCCGCGAGACGGCGGCGTGGGCCGAGAAGCAGGGCG
TGGAGTACCTCGACGGCGCGATCATGATCACGCCGCCCGGCATCGGCACCGAGACCGCCGTCCTCTTC
TACGCCGGTACGCAGAGCGTGTTCGAGAAGTACGAGCCCGCGCTCAAGCTGCTCGGCGGCGGCACCAC
GTACCTCGGCACCGACCACGGTATGCCCGCGCTGTACGACGTGTCGCTGCTCGGCCTCATGTGGGGCA
CGCTCAACAGCTTCCTGCACGGTGTCGCCGTGGTGGAGACCGCGGGCGTCGGCGCCCAGCAGTTCCTG
CCGTGGGCGCACATGTGGCTGGAGGCGATCAAGATGTTCACCGCCGACTACGCGGCCCAGATCGACGC
CGGTGACGGCAAGTTCCCCGCCAACGACGCCACGCTGGAGACGCACCTGGCGGCGCTCAAGCACCTGG
TGCACGAGAGCGAGGCGCTGGGTATCGACGCCGAACTGCCGAAGTACTCCGAGGCGTTGATGGAGCGC
GTCATCAGCCAGGGTCACGCCAAGAACAGCTACGCGGCCGTGCTCAAGGCGTTCCGCAAGCCGTCGGA
Gtga [888 bp]

Optimised Gene Sequence incl. 'Stuffer DNA' (*GeneArt*):

GTGCCGCGCGGCAGCCATATGGGTGATAATCGTACACCGGTTACCGTTATTGGTCTGGGTCTGATGGG
TCAGGCACTGGCAGCAGCATTTCTGGAAGCAGGTCATACCACCACCGTTTGGAATCGTAGCGCAGGTA
AAGCAGAACAGCTGGTTAGCCAGGGTGCAGTTCAGGCAGCAACACCGGCAGATGCAGTTGCAGCAAGC
GAACTGGTTGTTGTTTGTCTGAGCACCTATGATAATATGCATGATGTTATTGGTAGCCTGGGTGAAAG
CCTGCGTGGTAAAGTTATTGTTAATCTGACCAGCGGTAGCAGCGATCAGGGTCGTGAAACCGCAGCAT
GGGCAGAAAAACAGGGTGTTGAATATCTGGATGGTGCCATTATGATTACCCCTCCGGGTATTGGCACC
GAAACAGCAGTTCTGTTTTATGCAGGCACCCAGAGCGTTTTTGAAAAATATGAACCGGCACTGAAACT
GCTGGGTGGTGGCACCACATATCTGGGCACCGATCATGGTATGCCTGCACTGTATGATGTTAGCCTGC
TGGGCCTGATGTGGGGCACCCTGAATAGCTTTCTGCATGGTGTTGCAGTTGTTGAAACAGCGGGTGTT
GGTGCACAGCAATTTCTGCCGTGGGCACACATGTGGCTGGAAGCCATTAAAATGTTTACCGCAGATTA
TGCAGCACAGATTGATGCCGGTGATGGTAAATTTCCGGCAAATGATGCAACCCTGGAAACCCATCTGG
CAGCCCTGAAACATCTGGTTCATGAAAGCGAAGCACTGGGTATTGATGCAGAACTGCCGAAATATTCT
GAAGCACTGATGGAACGTGTTATTAGTCAGGGTCATGCCAAAAATAGCTATGCAGCCGTTCTGAAAGC
ATTTCGTAAACCGAGCGAAtaaCTCGAGCCACTGAGATCCGGC [927 bp]

Protein Sequence incl. N-terminal His-tag:

MGSSHHHHHHSSGLVPRGSHMGDNRTPVTVIGLGLMGQALAAAFLEAGHTTTVWNRSAGKAEQLVSQG
AVQAATPADAVAASELVVVCLSTYDNMHDVIGSLGESLRGKVIVNLTSGSSDQGRETAAWAEKQGVEY
LDGAIMITPPGIGTETAVLFYAGTQSVFEKYEPALKLLGGGTTYLGTDHGMPALYDVSLLGLMWGTLN
SFLHGVAVVETAGVGAQQFLPWAHMWLEAIKMFTADYAAQIDAGDGKFPANDATLETHLAALKHLVHE
SEALGIDAELPKYSEALMERVISQGHAKNSYAAVLKAFRKPSE [315 aa]

Predicted Properties (*ExPASy* ProtParam):

Isoelectric point: 5.59
Molecular weight: 33,270 Da
Absorbance at 280 nm: 40,910 M^{-1} cm^{-1}

IRED-B (Q1EQE0)

Native Gene Sequence (Genbank ID AB254080.1):

ATGCCCGACAACCCCTCTACGAAAGGGCGCATGATGCGGAACCAGCAGGCAGAGCACACCCCTGTGAC
GGTCATCGGCCTCGGCCTCATGGGCCAGGCCCTCGCCGGGGCGTTCCTGGGCGCGGACACCCCACGA
CCGTGTGGAACCGGACGGCGGCGAAGGCCGAACCGCTCGTCGCCCGGGGCGCGAAGAGCGCGGGATCG
GTCGCCGAAGCGGTCGCCGCGAGCCCGCTGGTGGTCGTCTGCGTCTCGGACTACGACGCCGTGCACGC
GCTCCTCGACCCGCTCGACGGCACGGCCCTCCAGGGGCGCACCCTGGTGAACCTGACCTCCGGCACCT
CGGCCCAGGCCCGCGAGCGCGCCGCGTGGGCGGACGGCCGCGGCGCCGACTACCTCGACGGCGCGATC
CTGGCGGGACCCGCCGCGATCGGCACGGCGGACGCCGTCGTCCTCCTCAGCGGGCCCCGGTCGGCCTT
CGACCCGCACGCGTCTGCCCTCGGCGGCCTCGGCGCGGGAACGACGTACCTCGGCGCCGACCACGGTC
TCGCGTCGCTGTACGACGCGGCGGGCCTCGTCATGATGTGGAGCATCCTGAACGGCTTCCTGCAAGGC
GCCGCGCTGCTCGGCACGGCCGGTGTGGACGCCACGACGTTCGCCCCGTTCATCACCCAGGGAATAGG
AACCGTCGCCGACTGGTTGCCCGGCTACGCGCGACAGATCGACGACGGCGCGTACCCCGCCGACGACG
CCGCCATCGACACCCATCTGGCCACCATGGAACACCTCATCCACGAGAGCGAGTTCCTCGGAGTGAAC
GCCGAACTCCCCAGGTTCATCAAGGCCCTCGCCGACCGGGCCGTGGCCGACGGGCACGGCGGCAGCGG
CTACCCGGCGCTGATCGAACAGTTCCGCACGCACTCAGGGAAGtga [930 bp]

Optimised Gene Sequence incl. 'Stuffer DNA' (*GeneArt*):

GTGCCGCGCGGCAGCCATATGCCGGATAATCCGAGCACCAAAGGTCGTATGATGCGTAATCAGCAGGC
AGAACATACACCGGTTACCGTTATTGGTCTGGGTCTGATGGGTCAGGCACTGGCAGGCGCATTTCTGG
GTGCAGGTCATCCGACCACCGTTTGGAATCGTACCGCAGCAAAAGCAGAACCGCTGGTTGCACGTGGT
GCAAAAAGCGCAGGTAGCGTTGCAGAAGCAGTTGCAGCAAGCCCTCTGGTTGTTGTTTGTGTTAGCGA
TTATGATGCAGTTCATGCACTGCTGGATCCGCTGGATGGCACCGCACTGCAGGGTCGTACCCTGGTTA
ATCTGACCAGCGGCACCAGCGCACAGGCACGTGAACGTGCAGCATGGGCAGATGGTCGTGGTGCAGAT
TATCTGGATGGTGCAATTCTGGCAGGTCCGGCAGCAATTGGTACAGCAGATGCAGTTGTTCTGCTGAG
CGGTCCGCGTAGCGCATTTGATCCGCATGCAAGCGCACTGGGTGGTCTGGGAGCAGGCACCACCTATC
TGGGTGCCGATCATGGTCTGGCAAGCCTGTATGATGCCGCAGGTCTGGTTATGATGTGGTCAATTCTG
AATGGTTTTCTGCAGGGTGCTGCCCTGCTGGGTACAGCCGGTGTTGATGCAACCACCTTTGCACCGTT
TATTACCCAGGGTATTGGCACCGTTGCAGATTGGCTGCCTGGTTATGCACGTCAGATTGATGATGGTG
CATATCCGGCAGATGATGCAGCCATTGATACCCATCTGGCAACCATGGAACATCTGATTCATGAAAGC
GAATTTCTGGGCGTTAATGCAGAACTGCCTCGTTTTATCAAAGCACTGGCCGATCGTGCAGTTGCCGA
TGGTCATGGTGGTAGCGGCTATCCGGCACTGATTGAACAGTTTCGTACCCATAGCGGTAAAtaaCTCG
AGCCACTGAGATCCGGC [969 bp]

Protein Sequence incl. N-terminal His-tag:

MGSSHHHHHHSSGLVPRGSHMPDNPSTKGRMMRNQQAEHTPVTVIGLGLMGQALAGAFLGAGHPTTVW
NRTAAKAEPLVARGAKSAGSVAEAVAASPLVVVCVSDYDAVHALLDPLDGTALQGRTLVNLTSGTSAQ
ARERAAWADGRGADYLDGAILAGPAAIGTADAVVLLSGPRSAFDPHASALGGLGAGTTYLGADHGLAS
LYDAAGLVMMWSILNGFLQGAALLGTAGVDATTFAPFITQGIGTVADWLPGYARQIDDGAYPADDAAI
DTHLATMEHLIHESEFLGVNAELPRFIKALADRAVADGHGGSGYPALIEQFRTHSGK [329 aa]

Predicted Properties (*ExPASy* ProtParam):

Isoelectric point:	5.79
Molecular weight:	33,681 Da
Absorbance at 280 nm:	32,430 M^{-1} cm^{-1}

IRED-C (W7VJL8)

Native Gene Sequence (Genbank ID KK037233.1):

ATGGCACCCGACACTGTGGAGAAGACGCCCGTGACGCTGCTGGGCCTCGGCGCGATGGGCGCCGCGCT
GGCCCGCGCCTGGCTCGCCGCCCGCCACCCGCTCACCGTCTGGAACCGCACCCCGACCCGCGCCGCGG
CGATATCCGCCGAGGGAGCGACGGTCGCCGACAGCGCCGCCGAGGCCGTCGCCGCGAACACCCTTGTC
GTCGTCTGCCTGCTGGACGACGCCTCGGTCGAGGAGGTGCTGGCCGGCGCCGACCTGGCCGGCAGGGA
TCTGGTCAACCTGACCACGGGTACGCCCGCCCAGGCCCGCGCCCGGGCCGACTGGGCCCGCGAGCGCG
GCGCTCGCTACCTGGACGGCGGAATCATGGCCGTCCCTCCGATGATCGGCGTCCCGGACGCCGGCGGC
TACGTCTTCTACAGCGGCTCCCGCGAGCTGTTCGAACGGCACCGGGAGACGCTCGCCGTCCCCGCCGG
CACCACCTACGTCGGCCGGGACGCGGGCTTCGCCGCCCTGCACGACGTGGCCCTGCTCAGCGCCATGT
ACGGCATGTTCGCCGGGGCAGCGCACGCTTTCGCCCTGATCCGCCGGGAGGACATCGACCCCGCGTCG
CTGGCCCCGCTGCTCGCCGACTGGCTCGTCGCGATGGCCCCGACCGTTCACCAGACCGCCGACCAGCT
GCGCAGCGGCGACTACACGAAGGGCGTCGTCTCCAATCTCGCCATGCAGGTGGCCGGCACACCGACGT
TCCTGCGCACCGCGGCAGAGCAGGGCGTCAGCCCGGAACTGCTCAGCCCCTACTTCGAGCTGATGCGC
CGCCGCCTGGCCGAGGGCAGCGGCGAGGAGGACCTGACGGGCGTCATCGACCTGCTGGTGCGCtga
[882 bp]

Optimised Gene Sequence incl. 'Stuffer DNA' (*GeneArt*):

GTGCCGCGCGGCAGCCATATGGCACCGGATACCGTTGAAAAAACACCGGTTACCCTGCTGGGTCTGGG
TGCAATGGGTGCAGCACTGGCACGTGCATGGCTGGCAGCACGTCATCCGCTGACCGTTTGGAATCGTA
CCCCGACCCGTGCAGCAGCAATTAGCGCAGAAGGTGCAACCGTTGCAGATAGCGCAGCAGAAGCAGTT
GCAGCAAATACCCTGGTTGTTGTTTGTCTGCTGGATGATGCAAGCGTTGAAGAAGTTCTGGCAGGCGC
AGATCTGGCAGGTCGTGATCTGGTTAATCTGACCACCGGTACACCGGCACAGGCACGCGCACGTGCAG
ATTGGGCACGTGAACGTGGTGCACGTTATCTGGATGGTGGTATTATGGCAGTTCCGCCTATGATTGGT
GTTCCGGATGCCGGTGGTTATGTGTTTTATAGCGGTAGCCGTGAACTGTTTGAACGTCATCGTGAAAC
CCTGGCCGTTCCGGCAGGCACCACCTATGTTGGTCGTGATGCAGGTTTTGCAGCCCTGCATGATGTTG
CACTGCTGAGCGCAATGTATGGTATGTTTGCCGGTGCCGCACATGCATTTGCACTGATTCGTCGTGAA
GATATTGATCCGGCAAGCCTGGCACCGCTGCTGGCAGATTGGCTGGTTGCAATGGCACCGACCGTTCA
TCAGACCGCAGATCAGCTGCGTAGCGGTGATTATACCAAAGGTGTTGTTAGCAATCTGGCAATGCAGG
TTGCAGGCACCCCGACCTTTCTGCGTACCGCAGCCGAACAGGGTGTTAGTCCGGAACTGCTGAGTCCG
TATTTTGAACTGATGCGTCGCCGTCTGGCCGAAGGTAGTGGTGAAGAGGATCTGACCGGTGTTATTGA
TCTGCTGGTTCGTtaaCTCGAGCCACTGAGATCCGGC [921 bp]

Protein Sequence incl. N-terminal His-tag:

MGSSHHHHHHSSGLVPRGSHMAPDTVEKTPVTLLGLGAMGAALARAWLAARHPLTVWNRTPTRAAAIS
AEGATVADSAAEAVAANTLVVVCLLDDASVEEVLAGADLAGRDLVNLTTGTPAQARARADWARERGAR
YLDGGIMAVPPMIGVPDAGGYVFYSGSRELFERHRETLAVPAGTTYVGRDAGFAALHDVALLSAMYGM
FAGAAHAFALIRREDIDPASLAPLLADWLVAMAPTVHQTADQLRSGDYTKGVVSNLAMQVAGTPTFLR
TAAEQGVSPELLSPYFELMRRRLAEGSGEEDLTGVIDLLVR [313 aa]

Predicted Properties (*ExPASy* ProtParam):

Isoelectric point:	5.71
Molecular weight:	32,946 Da
Absorbance at 280 nm:	32,430 M^{-1} cm^{-1}

IRED-D (V7GV82)

Native Gene Sequence (Genbank ID AYWV01000016.1):

ATGAGCGACATAACAGTGATCGGTCTCGGCGCCATGGGCACGGCGCTTGCCGAAGCCTTTCTGAACCA
GGGACATGCGGTGACGGTGTGGAACCGTTCTCCGGCAAAGGCTGAGGCTTTGGCAGCCAAGGGCGCCA
CTGTGGCCAAGAGTGTCGAGGAAGCCGTCAGATCGAGTCCACTGATCGTGGCGTGCCTGCTTGTCTAT
GACACCGTTCGTGAGGTGCTCGGCCCAAGCCGAGACGCGCTGTCGGGCCGCACATTGGTCAATCTCAC
CAACGGCACGCCCGAGCAGGCGCGGGCGATGTCGGGCTGGGCGGTGAGCCAGGGCGCGAGCTATATCG
ACGGCGGCATCATGGCGGTGCCGCCGATGATCGGCGGGCCGCACGCCTTGATTCTCTACAGCGGTTCG
CGCCAGGCGTTCGATGCCTGTTCGGGGCAGCTCGGAGCGCTCGGCACCAGCAAATTCCTCGGTGAGGA
TGCTGGCCTCGCGCCGCTCTATGATATCTCGCTTCTGACCGGCATGTACGGCATGTTCGCGGGCGTTC
TGCAGGCGTTGGCATTGACGGGCGCAGCAGGAATCCCGGCCGGCGAGTTCATGCCGCTGCTGGCCTCC
TGGCTTCAGTCGATGCAAGGCCTGCTGCCGAAGTGGGCGGAACAGATCGACAGTGGCGATCACACCAG
CAATGTCGTTTCCAACCTGGGCATGCAGGTTGATGCCTACGTCAATCTCATCGATGCCAGCAGGTCGG
CTGACGTCAGCACCGAACTCGTCTTGCCGATGCAAAGCCTGATGAAGCGCGGGGTGGCTGCCGGCCAG
GCCAATGCCGATCTTACAAGCCTGGTCGCACTGCTCCAGCTCTCGAAACAAGGCGCTtga [876 bp]

Optimised Gene Sequence incl. 'Stuffer DNA' (*GeneArt*):

GTGCCGCGCGGCAGCCATATGAGCGATATTACCGTTATTGGTCTGGGTGCAATGGGCACCGCACTGGC
AGAAGCATTTCTGAATCAGGGTCATGCAGTTACCGTTTGGAATCGTAGTCCGGCAAAAGCAGAAGCCC
TGGCAGCAAAAGGTGCAACCGTTGCAAAAAGCGTTGAAGAAGCAGTTCGTAGCAGTCCGCTGATTGTT
GCATGTCTGCTGGTTTATGATACCGTTCGTGAAGTTCTGGGTCCGAGCCGTGATGCACTGAGCGGTCG
TACCCTGGTTAATCTGACCAATGGTACACCGGAACAGGCACGTGCAATGAGCGGTTGGGCAGTTAGCC
AGGGTGCAAGCTATATTGATGGTGGTATTATGGCAGTTCCGCCTATGATTGGTGGTCCGCATGCACTG
ATTCTGTATAGCGGTAGCCGTCAGGCATTTGATGCGTGTAGCGGTCAGCTGGGTGCCCTGGGTACAAG
CAAATTTCTGGGTGAAGATGCAGGTCTGGCACCGCTGTATGATATTAGCCTGCTGACCGGTATGTATG
GTATGTTTGCCGGTGTTCTGCAGGCACTGGCACTGACCGGTGCAGCCGGTATTCCGGCAGGCGAATTT
ATGCCGCTGCTGGCAAGCTGGCTGCAGAGCATGCAGGGTCTGCTGCCGAAATGGGCAGAGCAGATTGA
TAGCGGTGATCATACCAGCAATGTTGTTAGCAATCTGGGTATGCAGGTTGATGCCTATGTGAATCTGA
TTGATGCAAGCCGTAGCGCAGATGTTAGCACCGAACTGGTTCTGCCGATGCAGAGCCTGATGAAACGT
GGTGTTGCAGCAGGTCAGGCAAATGCAGATCTGACCAGCCTGGTTGCACTGCTGCAGCTGAGCAAACA
GGGTGCAtaaCTCGAGCCACTGAGATCCGGC [915 bp]

Protein Sequence incl. N-terminal His-tag:

MGSSHHHHHHSSGLVPRGSHMSDITVIGLGAMGTALAEAFLNQGHAVTVWNRSPAKAEALAAKGATVA
KSVEEAVRSSPLIVACLLVYDTVREVLGPSRDALSGRTLVNLTNGTPEQARAMSGWAVSQGASYIDGG
IMAVPPMIGGPHALILYSGSRQAFDACSGQLGALGTSKFLGEDAGLAPLYDISLLTGMYGMFAGVLQA
LALTGAAGIPAGEFMPLLASWLQSMQGLLPKWAEQIDSGDHTSNVVSNLGMQVDAYVNLIDASRSADV
STELVLPMQSLMKRGVAAGQANADLTSLVALLQLSKQGA [311 aa]

Predicted Properties (*ExPASy* ProtParam):

Isoelectric point:	5.89
Molecular weight:	32,083 Da
Absorbance at 280 nm:	31,065 M^{-1} cm^{-1}

IRED-E (J7LAY5)

Native Gene Sequence (Genbank ID CP003788.1):

ATGAAGAACGACGGGGTGACGAAGGGATCGGTCGCCCTTCTGGGGCTCGGCGAGATGGGGCGGGTGCT
CGCGGAACGGCTCCTCGACGCCGGATATCCGGTGACGGTCTGGAACCGGACCCCGGGCCGGGACACGG
CGCTCGTGGAACGCGGGGCACGGCGAGCGGAGACGGTACGGGAGGCGGTCACCGCCGCGACGACCGTC
GTCACCTGCCTCTTCGACCACGCGTCGGTGCGGGAGACACTCGAACCGGTGGGCGCCGACCTGGCCGG
CAGGACCCTGGTCGACCTCACCACGACGACCCCGAACGAGGCCCGATGGCTGGGCGGATGGGCCGAGG
AACGGGGCATCGAGCACCTCGACGGCGCCATCATGGCGACCCCGTCGATGATCGGCGCTCCCGAGGCG
TCGCTCCTCTACAGCGGGTCCGCCGAGGCCTTCGGACGCCACAGGACCCTCTTCGAGGTCTGGGGGAG
CGCCACCTACGACGGAGCGGACCACGGGGCGGCGTCGCTGTTCGATCTGGCCCTCCTGTCGGGGATGT
ACACGATGTTCACCGGTTTCGCGCACGGGGCCGCCATGGTCACGTCGGCGGGGGTCACCGCGGAGGAG
TTCGCCCATCGATCCGCACGGCTCTTGTCCGCCATGACCGGGGTGTTCCCGATGACGGCGAAGGTGAT
CGACGAGGGCGACTACACCGGGCCCGGGCAGAGCCTCGAATGGACCGCCACGGCCTTGGACACCATCG
CGCGCGCCTCGGCCGAGCAGGGGGTCTCTCCCGGGCCGATCGAGATGACGCGGGCGCTCGTCCTCGCC
CAGATCGAGGCCGGATACGGGAACGAGAACTCCGATCGGATCTACGAGGAGCTGCGGGCCGGGtga
[882 bp]

Optimised Gene Sequence incl. 'Stuffer DNA' (*GeneArt*):

GTGCCGCGCGGCAGCCATATGAAAAATGATGGTGTTACCAAAGGTAGCGTTGCACTGCTGGGTCTGGG
TGAAATGGGTCGTGTTCTGGCAGAACGTCTGCTGGATGCAGGTTATCCGGTTACCGTTTGGAATCGTA
CACCGGGTCGTGATACCGCACTGGTTGAACGTGGTGCACGTCGTGCAGAAACCGTTCGTGAAGCAGTT
ACCGCAGCAACCACCGTTGTTACCTGTCTGTTTGATCATGCAAGCGTGCGTGAAACCCTGGAACCGGT
TGGTGCAGATCTGGCAGGTCGTACCCTGGTTGATCTGACCACCACCACCCCGAATGAAGCACGTTGGC
TGGGTGGTTGGGCAGAAGAACGCGGTATTGAACATCTGGATGGTGCAATTATGGCAACCCCGAGCATG
ATTGGTGCACCGGAAGCAAGCCTGCTGTATAGCGGTAGCGCAGAAGCATTTGGTCGTCATCGTACACT
GTTTGAAGTTTGGGGTAGCGCAACCTATGATGGTGCCGATCATGGTGCAGCCAGCCTGTTCGATCTGG
CACTGCTGAGCGGTATGTATACCATGTTTACCGGTTTTGCACATGGTGCCGCAATGGTTACCAGTGCC
GGTGTGACCGCTGAAGAATTTGCACATCGTAGCGCACGCCTGCTGAGTGCAATGACCGGTGTTTTTCC
GATGACCGCAAAAGTTATTGATGAAGGTGATTATACCGGTCCGGGTCAGAGCCTGGAATGGACCGCAA
CAGCACTGGATACAATTGCACGTGCCAGCGCAGAACAGGGTGTTAGTCCGGGTCCGATTGAAATGACC
CGTGCACTGGTTCTGGCACAGATTGAAGCAGGTTATGGTAATGAAAATAGCGATCGCATCTATGAAGA
ACTGCGTGCAGGTtaaCTCGAGCCACTGAGATCCGGC [921 bp]

Protein Sequence incl. N-terminal His-tag:

MGSSHHHHHHSSGLVPRGSHMKNDGVTKGSVALLGLGEMGRVLAERLLDAGYPVTVWNRTPGRDTALV
ERGARRAETVREAVTAATTVVTCLFDHASVRETLEPVGADLAGRTLVDLTTTTPNEARWLGGWAEERG
IEHLDGAIMATPSMIGAPEASLLYSGSAEAFGRHRTLFEVWGSATYDGADHGAASLFDLALLSGMYTM
FTGFAHGAAMVTSAGVTAEEFAHRSARLLSAMTGVFPMTAKVIDEGDYTGPGQSLEWTATALDTIARA
SAEQGVSPGPIEMTRALVLAQIEAGYGNENSDRIYEELRAG [313 aa]

Predicted Properties (*ExPASy* ProtParam):

Isoelectric point:	5.34
Molecular weight:	33,135 Da
Absorbance at 280 nm:	37,930 M^{-1} cm^{-1}

IRED-F (V6KA13)

Native Gene Sequence (Genbank ID AWQW01000146.1):

ATGACCACCGAGCCCTCGCTCCCCACCGTCTCCATCGTCGGCCTCGGCAACCTGGGCCGCGCCCTGGC
CGGCGCCTTCCTCGACCAGGGCTACCGCACCACGGTCTGGAACCGTTCCCCGGCCAAGGCCGACGACC
TCGTCGCCAGAGGCGCGCACCGGGCGACGACCGCCGCCGAAGCCCTCGCGGCGGGCGAACTCGTCATC
GTCTGCGTGCTGGACTACGACACCGTGAACCGGCTCCTGACACCGGCCGCCGACGCGCTCCGGGGACG
CGTCCTGCTCAACCTCACCTCCGGAACCCCGGAGCCCGCCCGCGAGTTGGCGGCCTGGGTGACCGGCC
AGGGCGCCGACTACCTGGACGGCGCGGTCTACGCCGTACCGCAGACGATCGGCACGGCGGACGCGTTC
GTCCTCTACAGCGGCTCGTCCGCCGTCTTCGAGACGTACCGCGAGCAGCTGGACCTCCTGGGCGCCCC
CACCTTCGTCGGCACCGACCCCGGTCTGGCCTCGCTGTACGACGTCGCGCTGCTCAGCGGCATGTACG
GGATGTTCGCGGGCTTCTTCCAGTCGGTCGCGGTCGCCGACTCGGCCCAGATCAAGGCCACGGACATC
ACCGCCCTCCTGGTCCCCTGGCTGAACGGGGCAGCCGCCGCGCTGCCCGGCTTCGCGGCCGAGATCGA
CTCCGGCGACTACACGACGGAGACGTCCAACCTCGACATCAACACGGTGGGCCTGGCGAACATACTGA
CGGCCACGAAGGCCCAGGGCGTCGGGGTGGACCTGCTGACCCCGCTCCAGACTCTCTTCGAGCGCCAG
ATCGCCCAGGGCCACGGGGCGTCGAGCCTCTCGCGGGCGATCGAATCACTCCGACCGCCGCGCtga
[882 bp]

Optimised Gene Sequence incl. 'Stuffer DNA' (*GeneArt*):

GTGCCGCGCGGCAGCCATATGACCACCGAACCGAGCCTGCCGACCGTTAGCATTGTTGGTCTGGGTAA
TCTGGGTCGTGCACTGGCAGGCGCATTTCTGGATCAGGGTTATCGTACCACCGTTTGGAATCGTAGTC
CGGCAAAAGCAGATGATCTGGTTGCACGTGGTGCACATCGTGCCACCACCGCAGCCGAAGCTCTGGCA
GCCGGTGAACTGGTTATTGTTTGTGTTCTGGATTATGATACCGTGAATCGTCTGCTGACACCGGCAGC
AGATGCACTGCGTGGTCGTGTTCTGCTGAATCTGACCAGCGGTACACCGGAACCGGCACGTGAACTGG
CAGCATGGGTTACCGGTCAGGGTGCAGATTATCTGGATGGTGCAGTTTATGCAGTTCCGCAGACCATT
GGCACCGCAGATGCATTTGTTCTGTATAGCGGTAGCAGCGCAGTTTTTGAAACCTATCGTGAACAGCT
GGATCTGCTGGGTGCACCGACCTTTGTTGGCACCGATCCGGGTCTGGCAAGCCTGTATGATGTTGCAC
TGCTGAGCGGTATGTATGGTATGTTTGCAGGTTTTTTCAGAGCGTTGCAGTTGCAGATAGCGCACAG
ATTAAAGCAACCGATATTACCGCACTGCTGGTTCCGTGGCTGAATGGTGCAGCAGCAGCCCTGCCTGG
TTTTGCAGCAGAAATTGATAGCGGTGATTATACCACCGAAACCAGCAATCTGGATATTAACACCGTGG
GTCTGGCCAATATTCTGACCGCAACCAAAGCACAGGGTGTTGGTGTTGATCTGCTGACTCCGCTGCAG
ACCCTGTTTGAACGTCAGATTGCACAGGGTCATGGTGCAAGCAGTCTGAGCCGTGCAATTGAAAGCCT
GCGTCCGCCTCGTtaaCTCGAGCCACTGAGATCCGGC [921 bp]

Protein Sequence incl. N-terminal His-tag:

MGSSHHHHHHSSGLVPRGSHMTTEPSLPTVSIVGLGNLGRALAGAFLDQGYRTTVWNRSPAKADDLVA
RGAHRATTAAEALAAGELVIVCVLDYDTVNRLLTPAADALRGRVLLNLTSGTPEPARELAAWVTGQGA
DYLDGAVYAVPQTIGTADAFVLYSGSSAVFETYREQLDLLGAPTFVGTDPGLASLYDVALLSGMYGMF
AGFFQSVAVADSAQIKATDITALLVPWLNGAAAALPGFAAEIDSGDYTTETSNLDINTVGLANILTAT
KAQGVGVDLLTPLQTLFERQIAQGHGASSLSRAIESLRPPR [313 aa]

Predicted Properties (*ExPASy* ProtParam):

Isoelectric point:	5.15
Molecular weight:	32,611 Da
Absorbance at 280 nm:	29,910 M^{-1} cm^{-1}

IRED-G (L8EIW6)

Native Gene Sequence (Genbank ID ANSJ01000148.1):

ATGGCAGCCACCCCCACCAACCCGTCCACCGACTCCGGCAAGACCCCCGTGACCGTGCTCGGCCTCGG
CGCGATGGGCCGGGCGCTGGCCGGCGCCTTCCTCAAGGCCGGACACCCCACCACCGTATGGAACCGCT
CCGAGCACAAGGCCGACGAGCTGGTCGCCCGCGGCGCCGTACGGGCCGGGAGCGTCGCCGAGGCGGTG
GCCGCCAGTCCGCTGATCGTCGTCTGCGTCGTGGACTACGAGGTCAGCCACCGCATCCTGGAGCCGGT
CGGCGCCGACCTGGCCGGGCGGGTGCTGGTCAACCTCACCTCCGACACCCCGGTACGCTCCCGGCGCG
CCGCCGAGTGGGCCGCCGGGCACGGCGTCGAGTACCTGGACGGGGCGATCATGGTGCCGACGCCGGTC
ATCGGCACACCGGAGGCGACGGTCCTCTACAGCGGCTCGCGGCGGGCCTTCGACACGTACGAGGAGAC
GCTGAAGGCGCTGGGCGGCAAGGCGCCCTTCCTCGGCACGGACCACGGCGTCGCGGCGGTCTACGACC
TGGCGATGCTCAGCTTCTTCTACTCCGGCATGGCGGGCCTCGCGCACGCCTTCACGCTGGCCGGGGAG
GAAGGCGTCCCGGCCACGGACCTGGCCCCGTTCCTGGACGTGATCACGGGCATCTTCCCGCCGATCGC
GAAGGGCATGGCCGACGACCTCGTCGGCGGGCGGCTCGACGGCGCGGGGGAGGGCAACATCGTCATGG
AGGCGGCGGGCATCGCGCACATCGTCGAGGCGTCCCGGGACCGCGGCGTCAACACCGATGTGCTCGAC
GCGCTCAAGGCGCTGATGGACCGCACGATCGCCGCCGGACACGGGGAGTCGGAGTTCGTACGGGTGAC
GGAGGCGATGCGGGGGGCGTACGCCtga [912 bp]

Optimised Gene Sequence incl. 'Stuffer DNA' (*GeneArt*):

GTGCCGCGCGGCAGCCATATGGCAGCAACCCCGACCAATCCGAGCACCGATAGCGGTAAAACACCGGT
TACCGTTCTGGGTCTGGGTGCAATGGGTCGTGCACTGGCAGGCGCATTTCTGAAAGCAGGTCATCCGA
CCACCGTTTGGAATCGTAGCGAACATAAAGCAGATGAACTGGTTGCACGTGGTGCAGTTCGTGCAGGT
AGCGTTGCAGAAGCAGTTGCAGCAAGTCCGCTGATTGTTGTTTGTGTTGTTGATTATGAAGTGAGCCA
TCGTATTCTGGAACCGGTTGGTGCAGATCTGGCAGGTCGTGTTCTGGTTAATCTGACCAGCGATACAC
CGGTTCGTAGCCGTCGTGCAGCAGAATGGGCAGCCGGTCATGGTGTTGAATATCTGGATGGTGCAATT
ATGGTTCCGACACCGGTGATTGGTACACCGGAAGCAACCGTTCTGTATAGCGGTTCACGTCGTGCATT
TGATACCTATGAAGAAACCCTGAAAGCACTGGGTGGTAAAGCACCGTTTCTGGGTACAGATCATGGTG
TGGCAGCAGTTTATGATCTGGCAATGCTGAGCTTTTTCTATAGCGGTATGGCAGGTCTGGCACATGCA
TTTACCCTGGCTGGTGAAGAAGGTGTTCCGGCAACCGATCTGGCACCTTTTCTGGATGTTATTACCGG
TATTTTTCCGCCTATTGCAAAAGGTATGGCCGATGATCTGGTTGGTGGTCGTCTGGATGGCGCAGGCG
AAGGTAATATTGTTATGGAAGCAGCAGGTATTGCCCATATTGTTGAAGCAAGCCGTGATCGTGGTGTT
AATACCGATGTTCTGGATGCACTGAAAGCCCTGATGGATCGTACCATTGCCGCAGGTCATGGCGAAAG
CGAATTTGTTCGTGTTACCGAAGCAATGCGTGGTGCCTATGCAtaaCTCGAGCCACTGAGATCCGGC
[951 bp]

Protein Sequence incl. N-terminal His-tag:

MGSSHHHHHHSSGLVPRGSHMAATPTNPSTDSGKTPVTVLGLGAMGRALAGAFLKAGHPTTVWNRSEH
KADELVARGAVRAGSVAEAVAASPLIVVCVVDYEVSHRILEPVGADLAGRVLVNLTSDTPVRSRRAAE
WAAGHGVEYLDGAIMVPTPVIGTPEATVLYSGSRRAFDTYEETLKALGGKAPFLGTDHGVAAVYDLAM
LSFFYSGMAGLAHAFTLAGEEGVPATDLAPFLDVITGIFPPIAKGMADDLVGGRLDGAGEGNIVMEAA
GIAHIVEASRDRGVNTDVLDALKALMDRTIAAGHGESEFVRVTEAMRGAYA [323 aa]

Predicted Properties (*ExPASy* ProtParam):

Isoelectric point:	5.71
Molecular weight:	33,459 Da
Absorbance at 280 nm:	21,430 M^{-1} cm^{-1}

IRED-H (I8QLV7)

Native Gene Sequence (Genbank ID CM001489.1):

ATGAATTCCCACCCACCTGCCGTCACCGTCATCGGACTGGGCCTGATGGGCTCGGCGCTCGCCGCCGT
GCTGCTGGATGCCGGCTGCCCGACGACCGTGTGGAACCGCTCGGCGCACAAGGCCCAGTCGCTGGTCG
ACCGGGGTGCACGCCTGACCGGGACGCCGCGGGAGGCGGTCGAGGCGAGTCCATTCGTCATCGTCTGT
GTGCTCGACTACGACGTGCTGTACTCCGTTCTCGCTCCCTCCGTAGATGCCCTCGCCGGCAAGGTTCT
GGTCAACCTCACCTCCGGCTCGCCGGAACAGGCCCGCGAGGCGATGGCATGGGCCCGGTCCCACGGAG
CCGACTACCTGGACGGCGCGATCATGACCACGCCGCCGGGTGTCGGCAGCCCCGAGATGATGTTCCTC
TACGGCGGTCCGGACGACGTCTTCGACGCCCATCGTCAGACCCTGGCGTTCCTGGGTGACCCCCTGCA
CCTGGGAGACGACCCCGGCCTGGCTTCCCTCTACGACGTCGCGCTGCTCGGCCTGATGTGGTCCACGC
TGACCGGCTGGCTGCACGGCACCGCCCTGGTCGGGGCGGAGAAGACGTCCGCCACGACTTTCACCCCC
TTCGCGGTCCGCTGGCTGACCGCCGTGGCCGGTTTCCTCACCACGTACGCACCCCAGGTGGACGCCGG
CCGCTACCCGGGTGACGACGCCACGGTCGACGTCCAGATCGCGTCCATAGACCATCTCCTGCACGCCG
CGGCCTCCCGGGGCGTCGACAACGCCCTGCCCGAACTCCTGAAATCCGTCATGGAGCAGGCCAGGGCC
GCGGGCCACGGCTCCGACAGCTACGCGAGCGTCATCGAGGTGCTGAGGAGCCCCGCCCCCGACCCGGA
CGAGCACAACCGCtga [900 bp]

Optimised Gene Sequence incl. 'Stuffer DNA' (*GeneArt*):

GTGCCGCGCGGCAGCCATATGAATAGCCATCCGCCTGCAGTTACCGTTATTGGTCTGGGTCTGATGGG
TAGCGCACTGGCAGCAGTTCTGCTGGATGCAGGTTGTCCGACCACCGTTTGGAATCGTAGCGCACATA
AAGCACAGAGCCTGGTTGATCGTGGTGCACGTCTGACCGGTACACCGCGTGAAGCAGTTGAAGCAAGC
CCGTTTGTTATTGTTTGTGTGCTGGATTATGATGTCCTGTATAGCGTTCTGGCACCGAGCGTTGATGC
CCTGGCAGGTAAAGTTCTGGTTAATCTGACCAGCGGTAGTCCGGAACAGGCACGTGAAGCAATGGCAT
GGGCACGTAGCCATGGTGCAGATTATCTGGATGGTGCCATTATGACCACCCCTCCGGGTGTTGGTTCA
CCGGAAATGATGTTTCTGTATGGTGGTCCGGATGATGTTTTTGATGCACATCGTCAGACCCTGGCATT
TCTGGGTGATCCGCTGCACCTGGGTGATGATCCGGGTCTGGCAAGCCTGTATGATGTTGCACTGCTGG
GCCTGATGTGGTCAACCCTGACCGGTTGGCTGCATGGCACCGCACTGGTTGGTGCAGAAAAAACCAGC
GCAACCACCTTTACCCCGTTTGCAGTTCGTTGGCTGACCGCAGTTGCAGGCTTTCTGACCACCTATGC
ACCTCAGGTTGATGCAGGTCGTTATCCTGGTGATGATGCAACCGTTGATGTTCAGATTGCAAGCATTG
ATCATCTGCTGCATGCCGCAGCAAGCCGTGGTGTTGATAATGCACTGCCGGAACTGCTGAAAAGCGTT
ATGGAACAAGCCCGTGCAGCAGGTCATGGTAGCGATAGCTATGCAAGCGTTATTGAAGTTCTGCGTAG
TCCGGCACCGGATCCTGATGAACATAATCGTtaaCTCGAGCCACTGAGATCCGGC [939 bp]

Protein Sequence incl. N-terminal His-tag:

MGSSHHHHHHSSGLVPRGSHMNSHPPAVTVIGLGLMGSALAAVLLDAGCPTTVWNRSAHKAQSLVDRG
ARLTGTPREAVEASPFVIVCVLDYDVLYSVLAPSVDALAGKVLVNLTSGSPEQAREAMAWARSHGADY
LDGAIMTTPPGVGSPEMMFLYGGPDDVFDAHRQTLAFLGDPLHLGDDPGLASLYDVALLGLMWSTLTG
WLHGTALVGAEKTSATTFTPFAVRWLTAVAGFLTTYAPQVDAGRYPGDDATVDVQIASIDHLLHAAAS
RGVDNALPELLKSVMEQARAAGHGSDSYASVIEVLRSPAPDPDEHNR [319 aa]

Predicted Properties (*ExPASy* ProtParam):

Isoelectric point:	5.44
Molecular weight:	33,531 Da
Absorbance at 280 nm:	39,545 M^{-1} cm^{-1}

IRED-I (M4ZS15)

Native Gene Sequence (Genbank ID AB747175.1):

ATGAGCAAGCAGTCGGTAACGGTCATCGGTCTGGGCCCGATGGGCCAGGCGATGGTGAACACCTTCCT
GGACAACGGCCACGAGGTGACCGTGTGGAACCGGACCGCCAGCAAGGCCGAGGCGCTCGTGGCCCGGG
GTGCCGTACTGGCTCCCACCGTCGAGGACGCGCTCAGCGCCAATGAGCTGATCGTGCTCAGCCTGACG
GACTACGACGCGGTGTACGCCATTCTTGAGCCGGTGACGGGTTCCCTGTCCGGCAAGGTCATCGCCAA
CCTCAGCTCCGACACCCCGGACAAGGCCCGCGAGGCGGCCAAGTGGGCGGCCAAGCACGGTGCGAAGC
ACCTCACCGGTGGTGTGCAGGTGCCGCCGCCGCTGATCGGCAAGCCCGAGTCCTCCACCTACTACAGC
GGTCCCAAGGATGTCTTCGACGCCCATGAGGACACCCTGAAGGTCCTCACCAACGCGGACTACCGCGG
CGAGGACGCCGGCCTCGCGGCCATGTACTACCAGGCCCAGATGACCATCTTCTGGACCACGATGCTGA
GCTACTACCAGACCCTCGCGCTGGGCCAGGCCAACGGTGTCTCGGCGAAGGAACTGCTGCCCTACGCC
ACGATGATGACGTCGATGATGCCGCACTTCCTGGAGCTGTACGCCCAGCACGTGGACTCCGCGGACTA
CCCGGGCGACGTGGACCGGCTCGCGATGGGGGCGGCCAGTGTCGACCACGTCCTGCACACGCACCAGG
ACGCGGGCGTCAGCACCGTGCTGCCGGCCGCCGTCGCCGAGATCTTCAAGGCGGGCATGGAGAAGGGC
TTCGCCGAGAACAGCTTCTCCAGCCTCATCGAGGTGCTCAAGAAGCCGGCGGTCtga [873 bp]

Optimised Gene Sequence incl. 'Stuffer DNA' (*GeneArt*):

GTGCCGCGCGGCAGCCATATGAGCAAACAGAGCGTTACCGTTATTGGTCTGGGTCCGATGGGTCAGGC
AATGGTTAATACCTTTCTGGATAATGGTCATGAAGTGACCGTTTGGAATCGTACCGCAAGCAAAGCCG
AAGCACTGGTTGCACGTGGTGCAGTTCTGGCACCGACCGTTGAAGATGCACTGAGCGCAAATGAACTG
ATTGTTCTGAGCCTGACCGATTATGATGCAGTTTATGCAATTCTGGAACCGGTTACCGGTAGCCTGAG
CGGTAAAGTTATTGCAAATCTGAGCAGCGATACACCGGATAAAGCACGTGAAGCAGCAAAATGGGCAG
CAAAACATGGTGCAAAACATCTGACCGGTGGTGTTCAGGTTCCGCCTCCGCTGATTGGTAAACCGGAA
AGCAGCACCTATTATAGCGGTCCGAAAGATGTTTTTGATGCCCATGAAGATACCCTGAAAGTTCTGAC
CAATGCAGATTATCGTGGTGAAGATGCCGGTCTGGCAGCAATGTATTATCAGGCACAGATGACCATTT
TTTGGACCACCATGCTGAGCTATTATCAGACCCTGGCACTGGGCCAGGCAAATGGTGTTAGCGCCAAA
GAACTGCTGCCGTATGCAACCATGATGACCAGCATGATGCCGCATTTTCTGGAACTGTATGCACAGCA
TGTTGATAGTGCCGATTATCCGGGTGATGTTGATCGTCTGGCAATGGGTGCAGCAAGCGTTGATCATG
TTCTGCATACCCATCAGGATGCCGGTGTTAGCACCGTTCTGCCTGCAGCAGTTGCAGAAATCTTTAAA
GCAGGTATGGAAAAAGGCTTTGCCGAAAATAGCTTTAGCAGCCTGATTGAGGTTCTGAAAAAACCGGC
AGTTTtaaCTCGAGCCACTGAGATCCGGC [912 bp]

Protein Sequence incl. N-terminal His-tag:

MGSSHHHHHHSSGLVPRGSHMSKQSVTVIGLGPMGQAMVNTFLDNGHEVTVWNRTASKAEALVARGAV
LAPTVEDALSANELIVLSLTDYDAVYAILEPVTGSLSGKVIANLSSDTPDKAREAAKWAAKHGAKHLT
GGVQVPPPLIGKPESSTYYSGPKDVFDAHEDTLKVLTNADYRGEDAGLAAMYYQAQMTIFWTTMLSYY
QTLALGQANGVSAKELLPYATMMTSMMPHFLELYAQHVDSADYPGDVDRLAMGAASVDHVLHTHQDAG
VSTVLPAAVAEIFKAGMEKGFAENSFSSLIEVLKKPAV [310 aa]

Predicted Properties (*ExPASy* ProtParam):

Isoelectric point:	5.76
Molecular weight:	33,103 Da
Absorbance at 280 nm:	34,380 M^{-1} cm^{-1}

IRED-J (D2PR38)

Native Gene Sequence (Genbank ID CP001736.1):

ATGCCCCCCACCGACCGCACCCCCGTCACGCTGATCGGCCTCGGCCCGATGGGACAGGCGATGACCCG
CGCGCTGCTCGCGGCCGGACACCCGGTGACCGTCTGGAACCGGACCCCCGCCCGGGCCGCCGGCGTCG
TGGCCGACGGCGCCGTTCTCGCCGCGAGTCCGGTCGAGGCCGTCGAAGCCGGCGACCTGGTCATCCTC
AGCCTGACCGACTACCAGGCGATGTACGACGTACTGGAGCCGGCCACCGGCTCGCTCGCGGGCCGCAC
CGTCGTCAACCTCAGCTCCGACACCCCGGACCGCACCCGCGCGGCCGCGGACTGGGCCACCGAGCACG
GCGCGACGTTCCTGACCGGCGGGGTGATGATCCCCGCGCCGATGGTTGGCACCGAGGAGGCCTACGTC
TACTACAGCGGCCCCGCCGAGGTCTTCGAGAAGCACCGGACCACGCTCACCGTGATCGGGGCTCCCCG
CTACCTCGGCGAAGACACGGGCCTGGCACAGCTGATGTATCAGGCGCAGCTCGACGTCTTCCTGACCA
CGCTGTCGTCGCTGATGCACGCAACAGCCCTGCTCGGTACGGCGGGCGTCAGCGCGGCGGAGTCGATG
CCCGAGCTGATCGGGATGCTGCGCACCGTGCCGGCGATGCTGGAGGCGGGCGGGGAGAATCCCGGCGC
GGACATCGACGCCGACAAGCACCCGGGCGACCTGAGCACGATCACGATGATGGGCGCGACCGCCGACC
ACATCGTCGGGGCGAGCGAGACGGCCGGCATCGACCTCGCGCTGCCGCGAGCGGTGCAGGCGCACTAC
CGCCGGGCGATCGAGAACGGCCACGGCGGAGACAACTGGACGCGGATCATCGACGGCATCCGCAGCCC
GCGCtga [891 bp]

Optimised Gene Sequence incl. 'Stuffer DNA' (*GeneArt*):

GTGCCGCGCGGCAGCCATATGCCTCCGACCGATCGTACACCGGTTACCCTGATTGGTCTGGGTCCGAT
GGGTCAGGCAATGACCCGTGCACTGCTGGCAGCAGGTCATCCGGTTACCGTTTGGAATCGCACACCGG
CACGTGCAGCCGGTGTTGTTGCAGATGGTGCAGTTCTGGCAGCAAGTCCGGTTGAAGCAGTTGAAGCC
GGTGATCTGGTTATTCTGAGCCTGACCGATTATCAGGCCATGTATGACGTTCTGGAACCGGCAACCGG
TAGCCTGGCAGGTCGTACCGTTGTTAATCTGAGCAGCGATACACCGGATCGTACCCGTGCAGCAGCAG
ATTGGGCAACCGAACATGGTGCAACCTTTCTGACCGGTGGTGTTATGATTCCGGCACCGATGGTTGGC
ACCGAAGAGGCCTATGTGTATTATAGCGGTCCGGCAGAAGTTTTTGAAAAACATCGTACCACCCTGAC
CGTTATTGGTGCACCGCGTTATCTGGGTGAAGATACCGGTCTGGCACAGCTGATGTATCAGGCCCAGC
TGGATGTGTTTCTGACCACCCTGTCAAGCCTGATGCATGCAACAGCCCTGCTGGGTACAGCGGGTGTT
AGCGCAGCAGAAAGCATGCCGGAACTGATTGGTATGCTGCGTACCGTTCCTGCAATGCTGGAAGCGGG
TGGTGAAAATCCGGGTGCAGATATTGATGCAGATAAACATCCGGGTGATCTGAGCACCATTACCATGA
TGGGTGCAACCGCAGATCATATTGTTGGTGCAAGCGAAACCGCAGGTATTGATCTGGCACTGCCTCGT
GCAGTTCAGGCCCATTATCGTCGTGCAATTGAAAATGGTCATGGTGGTGATAATTGGACCCGTATTAT
TGATGGTATTCGTAGTCCGCGTtaaCTCGAGCCACTGAGATCCGGC [930 bp]

Protein Sequence incl. N-terminal His-tag:

MGSSHHHHHHSSGLVPRGSHMPPTDRTPVTLIGLGPMGQAMTRALLAAGHPVTVWNRTPARAAGVVAD
GAVLAASPVEAVEAGDLVILSLTDYQAMYDVLEPATGSLAGRTVVNLSSDTPDRTRAAADWATEHGAT
FLTGGVMIPAPMVGTEEAYVYYSGPAEVFEKHRTTLTVIGAPRYLGEDTGLAQLMYQAQLDVFLTTLS
SLMHATALLGTAGVSAAESMPELIGMLRTVPAMLEAGGENPGADIDADKHPGDLSTITMMGATADHIV
GASETAGIDLALPRAVQAHYRRAIENGHGGDNWTRIIDGIRSPR [316 aa]

Predicted Properties (*ExPASy* ProtParam):

Isoelectric point:	5.41
Molecular weight:	33,018 Da
Absorbance at 280 nm:	28,420 M^{-1} cm^{-1}

IRED-K (D2AWI4)

Native Gene Sequence (Genbank ID CP001814.1):

ATGAACACGAAGTCCGTGACTGTGATCGGCCTGGGTCCCATGGGGCAGGCGATGGCCGACGCCTACCT
GGACGGCGGCTACGAGGTGACCGTGTGGAACCGTACGGCCGCCCGGGCCGACCGGCTGGTGGCCCGCG
GCGCCCGGCGCGCGCCGACCGTCGAGGCCGCGCTGACCGCCAACGACCTGGTGGTGCTGAGCCTGACC
GACTATGACGCGATGTACGCCATCCTGGAGCAGGCCCCCTCCGCCGCCCTGGCCGGCCGTACCGTGGC
CAACCTCACCTCCGACACCCCCGAGAAGGCGCGTCAGGCGGCGGCGTGGTTGGCCGAGCGCGGCGCCG
TACAGATCACCGGTGGGGTCCAGGTGCCGCCCCCGGGGATCGGCAAGCCGGGGGCCACGACCTACTAC
AGCGGTCCCGAGGACGCGATCGAGGCCCACCGGCCCGCCCTGGAGGTGCTGACGGAGATCGACCACCT
CGGCGAAGACCCGGGGCTGGCGGCGCTGTTCTACCAGATCGGGATGGACATGTTCTGGACGGGCATCC
TGAGCTACGTGCACGCCCAGGCGGTGGCCGAAGCCAACGGCATCTCGGCGGAGCGGTTCCTCCCCAAC
GCCGTGAAGACCATGGACTTCCGCTACTTCCTGGAGTTCTACGCCCCGCGCATCGCCGCGGGCAACCA
CGAGGGGGACGTGGACCGGCTGGCCATGGGCGTGGCCAGCATGGAACACGTTCTGCACACTGTGGAGG
CGTCCGGCGTGGACGGCTCCCTGCCGGCGGCCGTCCTGGACGTCTTCCGCCGCGGCGTCGCCGCAGGG
CACGGCCAGGACAGCCTCACCAGCCTGATCAAGGTGCTGAAGCGGtga [864 bp]

Optimised Gene Sequence incl. 'Stuffer DNA' (*GeneArt*):

GTGCCGCGCGGCAGCCATATGAATACCAAAAGCGTTACCGTTATTGGTCTGGGTCCGATGGGTCAGGC
AATGGCAGATGCATATCTGGATGGTGGTTATGAAGTTACCGTTTGGAATCGTACCGCAGCACGTGCAG
ATCGTCTGGTTGCACGTGGTGCCCGTCGTGCACCGACCGTTGAAGCAGCACTGACCGCAAATGATCTG
GTTGTTCTGAGCCTGACCGATTATGATGCAATGTATGCAATTCTGGAACAGGCACCGAGCGCAGCACT
GGCAGGTCGTACCGTTGCAAATCTGACCAGCGATACACCGGAAAAAGCACGTCAGGCAGCAGCATGGC
TGGCAGAACGTGGTGCAGTTCAGATTACCGGTGGTGTTCAGGTTCCGCCTCCGGGTATTGGTAAACCG
GGTGCAACCACCTATTATAGCGGTCCGGAAGATGCAATTGAAGCACATCGTCCGGCACTGGAAGTTCT
GACCGAAATTGATCATCTGGGTGAAGATCCGGGTCTGGCAGCACTGTTTTATCAGATTGGTATGGATA
TGTTTTGGACCGGCATTCTGAGCTATGTTCATGCACAGGCAGTTGCCGAAGCAAATGGTATTAGCGCA
GAACGTTTTCTGCCGAATGCAGTTAAAACCATGGATTTCCGTTACTTTCTGGAATTTTATGCACCGCG
TATTGCAGCAGGCAATCATGAAGGTGATGTTGATCGCCTGGCAATGGGTGTTGCAAGCATGGAACATG
TGCTGCATACCGTGGAAGCAAGCGGTGTTGATGGTAGCCTGCCTGCAGCAGTTCTGGATGTTTTTCGT
CGTGGTGTTGCAGCCGGTCATGGTCAGGATTCACTGACCAGCCTGATTAAAGTTCTGAAACGCtaaCT
CGAGCCACTGAGATCCGGC [903 bp]

Protein Sequence incl. N-terminal His-tag:

MGSSHHHHHHSSGLVPRGSHMNTKSVTVIGLGPMGQAMADAYLDGGYEVTVWNRTAARADRLVARGAR
RAPTVEAALTANDLVVLSLTDYDAMYAILEQAPSAALAGRTVANLTSDTPEKARQAAAWLAERGAVQI
TGGVQVPPPGIGKPGATTYYSGPEDAIEAHRPALEVLTEIDHLGEDPGLAALFYQIGMDMFWTGILSY
VHAQAVAEANGISAERFLPNAVKTMDFRYFLEFYAPRIAAGNHEGDVDRLAMGVASMEHVLHTVEASG
VDGSLPAAVLDVFRRGVAAGHGQDSLTSLIKVLKR [307 aa]

Predicted Properties (*ExPASy* ProtParam):

Isoelectric point:	5.84
Molecular weight:	32,584 Da
Absorbance at 280 nm:	31,400 M^{-1} cm^{-1}

IRED-L (K0F8R0)

Native Gene Sequence (Genbank ID CP003876.1):

ATGTCCGAGCAGCACACCCCCCGTTCCGTTTCCGTCGTGGGCCTCGGGCCGATGGGCCAGTCCATGGT
CCGGGCCCTGCTCGACGCCGGCGTCGAGGTGACGGTCTGGAATCGCAGCACCGACAAGGTCGACGCGA
TGGTCGAACTCGGCGCCGTGCGGGCCGAGACGGTCGCGGCGGCGCTGGCGGCCAACGACGTGACGGTG
CTGAGCCTCACCCACTACGCGGCCATGTACAGCGTGCTCGAGCAGGCAGCGGATCAGCTGGCCGGCAA
GGTGATCGTCAACCTGTCCTCGGACTCCCCGGAGAAGGCGCGCAAGGGCGCCGAGTGGGTCCGTTCGC
ACGGCGCCGAATTCCTTTCCGGCGGTGTGATGTCGGCGGGTGACAACATCGCCCACCCGGCGTCGTAC
ATCTTCTACAGCGGTCCGCGCGAGGTCTTCGACGCGCATGCCGAGCTGCTGCGCCCGCTGAGCCCGCA
GGAGTACCTCGGCACGGACGACGGTCTGTCGCAGGTCTACTACCAGGCCTTGCTGACCATCTTCCATC
CGTGGCTGCTCGCCTTCGACCAGGCGACCGCGATGATCGAGCGGTCCGGCAACAGCATCGCGCAGTTC
ATCCCGTTCGCCGTGCGCTCCGCGGCCGCGTACCCCTACTTCATGGAGGAGTTCTCGGTCGCCAACCA
GAACGGCGGCTGGGCCACGCTGGCGAGCCTGAAAATGATGGACGCGGGCGCGCAGCACATCATCGATG
CCAGCGAAGAGGTCGGCGTCGACGCGACGTTCTCGCACACCGCACAGGCCTACTGGCGCAAGGCCGTC
GCGGCCAGCGAGGAGAAGGGCGAGGCCGTCTCCACCTACGCCCTGATGCGTGGCGCCGACGCGtga
[882 bp]

Optimised Gene Sequence incl. 'Stuffer DNA' (*GeneArt*):

GTGCCGCGCGGCAGCCATATGAGCGAACAGCATACACCGCGTAGCGTTAGCGTTGTTGGTCTGGGTCC
GATGGGTCAGAGCATGGTTCGTGCACTGCTGGATGCCGGTGTTGAAGTTACCGTTTGGAATCGTAGCA
CCGATAAAGTTGATGCAATGGTTGAACTGGGTGCAGTTCGTGCAGAAACCGTTGCAGCAGCACTGGCA
GCAAATGATGTTACCGTTCTGAGCCTGACCCATTATGCAGCAATGTATAGCGTTCTGGAACAGGCAGC
AGATCAGCTGGCAGGTAAAGTTATTGTTAATCTGAGCAGCGATAGTCCGGAAAAAGCACGTAAAGGTG
CAGAATGGGTTCGTAGCCATGGTGCCGAATTTCTGAGCGGTGGTGTTATGAGTGCCGGTGATAATATT
GCACATCCGGCAAGCTATATCTTTTATAGCGGTCCGCGTGAAGTTTTTGATGCACATGCAGAACTGCT
GCGTCCGCTGAGTCCGCAAGAATATCTGGGCACCGATGATGGTCTGAGCCAGGTTTATTATCAGGCAC
TGCTGACCATTTTTCATCCGTGGCTGCTGGCATTTGATCAGGCAACCGCAATGATTGAACGTAGCGGT
AATAGCATTGCACAGTTTATTCCGTTTGCCGTTCGTAGCGCAGCAGCATATCCGTATTTTATGGAAGA
ATTTAGCGTGGCCAATCAGAATGGTGGTTGGGCAACCCTGGCAAGCCTGAAAATGATGGATGCGGGTG
CACAGCATATTATTGATGCAAGCGAAGAGGTTGGTGTTGATGCCACCTTTAGCCATACCGCACAGGCA
TATTGGCGTAAAGCAGTTGCAGCCAGCGAAGAAAAAGGTGAAGCAGTTAGCACCTATGCACTGATGCG
TGGTGCAGATGCAtaaCTCGAGCCACTGAGATCCGGC [921 bp]

Protein Sequence incl. N-terminal His-tag:

MGSSHHHHHHSSGLVPRGSHMSEQHTPRSVSVVGLGPMGQSMVRALLDAGVEVTVWNRSTDKVDAMVE
LGAVRAETVAAALAANDVTVLSLTHYAAMYSVLEQAADQLAGKVIVNLSSDSPEKARKGAEWVRSHGA
EFLSGGVMSAGDNIAHPASYIFYSGPREVFDAHAELLRPLSPQEYLGTDDGLSQVYYQALLTIFHPWL
LAFDQATAMIERSGNSIAQFIPFAVRSAAAYPYFMEEFSVANQNGGWATLASLKMMDAGAQHIIDASE
EVGVDATFSHTAQAYWRKAVAASEEKGEAVSTYALMRGADA [313 aa]

Predicted Properties (*ExPASy* ProtParam):

Isoelectric point:	5.44
Molecular weight:	33,565 Da
Absorbance at 280 nm:	43,890 M^{-1} cm^{-1}

IRED-M (K0K4C6)

Native Gene Sequence (Genbank ID HE804045.1):

ATGAGCACGCCGCTGACGCTGATCGGCCTGGGCCCGATGGGGCAGGCCATGGTCGCGAAGTACCTGGA
GCACGGCCACCCGGTCACGGTGTGGAACCGGACCGCGAGCCGCGCCGACGACCTGGTGGCCCGGGGCG
CGGTGCGCGCCGACACCCCGCGTGACGCCGTGGCCGCCAACCGCCTGGTCGTGCTGAGCCTGACCGAC
TACCAGGCCATGTACGACGTCCTGGGCGACGCCGAACTGGCCGGGAAGACGGTCGTCAACCTCAGCTC
CGACACCCCCGACAAGACCCTCAAGGCCGCCGCGTGGCTCGCCGAACGGGGCGCGGAACTGGTGGTCG
GCGGCGTGATGGTGCCCGCGCCGCTGGTCGGCGAGGAAGCCGCGTACGTCTTCTACAGCGGCCCGAAG
GCCGTGTTCGAGCAGCACGCGGAGGTCCTGGCGGTGATCGGCCGCACCGAGTACCTCGGCGAGGACCA
CGCGCTGGCGCAGCTGTTCTACCAGGCCCAGCTGGACTTCTTCCTGACCACGCTGGCCGCCACGCTGC
ACAGCGTCGCCCTGGTCCGCACGGCCGGCGTGACCGCCGCGCAGTTCGCGCCGTACCTGAAGGACAAC
GCCGAGTCGATCTGGATGTACCTGGAGGAGACGATCACCGCCGTCGACCGCGGCGAGCACCCCGGCGA
CCTGGCCAACATCGTGATGATGGGCGCGACCGCCGACCACGTCGTCGGCGCGAGCGAGGCGACCGGGG
TGGACGCCGGGCTGCCCAGGGCCGTGCAGGACATGTACCGGCGGGCGATCGAGGCCGGGCACGGGCAG
GAGAGCTGGACCGCGCTCTACGAGGTGATCAAGCCCGCGAAGtag [861 bp]

Optimised Gene Sequence incl. 'Stuffer DNA' (*GeneArt*):

GTGCCGCGCGGCAGCCATATGAGCACACCGCTGACCCTGATTGGTCTGGGTCCGATGGGTCAGGCAAT
GGTTGCAAAATATCTGGAACATGGTCATCCGGTTACCGTTTGGAATCGTACCGCAAGCCGTGCAGATG
ATCTGGTTGCACGTGGTGCAGTTCGTGCAGATACACCGCGTGATGCAGTTGCAGCAAATCGTCTGGTT
GTTCTGAGCCTGACCGATTATCAGGCCATGTATGACGTTCTGGGTGATGCAGAACTGGCAGGTAAAAC
CGTTGTTAATCTGAGCAGCGATACACCGGATAAAACCCTGAAAGCAGCAGCATGGCTGGCAGAACGTG
GTGCCGAACTGGTTGTTGGTGGTGTTATGGTTCCGGCACCGCTGGTTGGTGAAGAGGCAGCCTATGTG
TTTTATAGCGGTCCGAAAGCAGTTTTTGAACAGCATGCAGAAGTTCTGGCAGTTATTGGTCGTACCGA
ATATCTGGGTGAAGATCATGCACTGGCACAGCTGTTTTATCAGGCGCAGCTGGATTTTTTTCTGACCA
CCCTGGCAGCAACCCTGCATAGCGTTGCACTGGTTCGTACAGCCGGTGTTACCGCAGCACAGTTTGCA
CCGTATCTGAAAGATAATGCAGAAAGCATTTGGATGTACCTGGAAGAAACCATTACCGCAGTTGATCG
TGGTGAACATCCGGGTGATCTGGCAAATATTGTTATGATGGGTGCAACCGCAGATCATGTTGTGGGTG
CAAGCGAAGCAACCGGTGTTGATGCAGGTCTGCCTCGTGCAGTTCAGGATATGTATCGTCGTGCAATT
GAAGCAGGTCATGGTCAAGAAAGCTGGACCGCACTGTATGAAGTTATTAAACCGGCAAAAtaaCTCGA
GCCACTGAGATCCGGC [900 bp]

Protein Sequence incl. N-terminal His-tag:

MGSSHHHHHHSSGLVPRGSHMSTPLTLIGLGPMGQAMVAKYLEHGHPVTVWNRTASRADDLVARGAVR
ADTPRDAVAANRLVVLSLTDYQAMYDVLGDAELAGKTVVNLSSDTPDKTLKAAAWLAERGAELVVGGV
MVPAPLVGEEAAYVFYSGPKAVFEQHAEVLAVIGRTEYLGEDHALAQLFYQAQLDFFLTTLAATLHSV
ALVRTAGVTAAQFAPYLKDNAESIWMYLEETITAVDRGEHPGDLANIVMMGATADHVVGASEATGVDA
GLPRAVQDMYRRAIEAGHGQESWTALYEVIKPAK [306 aa]

Predicted Properties (*ExPASy* ProtParam):

Isoelectric point:	5.53
Molecular weight:	32,701 Da
Absorbance at 280 nm:	38,390 M^{-1} cm^{-1}

IRED-N (J7YM26)

Native Gene Sequence (Genbank ID AHDE01000056.1):

ATGAAAAGTAATAGCCAAAATGAGAAAAATGGTTCTGAAACTACAAACGCGGTTGGGAACCGAAAATC
AGTAACAGTCATAGGATTAGGGCCGATGGGTCAGGCGATGGCAGACGTTTTTCTGGAATACGGGTACT
CGGTGACCGTGTGGAACCGGACTTCAAGTAAAGCAGACCAGCTTGTAGCAAAAGGAGCCATCAGGGTG
TCCACGGTCAACGAGGCGTTAGCTGCTAACGAGTTGGTAATCCTTAGCCTTACAGATTACAATGTGAT
GTACTCTATTCTTGAACCCGTCTCTGAGAACTTATTCGGTAAGGTGCTCGTTAACTTGAGTTCGGATA
CTCCGGAGAAAGCTCGTAAGGCGGCTAAATGGTTGGAAGACCGTGGAGCCCGGCATATTACTGGAGGG
GTGCAAGTTCCACCGTCAGGCATAGGTAAATCGGAATCTTACACATACTATAGTGGTGATCGAGTGGT
CTTCGAGGCTCACAGGGAGACCTTGGAAGTGTTAACAAGTAGCGATTACCGGGGCGAGGACCCCGGAT
TAGCCATGCTATACTATCAGATACAGATGGATATATTCTGGACGGCAATGCTGAGTTACCTACATGCT
CTTGCAATCGCTAACGCAAACGGCATTACGGCGGAGCAATTCCTACCATACGCTTCGGCGATGATGTC
GTCACTGCCGAAATTCGTAGAGTTTTATACCCCGCGTCTCGACGAGGGTGAGCACCCTGGTGACGTGG
ACAGACTTGCAATGGGCTTGGCGAGCGTTGAGCACGTTGTTCATACTACCCAAGAAGCCGGCATCGAC
ATCGCTTTGCCAGCAACCGTTTTGGAAGTCTTCAGGCGCGGCATGAAGACTGGTCATGCCAGTGATAG
CTTCACCAGCCTAATTGAAATCTTCAAAAATTCCGATATTCGTTCAtaa [933 bp]

Optimised Gene Sequence incl. 'Stuffer DNA' (*GeneArt*):

GTGCCGCGCGGCAGCCATATGAAAAGCAATAGCCAGAATGAAAAAAACGGTAGCGAAACCACCAATGC
AGTTGGTAATCGTAAAAGCGTTACCGTTATTGGTCTGGGTCCGATGGGTCAGGCAATGGCAGATGTTT
TTCTGGAATATGGTTATAGCGTGACCGTTTGGAATCGTACCAGCAGCAAAGCAGATCAGCTGGTTGCA
AAAGGTGCAATTCGTGTTAGCACCGTTAATGAAGCACTGGCAGCAAATGAACTGGTTATTCTGAGCCT
GACCGATTACAATGTGATGTATAGCATTCTGGAACCGGTTAGCGAAAACCTGTTTGGTAAAGTTCTGG
TGAATCTGAGCAGCGATACACCGGAAAAAGCACGTAAAGCAGCAAAATGGCTGGAAGATCGTGGTGCA
CGTCATATTACCGGTGGTGTTCAGGTTCCGCCTAGCGGTATTGGTAAAAGCGAAAGCTATACCTATTA
TAGCGGTGATCGTGTTGTTTTTGAAGCACATCGTGAAACCCTGGAAGTTCTGACCAGCAGCGATTATC
GTGGTGAAGATCCGGGTCTGGCAATGCTGTATTATCAGATTCAGATGGATATCTTTTGGACCGCAATG
CTGAGCTATCTGCATGCACTGGCAATTGCAAATGCAAATGGTATTACCGCAGAACAGTTTCTGCCGTA
TGCAAGCGCAATGATGAGCAGCCTGCCGAAATTTGTTGAATTCTATACACCGCGTCTGGATGAAGGCG
AACATCCGGGTGATGTTGATCGTCTGGCCATGGGTCTGGCCAGCGTTGAACATGTTGTTCATACCACC
CAAGAAGCCGGTATTGATATTGCACTGCCTGCAACCGTTCTGGAAGTTTTTCGTCGCGGTATGAAAAC
CGGTCATGCAAGCGATAGCTTTACCAGCCTGATTGAGATTTTCAAAAATAGCGATATCCGCAGCtaaC
TCGAGCCACTGAGATCCGGC [972 bp]

Protein Sequence incl. N-terminal His-tag:

MGSSHHHHHHSSGLVPRGSHMKSNSQNEKNGSETTNAVGNRKSVTVIGLGPMGQAMADVFLEYGYSVT
VWNRTSSKADQLVAKGAIRVSTVNEALAANELVILSLTDYNVMYSILEPVSENLFGKVLVNLSSDTPE
KARKAAKWLEDRGARHITGGVQVPPSGIGKSESYTYYSGDRVVFEAHRETLEVLTSSDYRGEDPGLAM
LYYQIQMDIFWTAMLSYLHALAIANANGITAEQFLPYASAMMSSLPKFVEFYTPRLDEGEHPGDVDRL
AMGLASVEHVVHTTQEAGIDIALPATVLEVFRRGMKTGHASDSFTSLIEIFKNSDIRS [330 aa]

Predicted Properties (*ExPASy* ProtParam):

Isoelectric point:	5.86
Molecular weight:	36,098 Da
Absorbance at 280 nm:	35,870 M^{-1} cm^{-1}

Pairwise Protein Sequence Comparison

IRED	A	B	C	D	E	F	G	H	I	J	K	L	M	N
**A** (M4ZRJ3)		49.8	33.2	35.6	32.4	38.7	38.4	45.4	35.8	37.5	34.1	31.5	40.3	33.8
**B** (Q1EQE0)	0.7		37.5	40.2	35.8	42.7	46.0	45.7	34.6	38.0	36.6	29.1	37.5	32.6
**C** (W7VJL8)	1.1	0.9		45.3	44.3	41.1	40.3	36.1	31.9	36.5	33.8	28.5	35.3	30.0
**D** (V7GV82)	1.0	0.9	0.8		37.3	42.8	37.7	39.0	36.4	32.6	35.7	29.2	31.2	31.4
**E** (J7LAY5)	1.1	1.0	0.8	1.0		38.0	37.3	35.0	31.0	33.3	31.2	29.0	32.5	28.2
**F** (V6KA13)	1.0	0.8	0.9	0.8	1.0		42.0	41.9	31.9	33.6	35.0	29.2	31.1	30.6
**G** (L8EIW6)	1.0	0.8	0.9	1.0	1.0	0.9		39.7	36.2	36.6	32.9	33.0	36.0	31.5
**H** (I8QLV7)	0.8	0.7	1.0	0.9	1.0	0.9	0.9		39.0	38.4	39.3	33.1	35.8	35.8
**I** (M4ZS15)	1.0	1.0	1.2	1.0	1.2	1.2	1.0	0.9		43.8	57.0	37.8	44.8	56.8
**J** (D2PR38)	1.0	0.9	1.0	1.1	1.1	1.1	1.0	0.9	0.8		43.8	37.6	57.8	40.8
**K** (D2AWI4)	1.1	1.0	1.1	1.0	1.2	1.1	1.1	0.9	0.6	0.8		38.3	48.3	58.2
**L** (K0F8R0)	1.2	1.2	1.3	1.3	1.3	1.3	1.1	1.1	1.0	1.0	1.0		40.8	36.1
**M** K0K4C6)	0.9	0.9	1.1	1.2	1.1	1.2	1.0	1.0	0.8	0.5	0.7	0.9		43.2
**N** J7YM26)	1.1	1.2	1.2	1.1	1.3	1.2	1.2	1.0	0.5	0.9	0.5	1.0	0.8	

Supplementary Table S7. Pairwise protein sequence identities (above diagonal) and Jukes–Cantor-corrected phylogenetic distances (below diagonal) of IREDs **A**–**N**. The comparison is based on a Clustal Omega alignment of the native protein sequences.

NMR and MS Spectra

(*S*)-1-(2-Methylpiperidin-1-yl)ethanone

- ^{1}H-NMR spectrum (Supplementary Figure S39)
- ^{13}C-NMR spectrum (Supplementary Figure S40)
- DEPT90-NMR spectrum (Supplementary Figure S41)
- DEPT135-NMR spectrum (Supplementary Figure S42)
- COSY-NMR spectrum (Supplementary Figure S43)
- HSQC-NMR spectrum (Supplementary Figure S44)
- MS spectrum (Supplementary Figure S45)
- Chiral-phase GC chromatogram (Supplementary Figure S46)

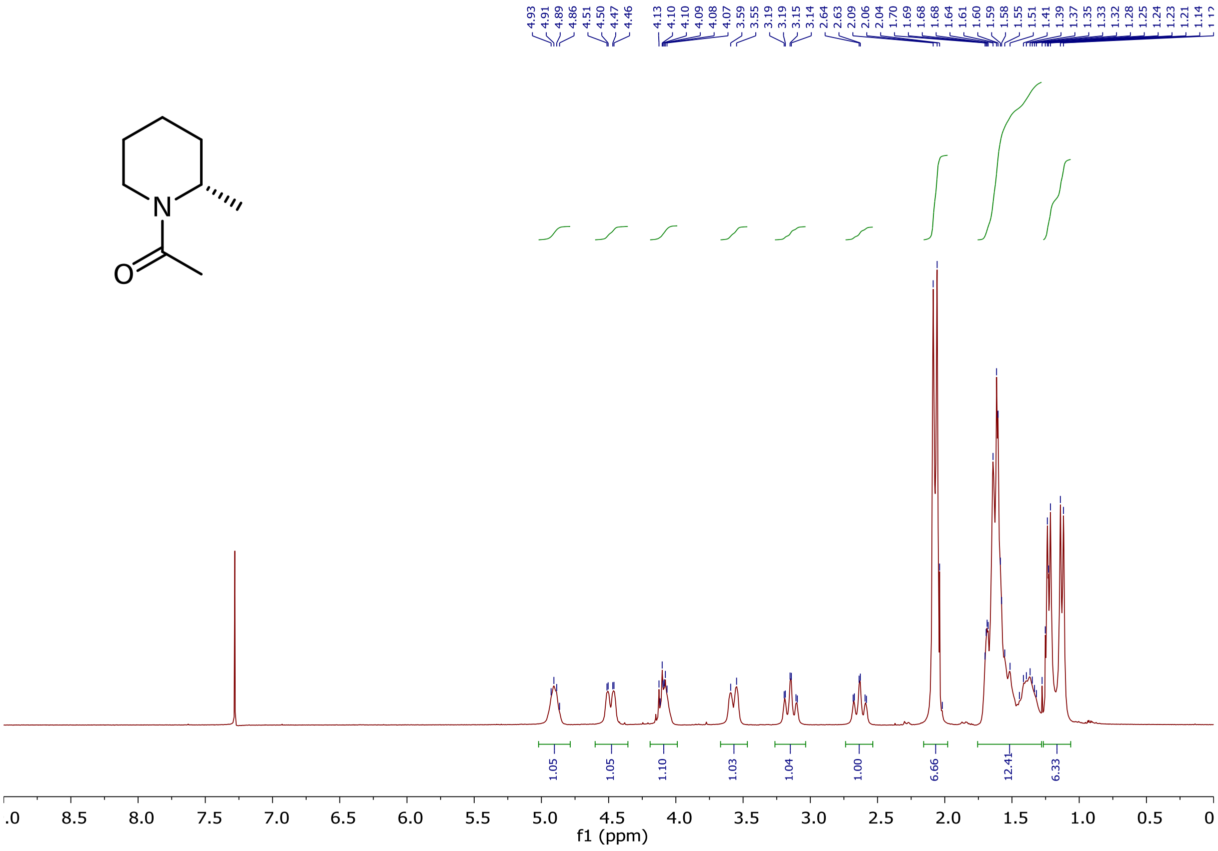

Supplementary Figure S39. ^{1}H-NMR spectrum of (*S*)-1-(2-methylpiperidin-1-yl)ethanone.

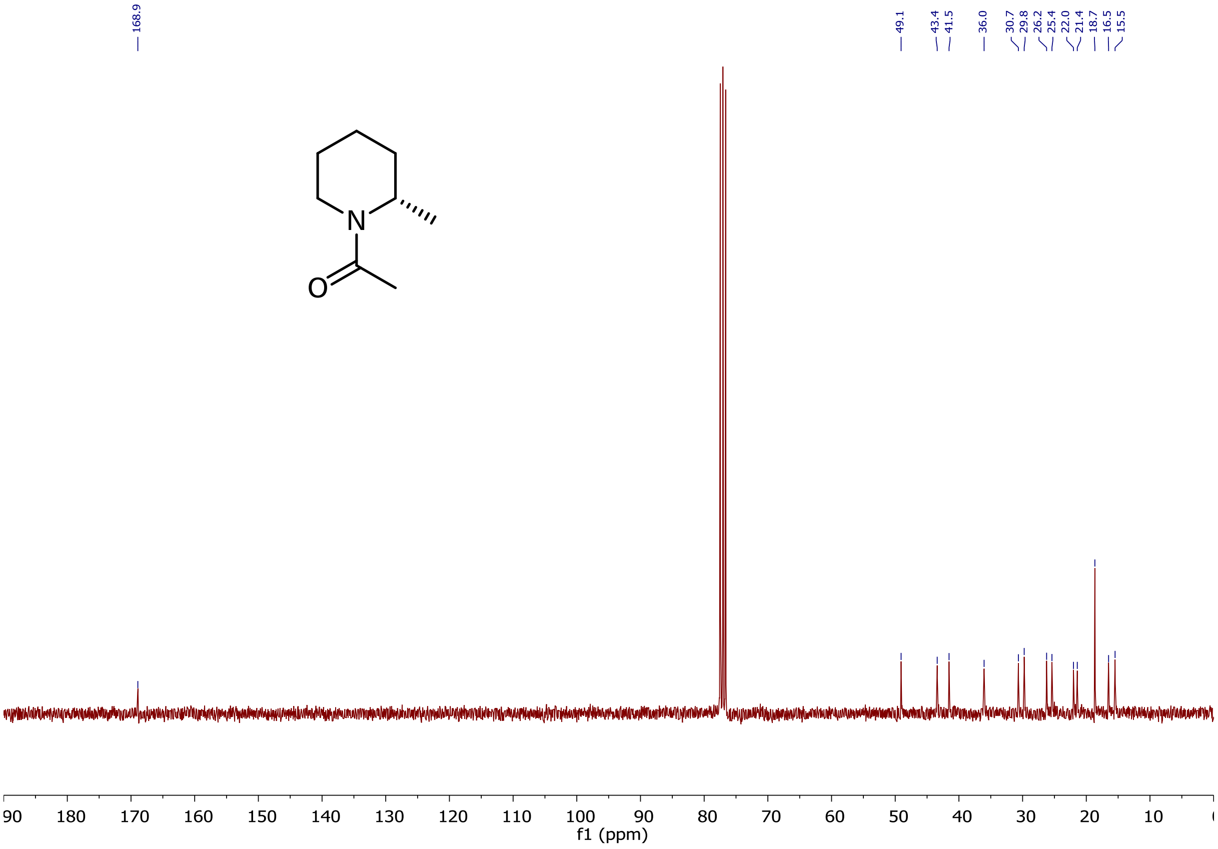

Supplementary Figure S40. ^{13}C-NMR spectrum of (*S*)-1-(2-methylpiperidin-1-yl)ethanone.

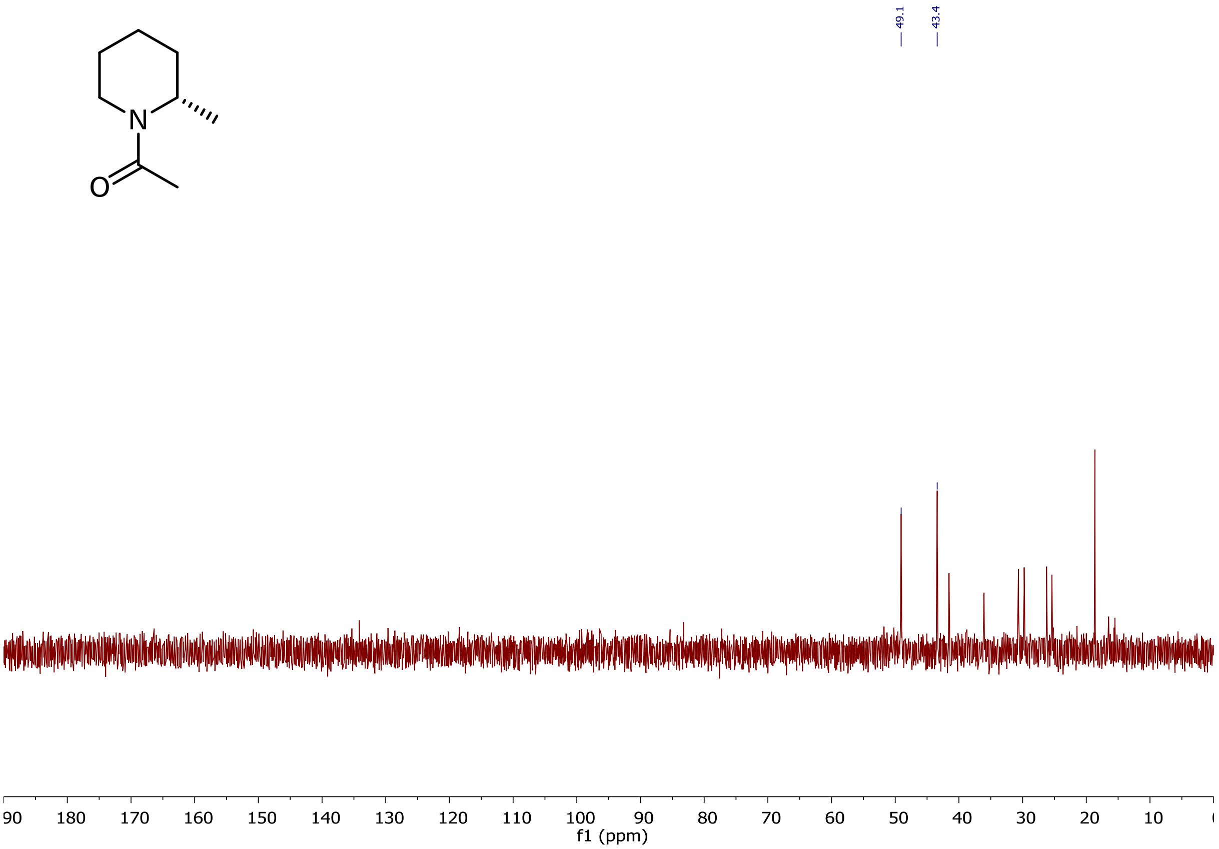

Supplementary Figure S41. DEPT90-NMR spectrum of (*S*)-1-(2-methylpiperidin-1-yl)ethanone.

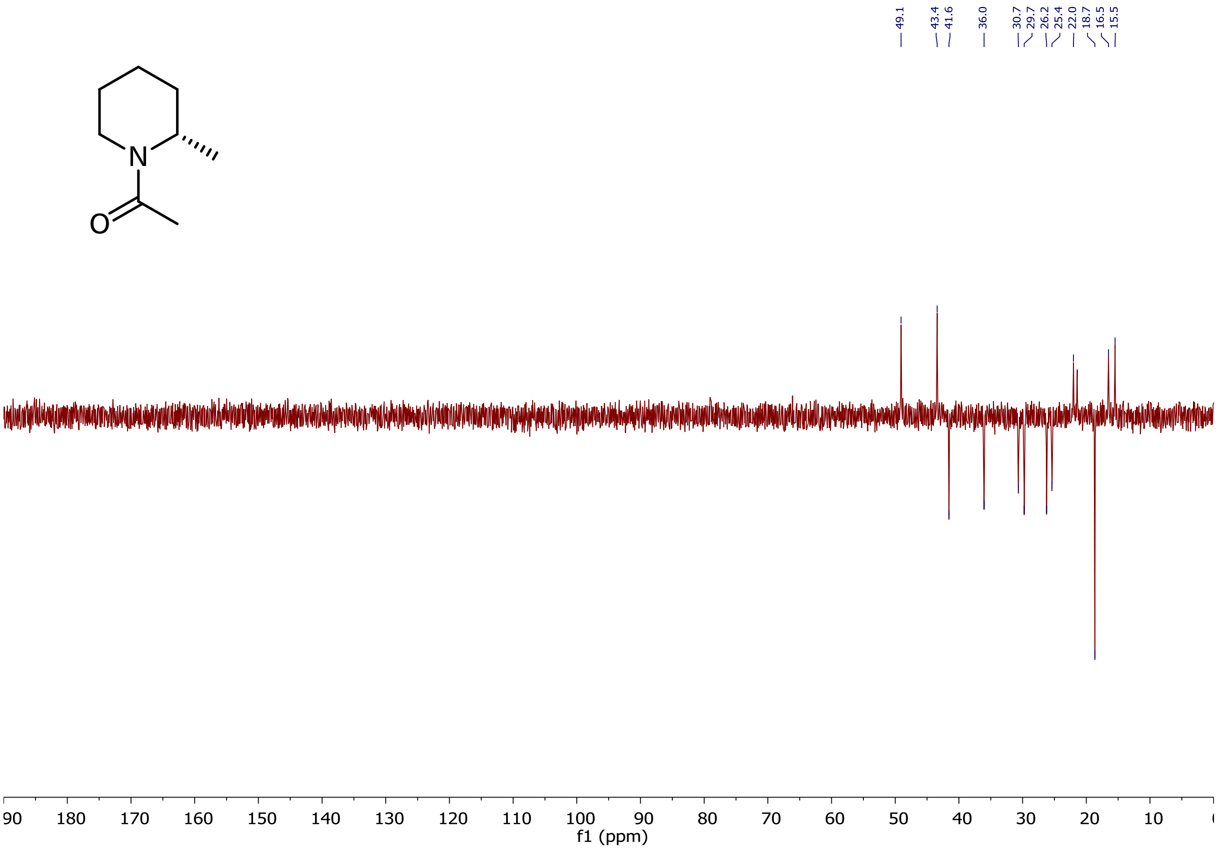

Supplementary Figure S42. DEPT135-NMR spectrum of (*S*)-1-(2-methylpiperidin-1-yl)ethanone.

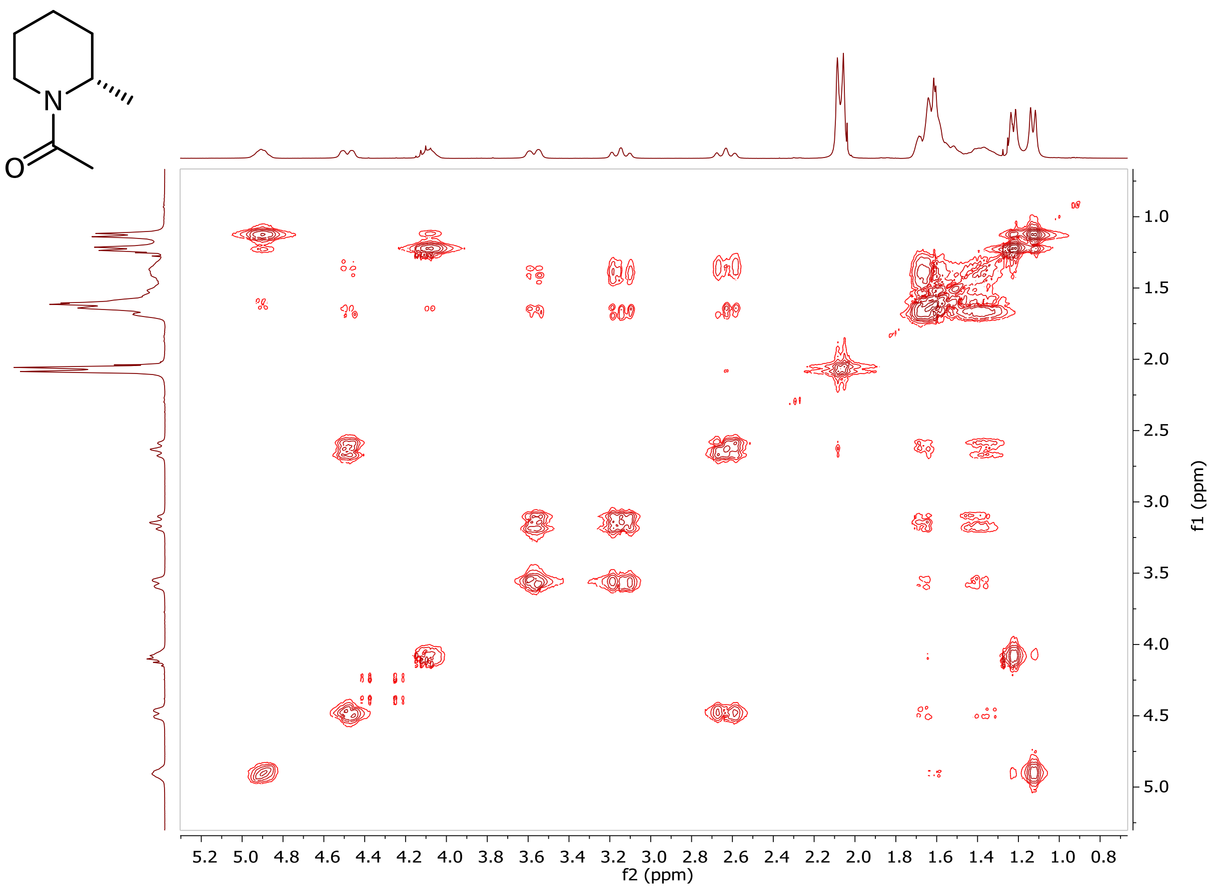

Supplementary Figure S43. COSY-NMR spectrum of (*S*)-1-(2-methylpiperidin-1-yl)ethanone.

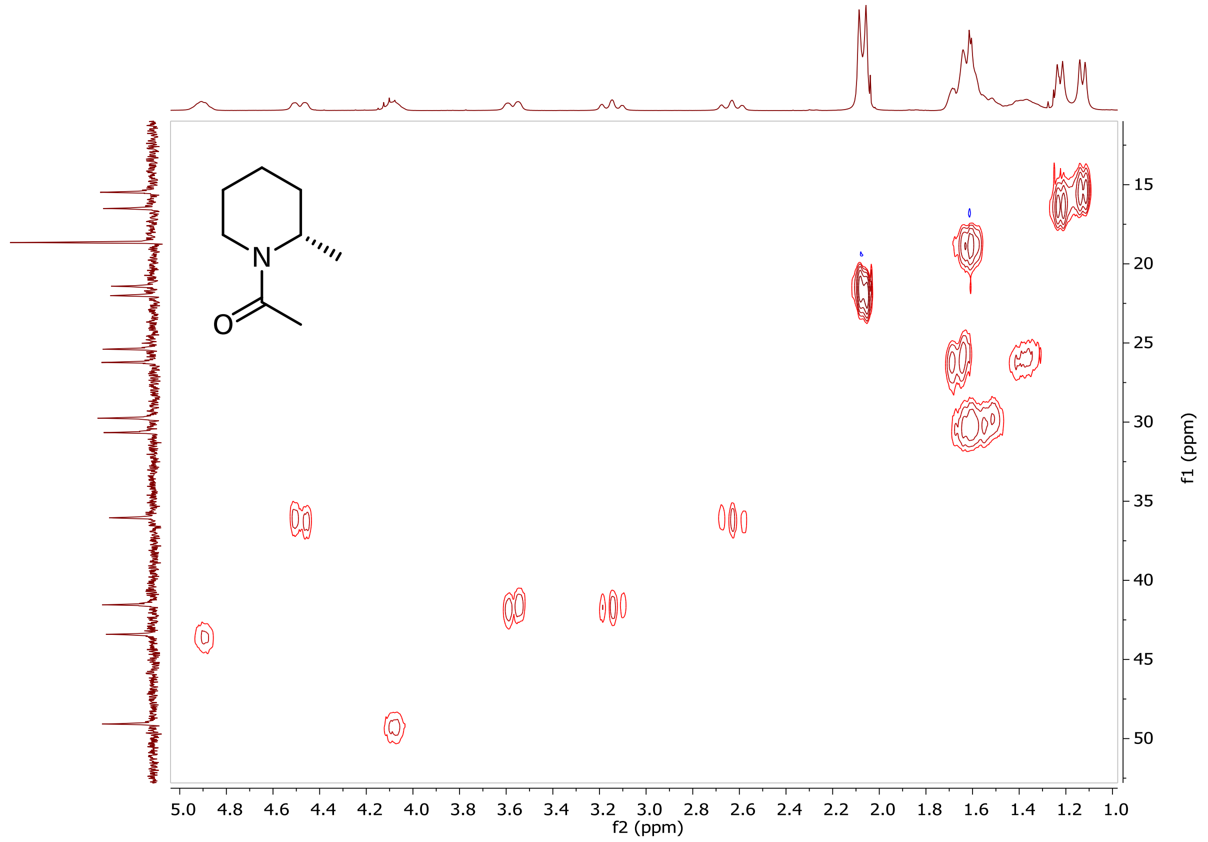

Supplementary Figure S44. HSQC-NMR spectrum of (*S*)-1-(2-methylpiperidin-1-yl)ethanone.

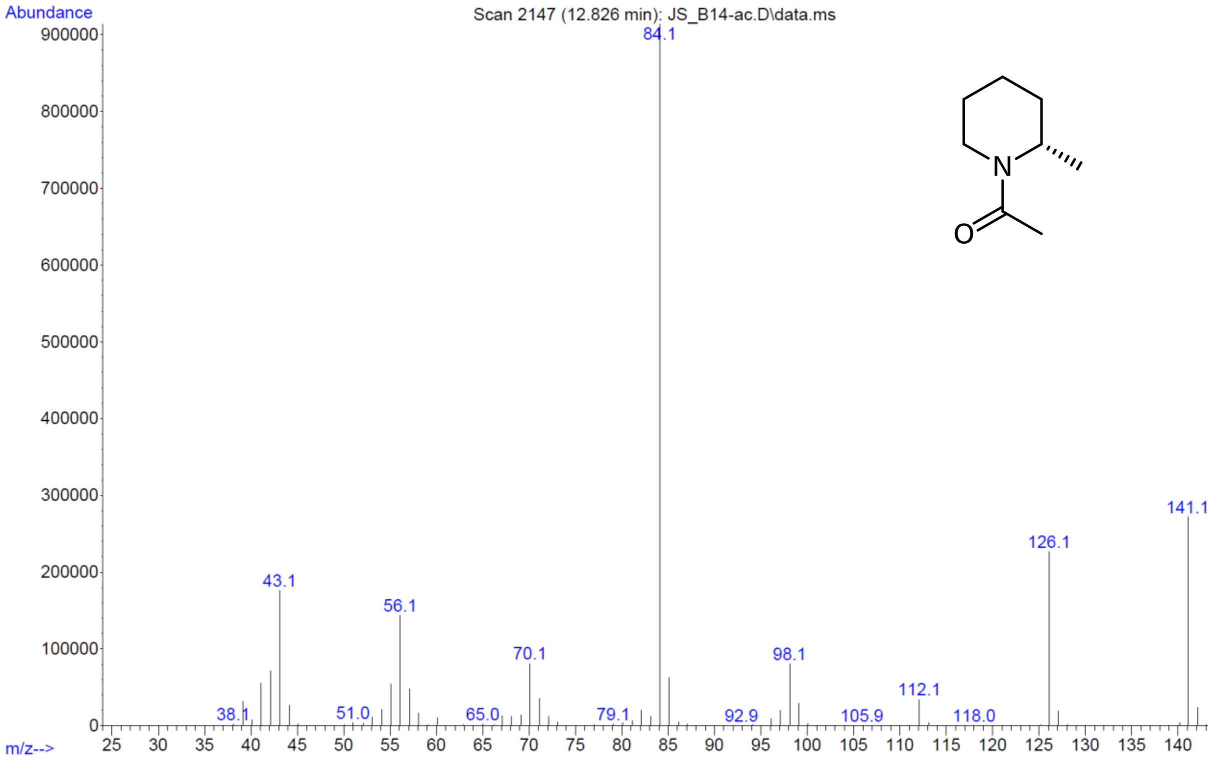

Supplementary Figure S45. MS spectrum of (*S*)-1-(2-methylpiperidin-1-yl)ethanone.

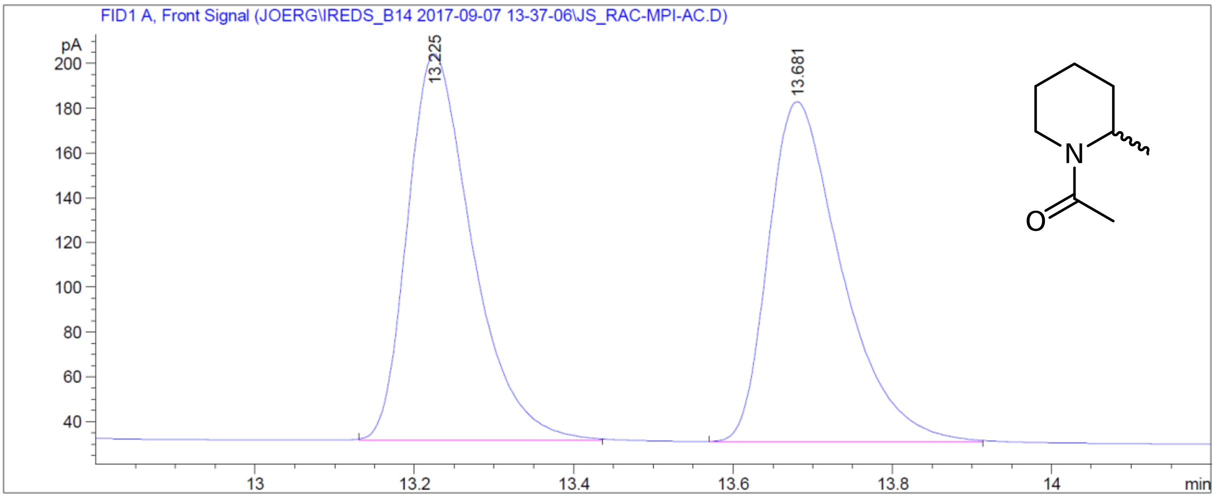

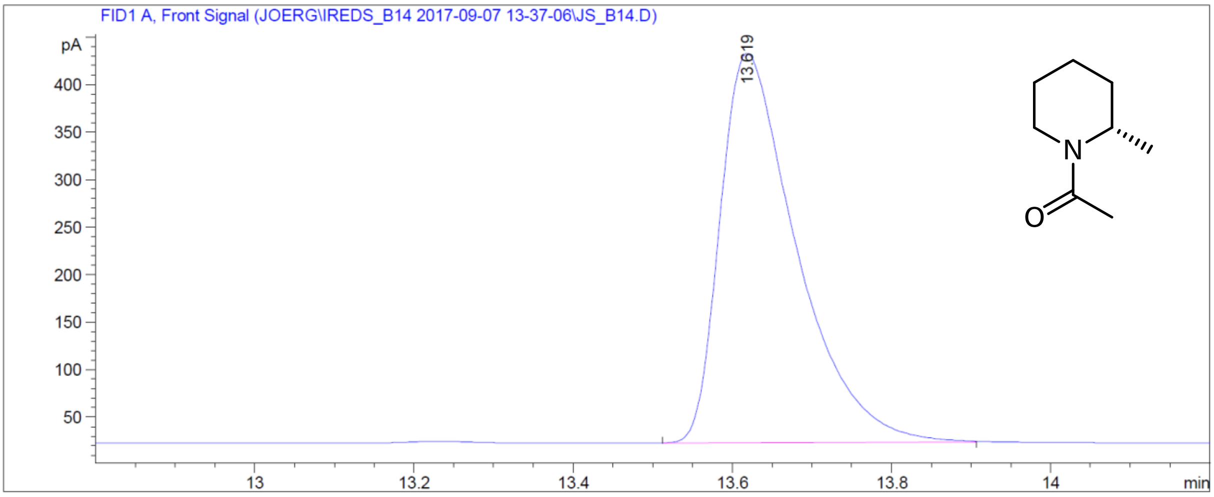

Supplementary Figure S46. Determination of the enantiomeric excess of (*S*)-1-(2-methylpiperidin-1-yl)ethanone using GC method GC-C1. Racemic standard (top), biotransformation product (bottom).

(*R*)-1-Methyl-1,2,3,4-tetrahydroisoquinoline (*R*)-2g

- ^{1}H-NMR spectrum (Supplementary Figure S47)
- ^{13}C-NMR spectrum (Supplementary Figure S48)
- MS spectrum (Supplementary Figure S49)
- Chiral-phase HPLC chromatogram (Supplementary Figure S50)

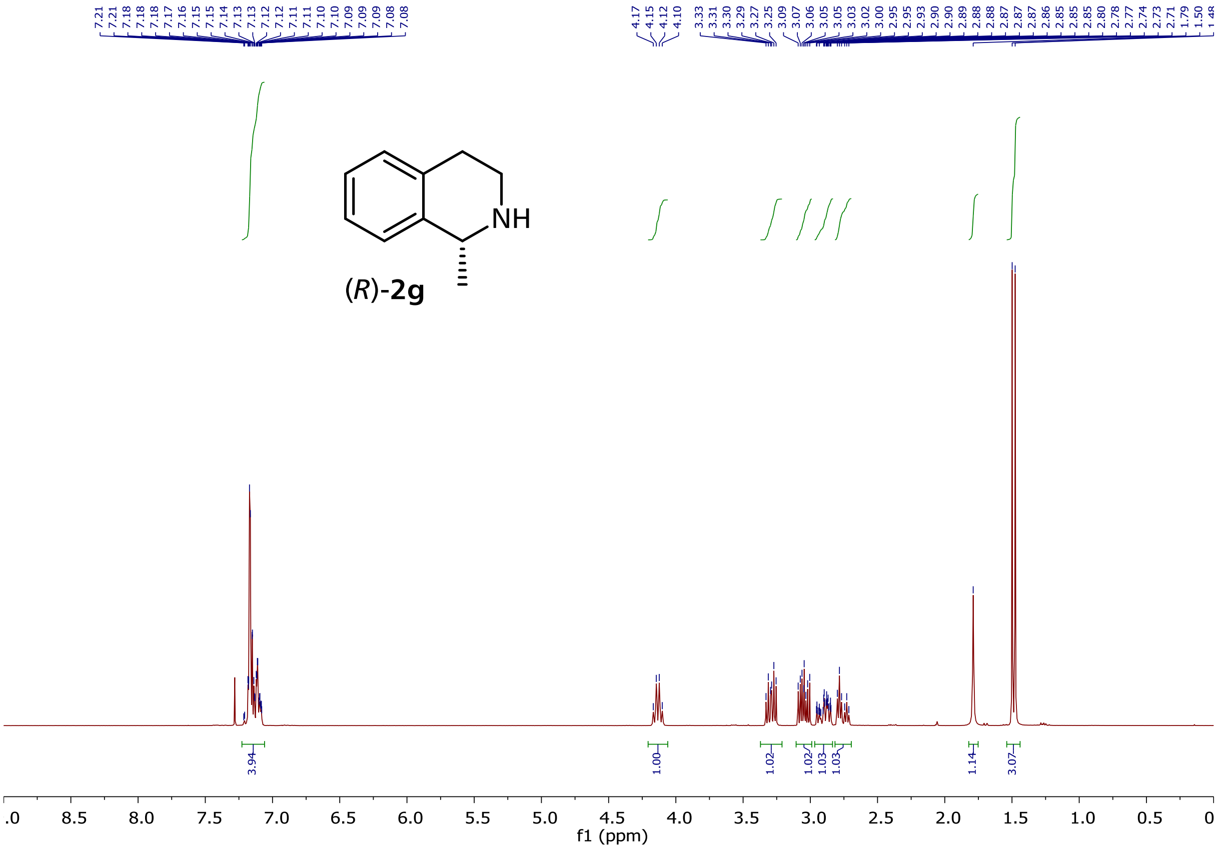

Supplementary Figure S47. ^{1}H-NMR spectrum of (*R*)-**2g**.

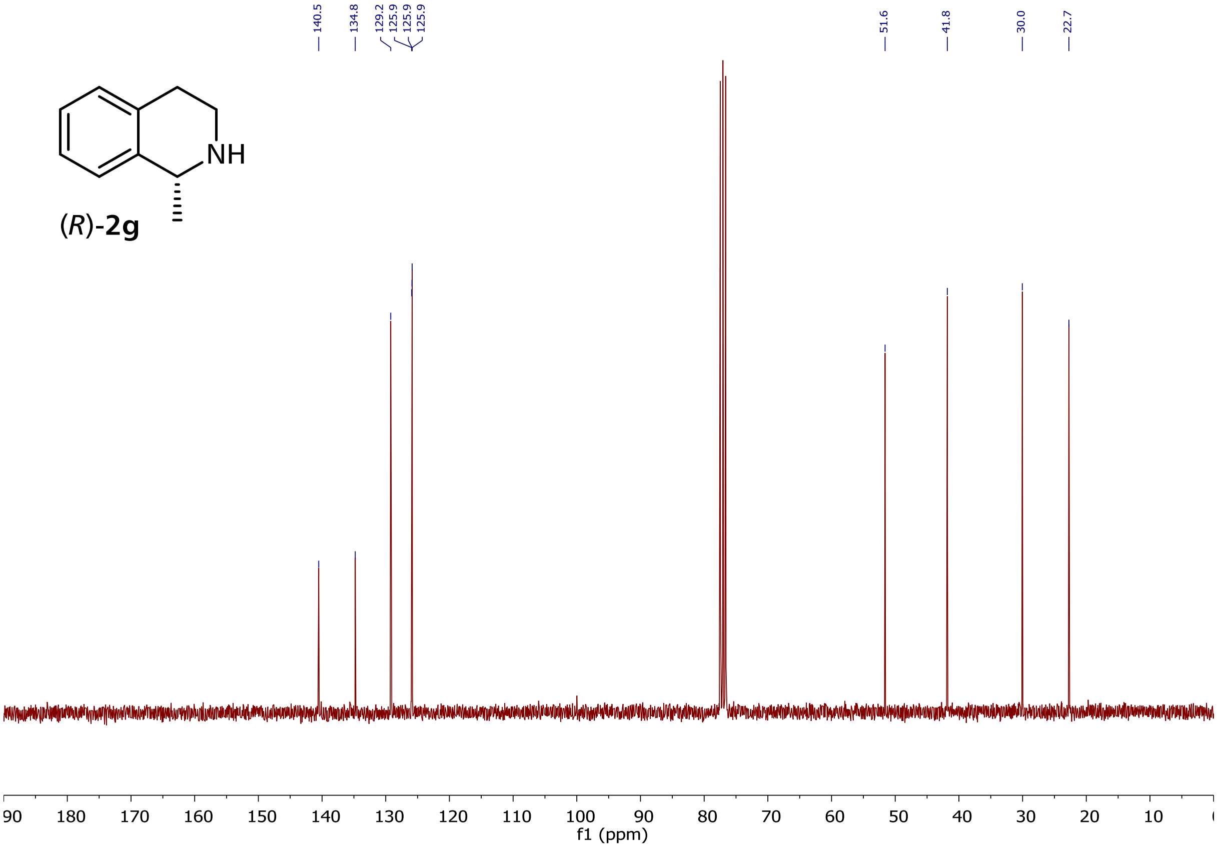

Supplementary Figure S48. ^{13}C-NMR spectrum of (*R*)-**2g**.

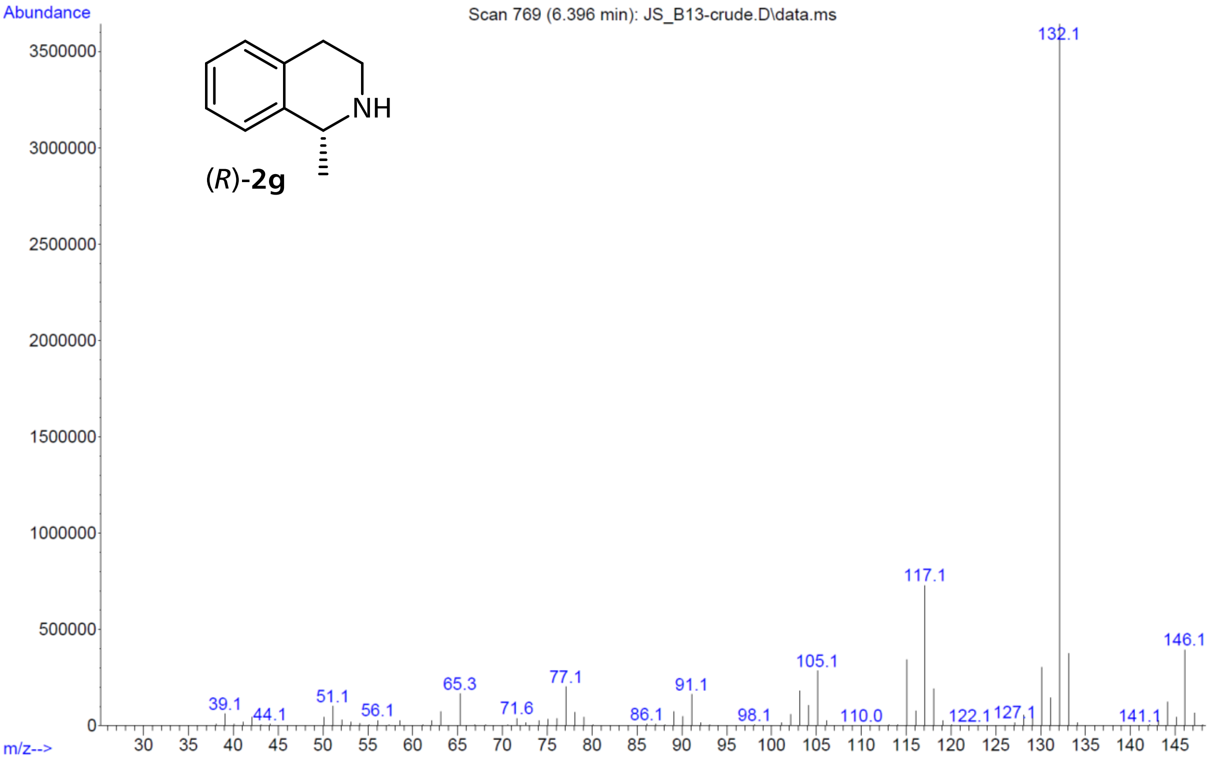

Supplementary Figure S49. MS spectrum of (*R*)-**2g**.

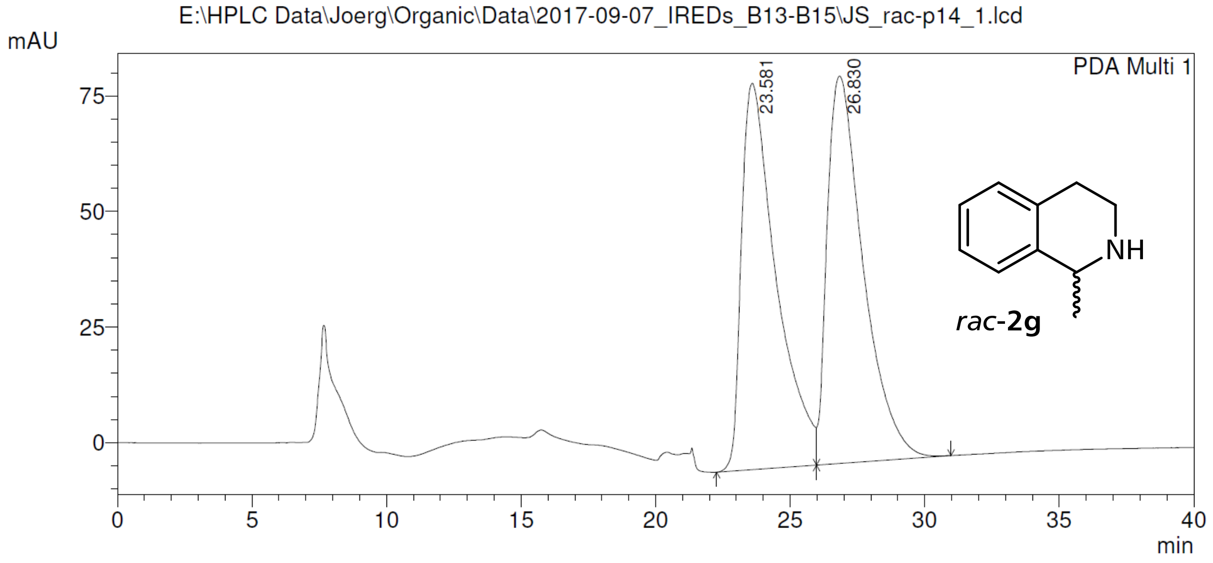

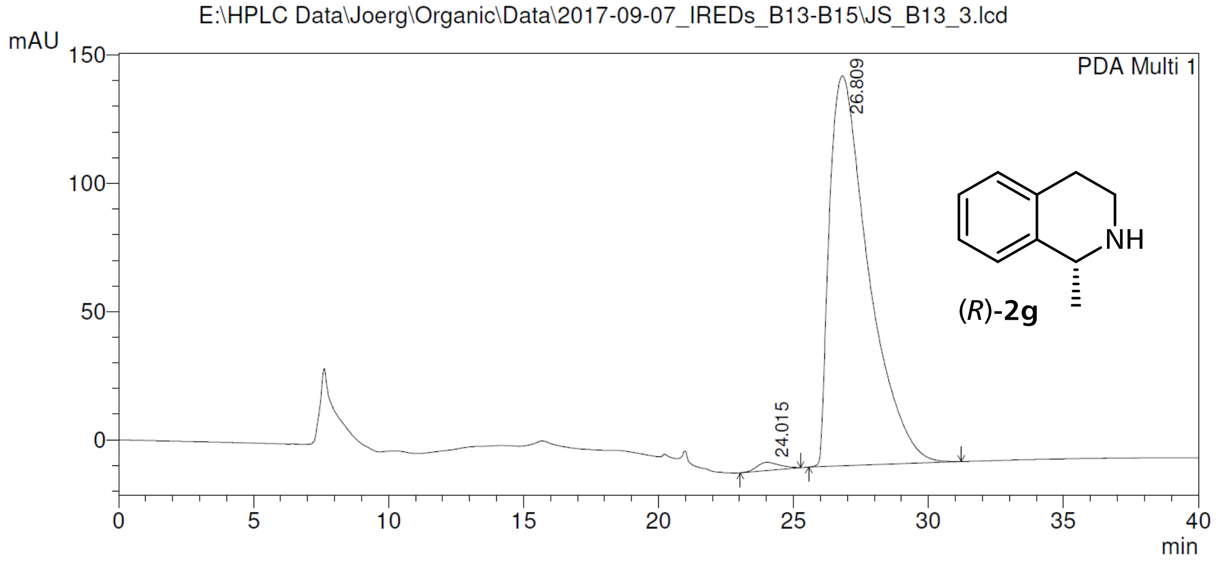

Supplementary Figure S50. Determination of the enantiomeric excess of (*R*)-**2g** using HPLC method HPLC-C1. Racemic standard (top), biotransformation product (bottom).

(*R*)-1-(2-Methylazepan-1-yl)ethanone

- ^{1}H-NMR spectrum (Supplementary Figure S51)
- ^{13}C-NMR spectrum (Supplementary Figure S52)
- DEPT90-NMR spectrum (Supplementary Figure S53)
- DEPT135-NMR spectrum (Supplementary Figure S54)
- COSY-NMR spectrum (Supplementary Figure S55)
- HSQC-NMR spectrum (Supplementary Figure S56)
- MS spectrum (Supplementary Figure S57)
- Chiral-phase GC chromatogram (Supplementary Figure S58)

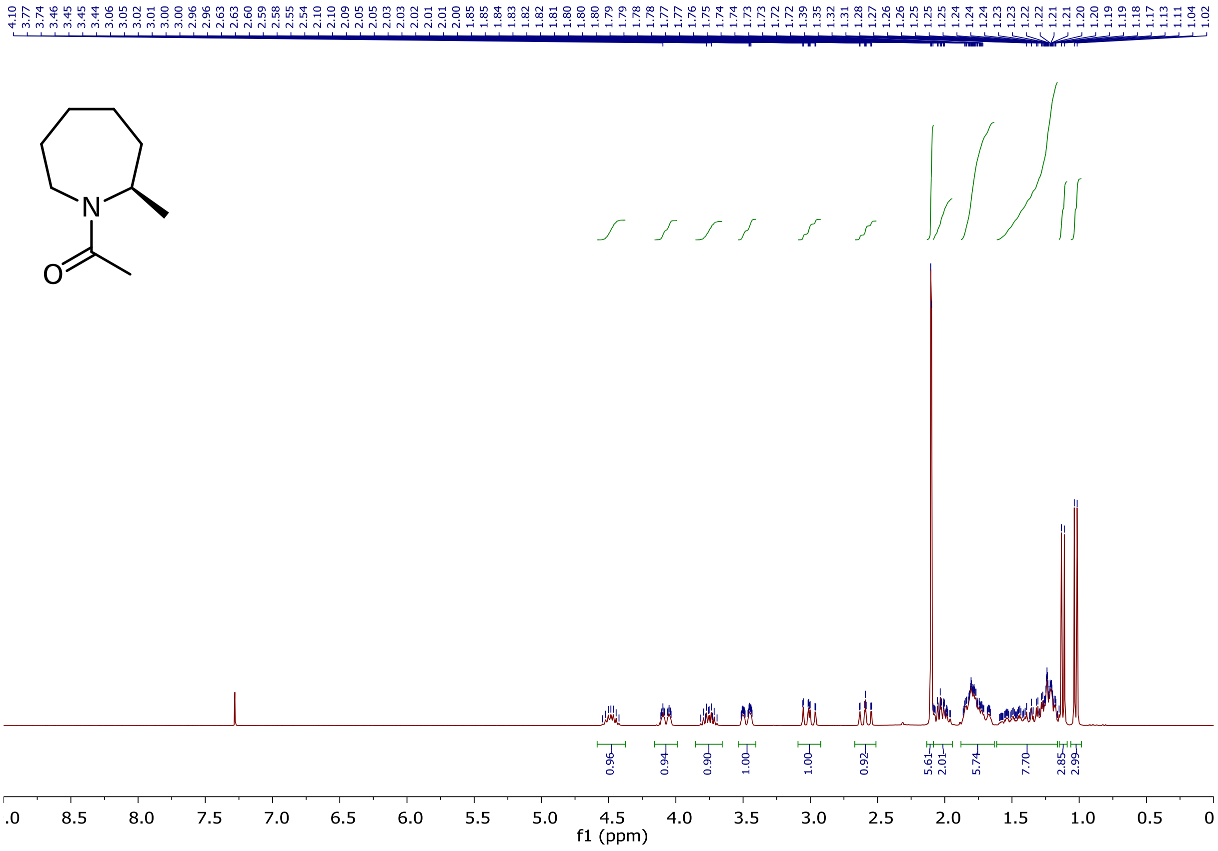

Supplementary Figure S51. ^{1}H-NMR spectrum of (*R*)-1-(2-methylazepan-1-yl)ethanone.

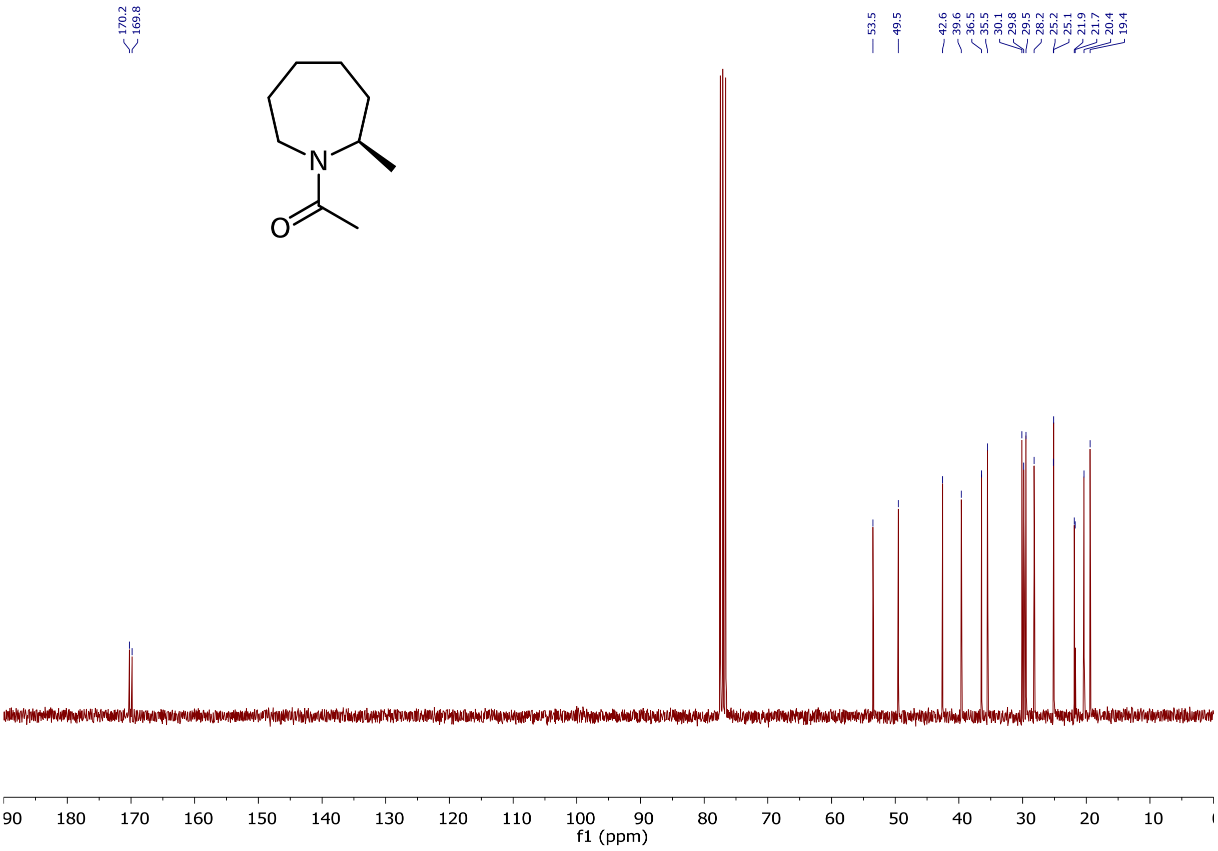

Supplementary Figure S52. ^{13}C-NMR spectrum of (*R*)-1-(2-methylazepan-1-yl)ethanone.

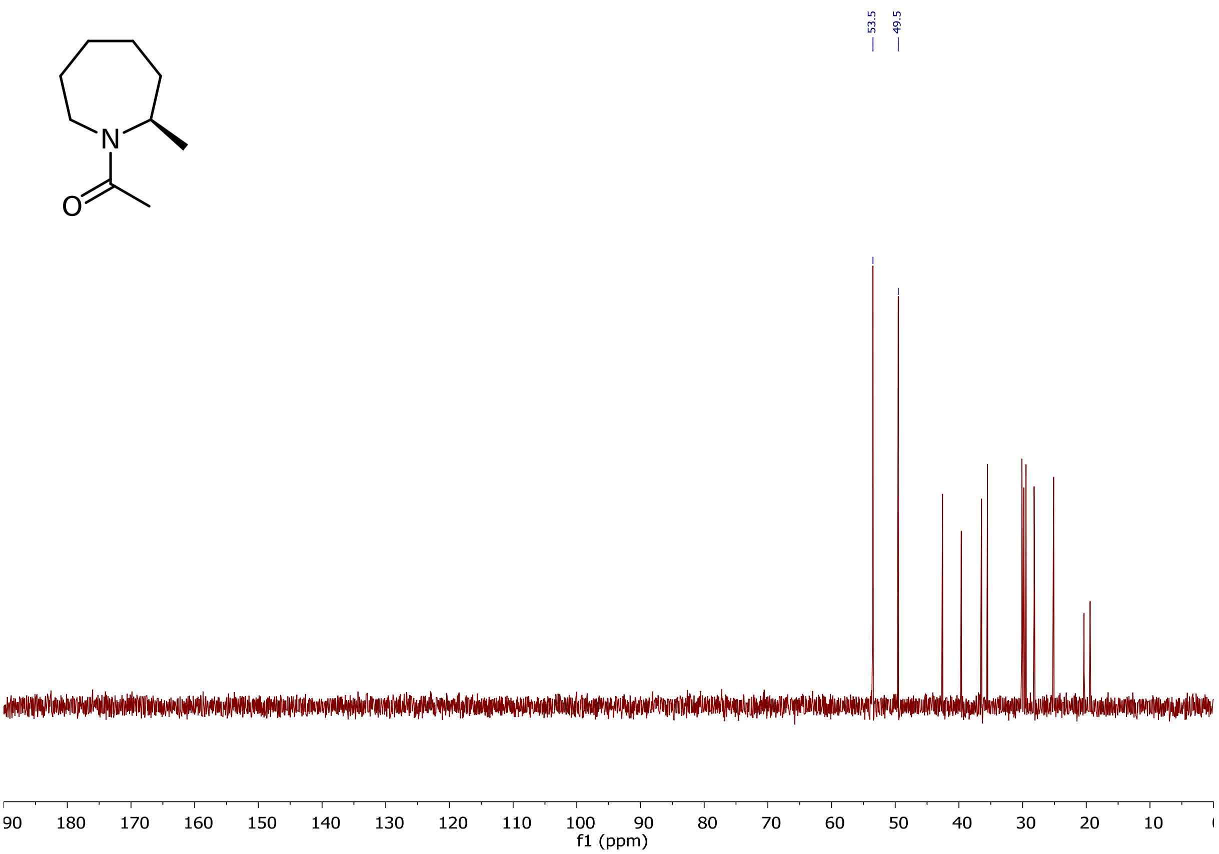

Supplementary Figure S53. DEPT90-NMR spectrum of (*R*)-1-(2-methylazepan-1-yl)ethanone.

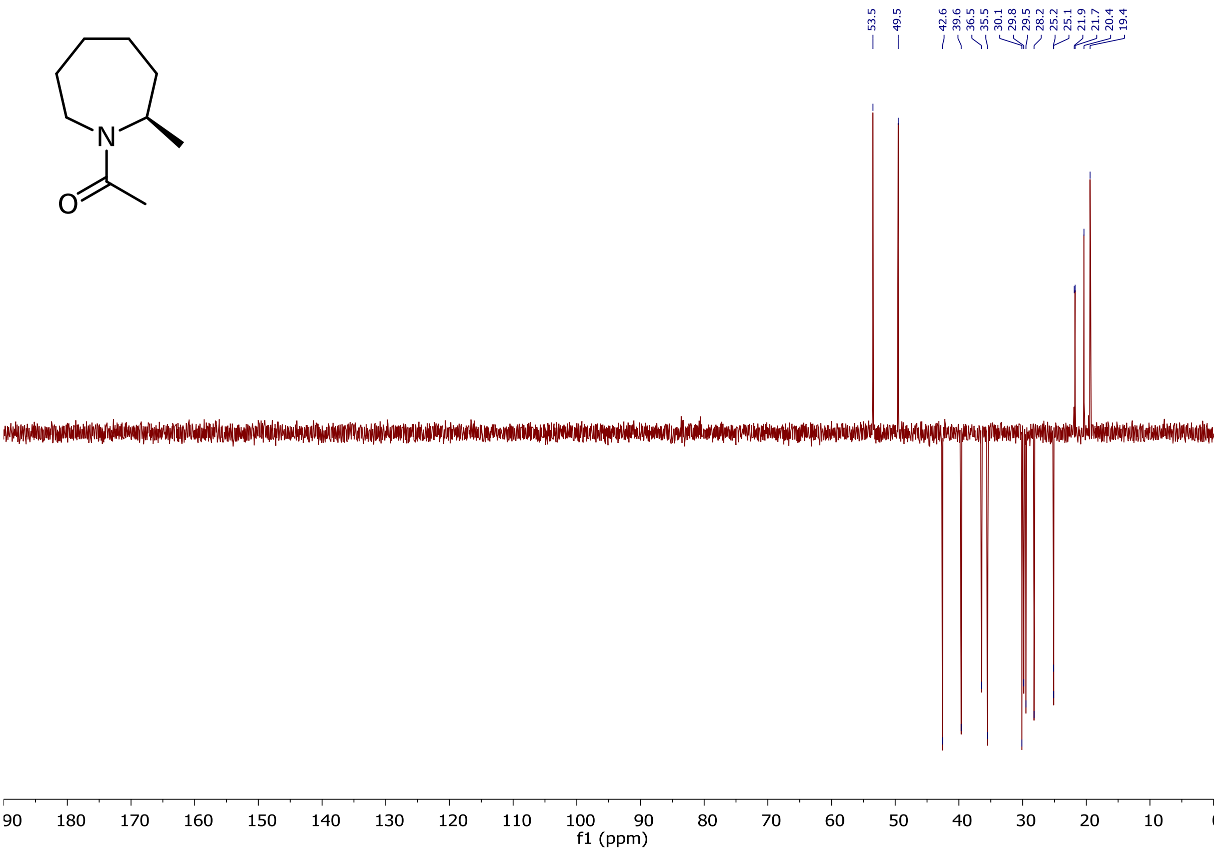

Supplementary Figure S54. DEPT135-NMR spectrum of (*R*)-1-(2-methylazepan-1-yl)ethanone.

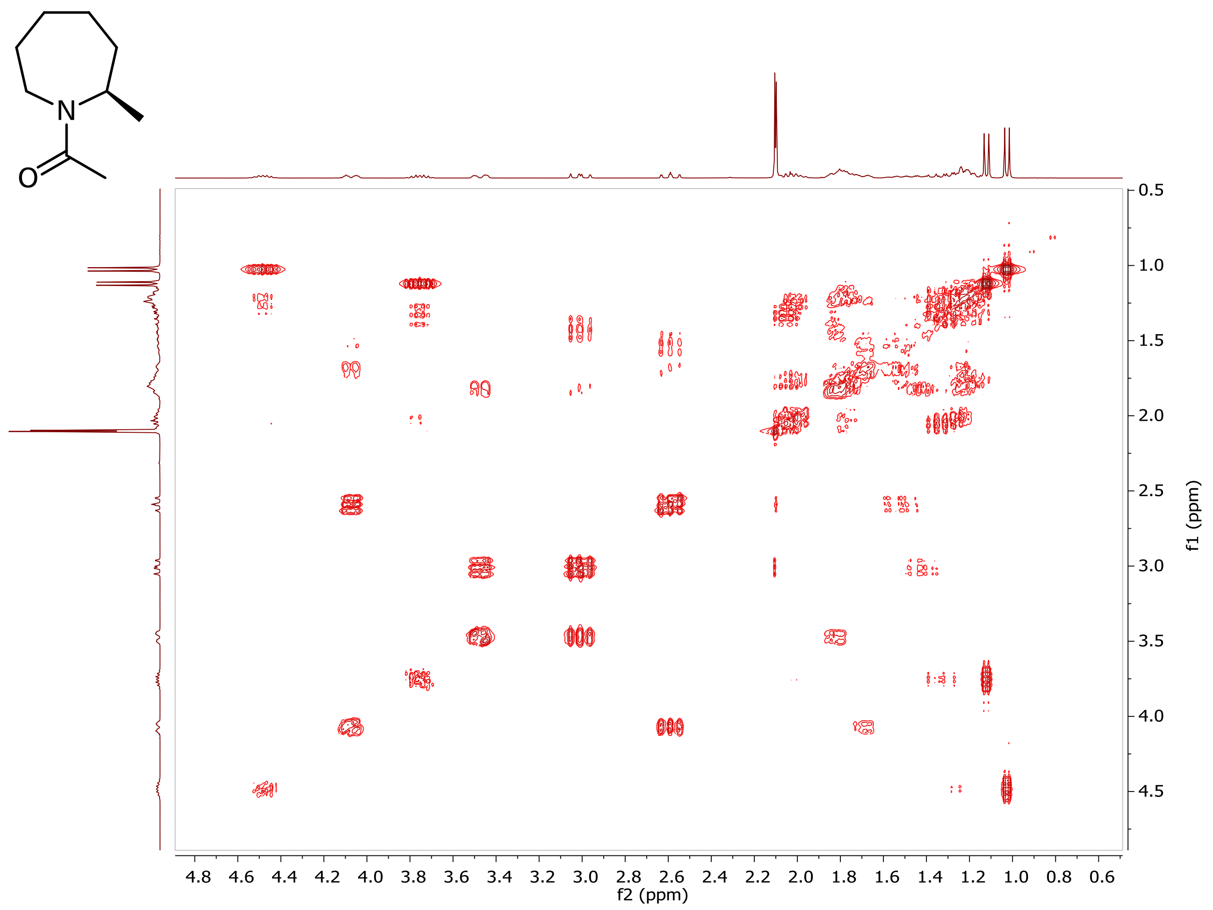

Supplementary Figure S55. COSY-NMR spectrum of (*R*)-1-(2-methylazepan-1-yl)ethanone.

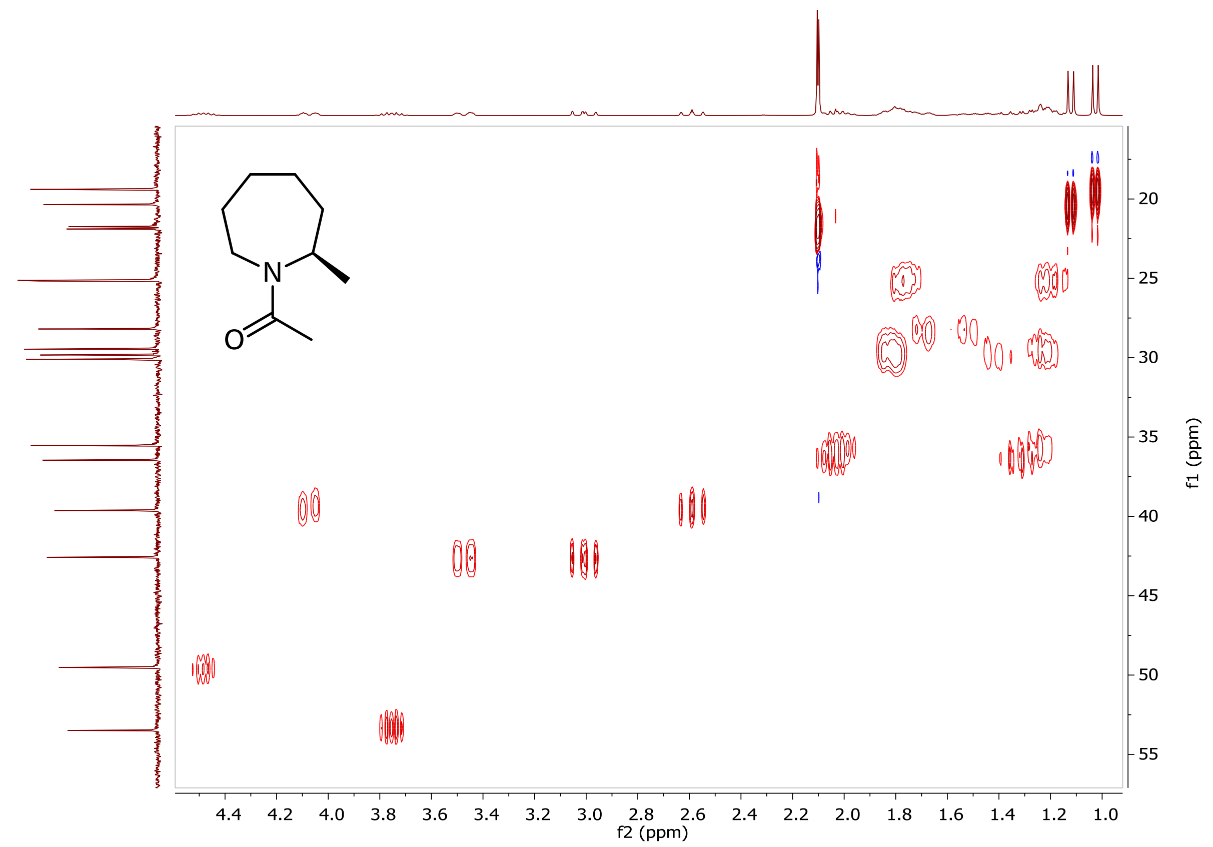

Supplementary Figure S56. HSQC-NMR spectrum of (*R*)-1-(2-methylazepan-1-yl)ethanone.

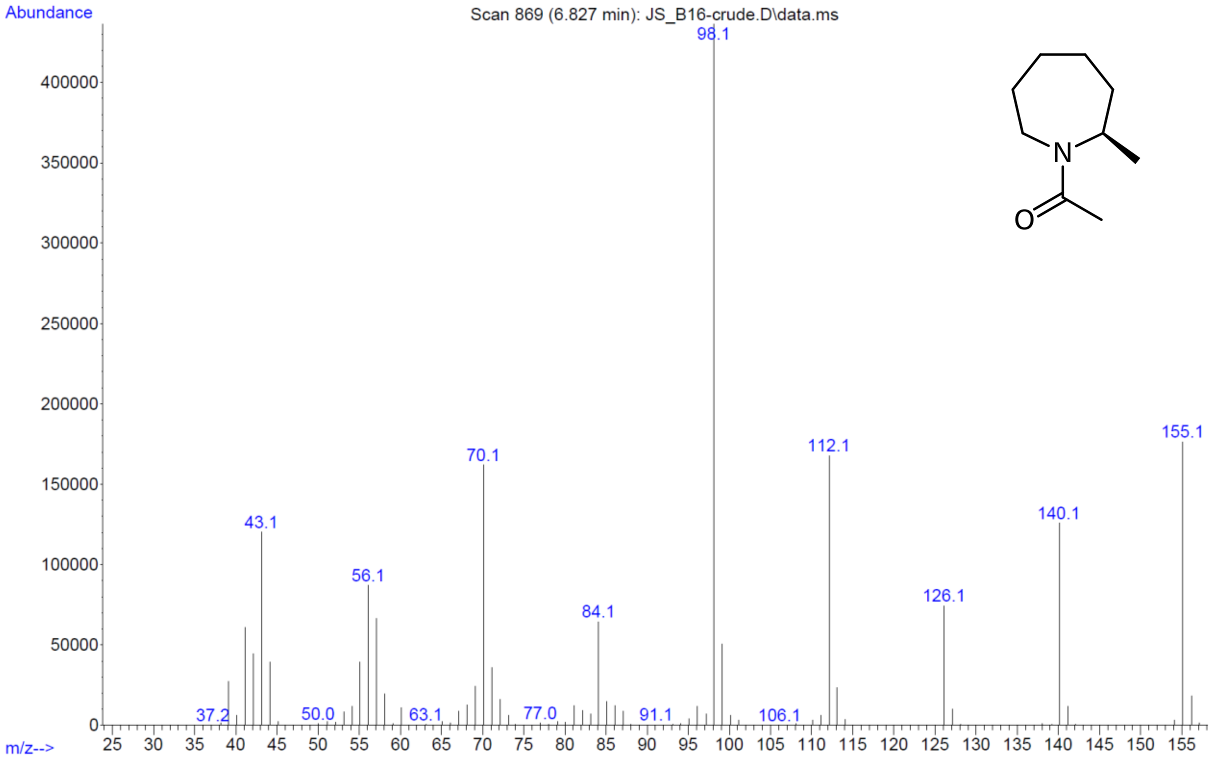

Supplementary Figure S57. MS spectrum of (*R*)-1-(2-methylazepan-1-yl)ethanone.

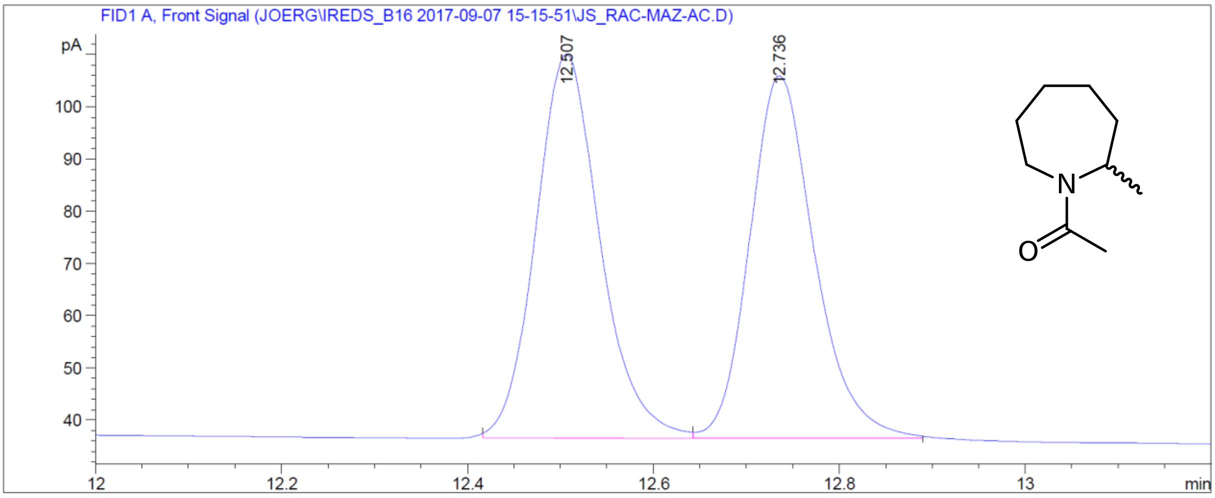

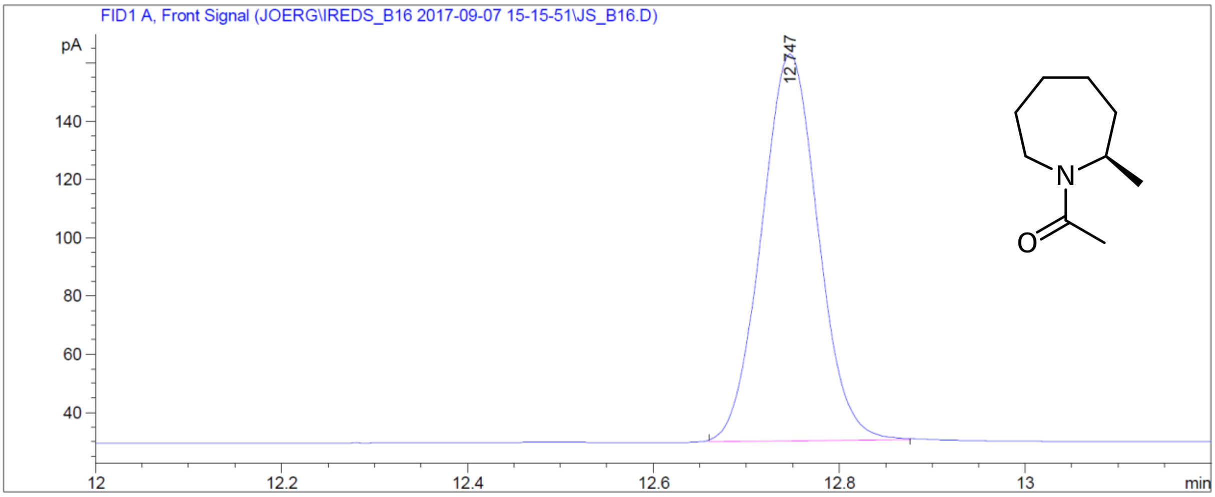

Supplementary Figure S58. Determination of the enantiomeric excess of (*R*)-1-(2-methylazepan-1-yl)ethanone using GC method GC-C1. Racemic standard (top), biotransformation product (bottom).

(*S*)-1-Methyl-1,2,3,4-tetrahydroisoquinoline (*S*)-2g

- ^{1}H-NMR spectrum (Supplementary Figure S59)
- ^{13}C-NMR spectrum (Supplementary Figure S60)
- MS spectrum (Supplementary Figure S61)
- Chiral-phase HPLC chromatogram (Supplementary Figure S62)

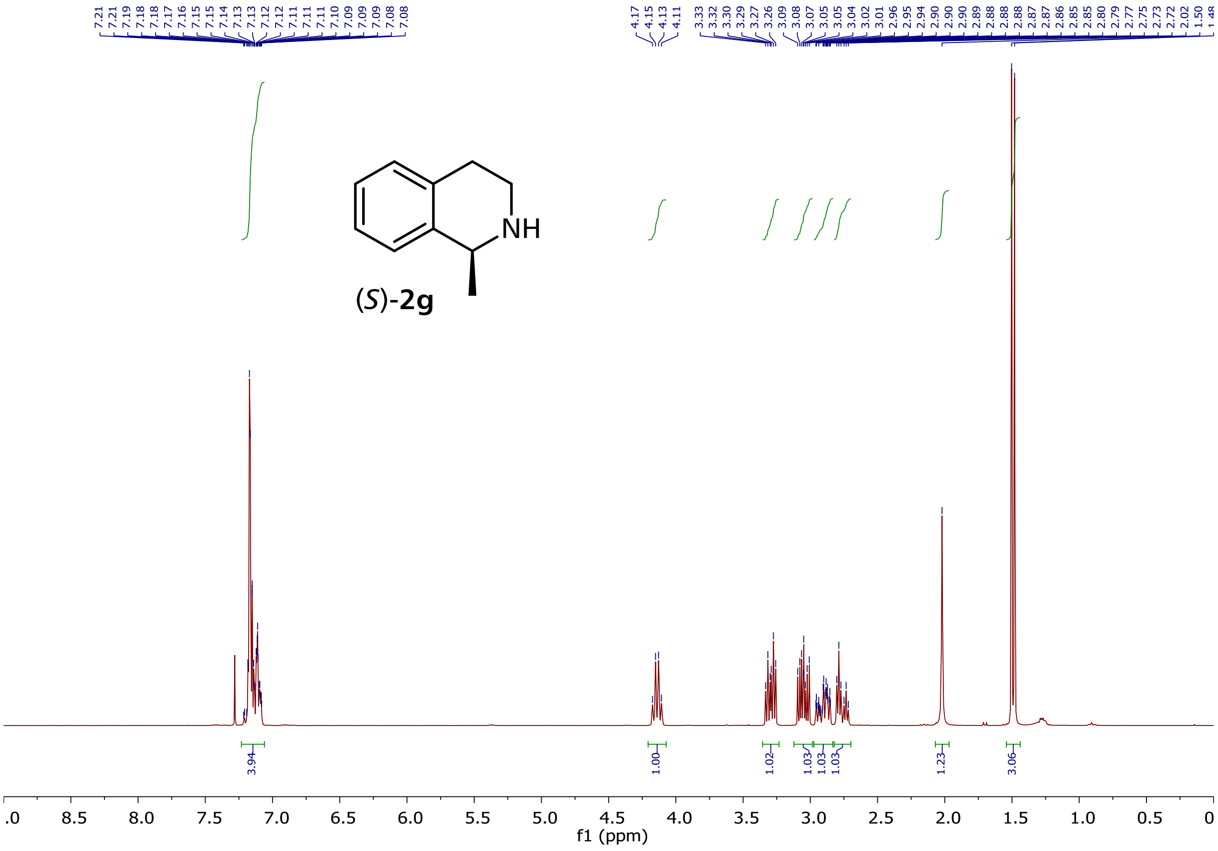

Supplementary Figure S59. ^{1}H-NMR spectrum of (*S*)-**2g**.

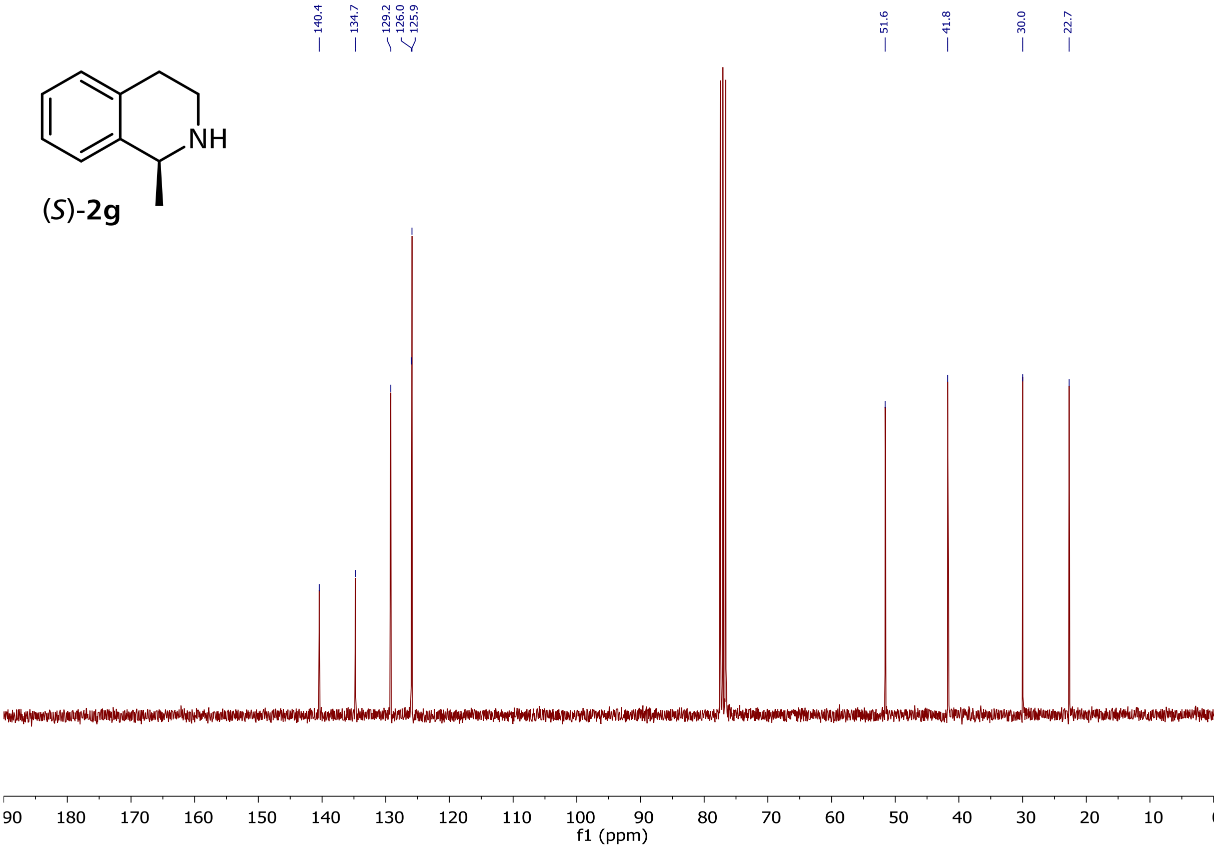

Supplementary Figure S60. ^{13}C-NMR spectrum of (*S*)-**2g**.

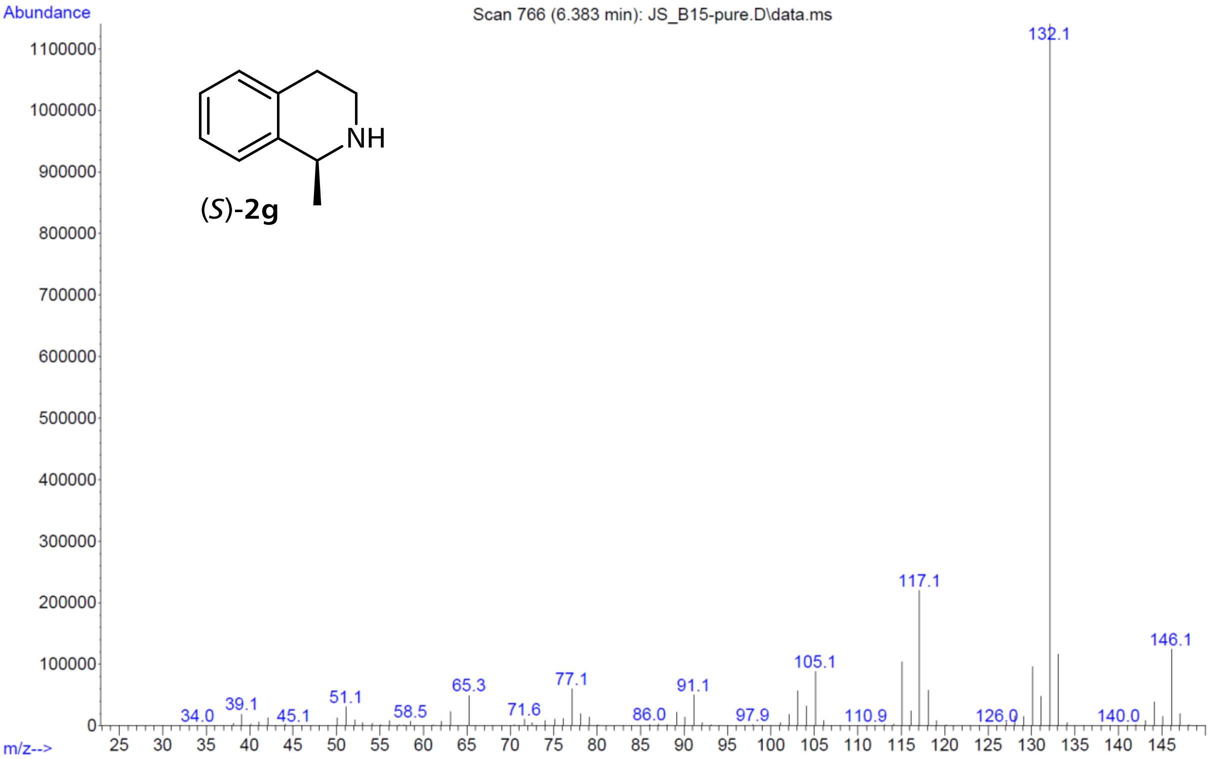

Supplementary Figure S61. MS spectrum of (*S*)-**2g**.

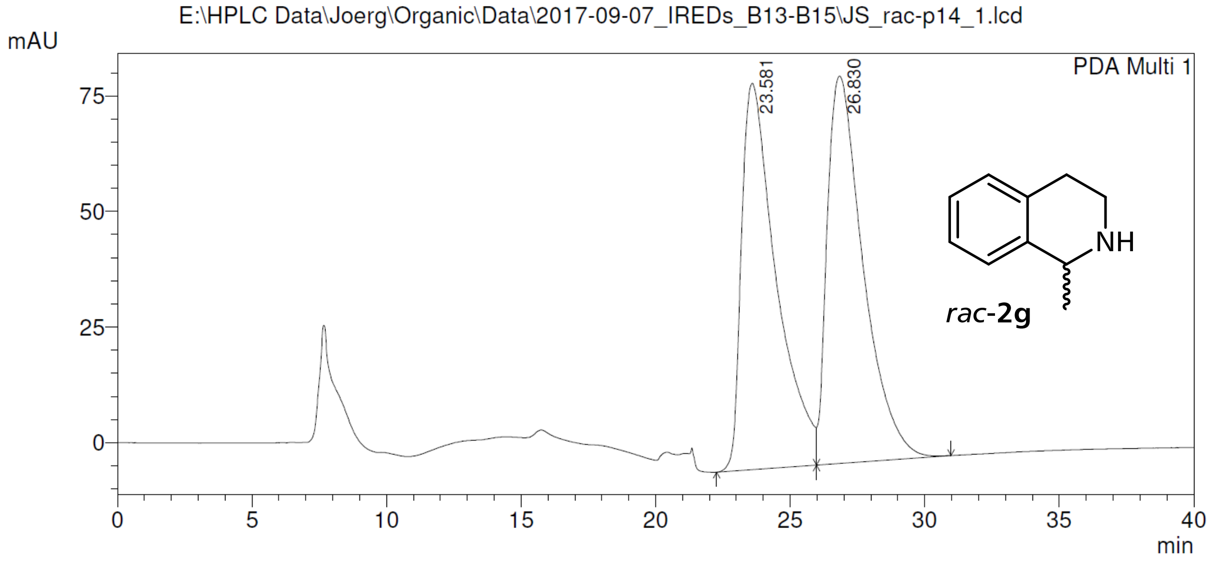

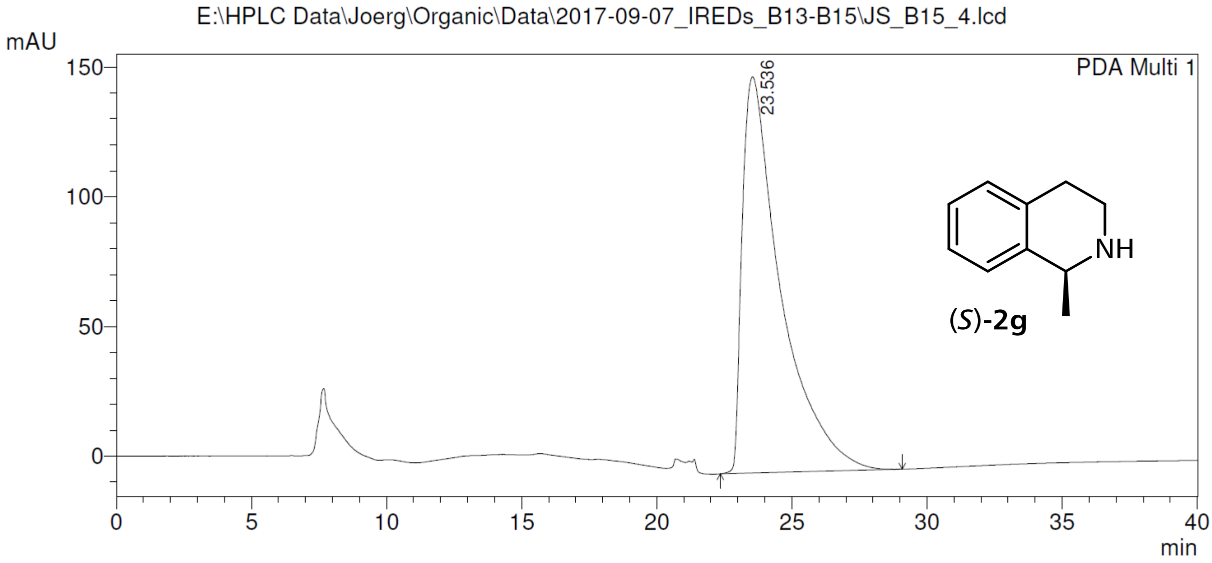

Supplementary Figure S62. Determination of the enantiomeric excess of (*S*)-**2g** using HPLC method HPLC-C1. Racemic standard (top), biotransformation product (bottom).

References

1. T. Huber, L. Schneider, A. Präg, S. Gerhardt, O. Einsle, M. Müller, *ChemCatChem* **2014**, *6*, 2248–2252.

2. D. H. Hua, S. W. Miao, S. N. Bharathi, T. Katsuhira, A. A. Bravo, *J. Org. Chem.* **1990**, *55*, 3682–3684.

3. C. Coindet, A. Comel, G. Kirsch, *Tetrahedron Lett.* **2001**, *42*, 6101–6104.

4. F. Leipold, S. Hussain, D. Ghislieri, N. J. Turner, *ChemCatChem* **2013**, *5*, 3505–3508.

5. N. Pemberton, L. Jakobsson, F. Almqvist, *Org. Lett.* **2006**, *8*, 935–938.

6. H. Song, Y. Liu, Y. Liu, L. Wang, Q. Wang, *J. Agric. Food Chem.* **2014**, *62*, 1010–1018.

7. D. Ghislieri, A. P. Green, M. Pontini, S. C. Willies, I. Rowles, A. Frank, G. Grogan, N. J. Turner, *J. Am. Chem. Soc.* **2013**, *135*, 10863–10869.

8. W.-T. Chan, C. S. Verma, D. P. Lane, S. K.-E. Gan, *Biosci. Rep.* **2013**, *33*, e00086.

9. M. Rodríguez-Mata, A. Frank, E. Wells, F. Leipold, N. J. Turner, S. Hart, J. P. Turkenburg, G. Grogan, *ChemBioChem* **2013,** *14*, 1372–1379.

10. H. Man, E. Wells, S. Hussain, F. Leipold, S. Hart, J. P. Turkenburg, N. J. Turner, G. Grogan, *ChemBioChem* **2015**, *16*, 1052–1059.

11. S. Hussain, F. Leipold, H. Man, E. Wells, S. P. France, K. R. Mulholland, G. Grogan, N. J. Turner, *ChemCatChem* **2015**, *7*, 579–583.

12. Z.-Y. Ding, T. Wang, Y.-M. He, F. Chen, H.-F. Zhou, Q.-H. Fan, Q. Guo, A. S. C. Chan, *Adv. Synth. Catal.* **2013**, *355*, 3727–3735.

13. H. Li, Z.-J. Luan, G.-W. Zheng, J.-H. Xu, *Adv. Synth. Catal.* **2015**, *357*, 1692–1696.

14. T. Shinohara, A. Takeda, J. Toda, T. Sano, *Chem. Pharm. Bull.* **1998**, *46*, 430–433.
